# Characterization and catalytic investigation of fungal single-module nonribosomal peptide synthetase in terpene-amino acid meroterpenoid biosynthesis

**DOI:** 10.1093/jimb/kuad043

**Published:** 2023-12-04

**Authors:** Cheng-Chung Tseng, Li‐Xun Chen, Chi‐Fang Lee, Zhijay Tu, Chun-Hung Lin, Hsiao-Ching Lin

**Affiliations:** Institute of Biological Chemistry, Academia Sinica, Taipei 115, Taiwan R.O.C; School of Pharmacy, National Taiwan University, Taipei 100, Taiwan R.O.C; Institute of Biological Chemistry, Academia Sinica, Taipei 115, Taiwan R.O.C; Institute of Biochemical Sciences, National Taiwan University, Taipei 106, Taiwan R.O.C; Institute of Biological Chemistry, Academia Sinica, Taipei 115, Taiwan R.O.C; Institute of Biochemical Sciences, National Taiwan University, Taipei 106, Taiwan R.O.C; Institute of Biological Chemistry, Academia Sinica, Taipei 115, Taiwan R.O.C; Institute of Biological Chemistry, Academia Sinica, Taipei 115, Taiwan R.O.C; Institute of Biochemical Sciences, National Taiwan University, Taipei 106, Taiwan R.O.C; Institute of Biological Chemistry, Academia Sinica, Taipei 115, Taiwan R.O.C; School of Pharmacy, National Taiwan University, Taipei 100, Taiwan R.O.C; Institute of Biochemical Sciences, National Taiwan University, Taipei 106, Taiwan R.O.C

**Keywords:** Nonribosomal peptide synthetase, Single-module NRPS, Terpene-amino acid meroterpenoids

## Abstract

Hybrid natural products are compounds that originate from diverse biosynthetic pathways and undergo a conjugation process, which enables them to expand their chemical diversity and biological functionality. Terpene-amino acid meroterpenoids have garnered increasing attention in recent years, driven by the discovery of noteworthy examples such as the anthelmintic CJ-12662, the insecticidal paeciloxazine, and aculene A (**1**). In the biosynthesis of terpene-amino acid natural products, single-module nonribosomal peptide synthetases (NRPSs) have been identified to be involved in the esterification step, catalyzing the fusion of modified terpene and amino acid components. Despite prior investigations into these NRPSs through gene deletion or *in vivo* experiments, the enzymatic basis and mechanistic insights underlying this family of single-module NRPSs remain unclear. In this study, we performed biochemical characterization of AneB by *in vitro* characterization, molecular docking, and site-directed mutagenesis. The enzyme reaction analyses, performed with L-proline and daucane/nordaucane sesquiterpene substrates, revealed that AneB specifically esterifies the C10-OH of aculenes with L-proline. Notably, in contrast to ThmA in CJ-12662 biosynthesis, which exclusively recognizes oxygenated amorpha-4,11-diene sesquiterpenes for L-tryptophan transfer, AneB demonstrates broad substrate selectivity, including oxygenated amorpha-4,11-diene and 2-phenylethanol, resulting in the production of diverse unnatural prolyl compounds. Furthermore, site-directed mutagenesis experiments indicated the involvement of H794 and D798 in the esterification catalyzed by AneB. Lastly, domain swapping between AneB and ThmA unveiled that the A‒T domains of ThmA can be effectively harnessed by the C domain of AneB for L-tryptophan transfer, thus highlighting the potential of the C domain of AneB for generating various terpene-amino acid meroterpenoid derivatives.

**One-Sentence Summary:**

The enzymatic basis and mechanistic insights into AneB, a single-module NRPS, highlight its capacity to generate various terpene-amino acid meroterpenoid derivatives.

## Graphical Abstract

**Graphical Abstract:**
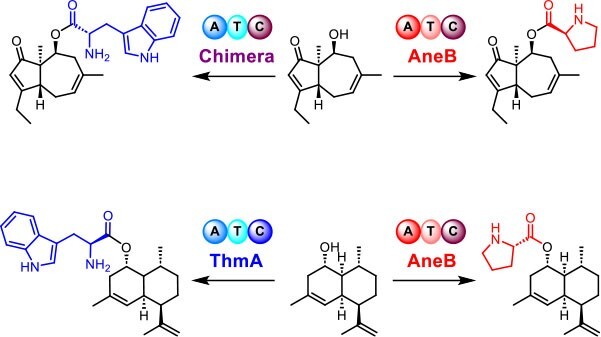

## Introduction

**Fig. 1.**
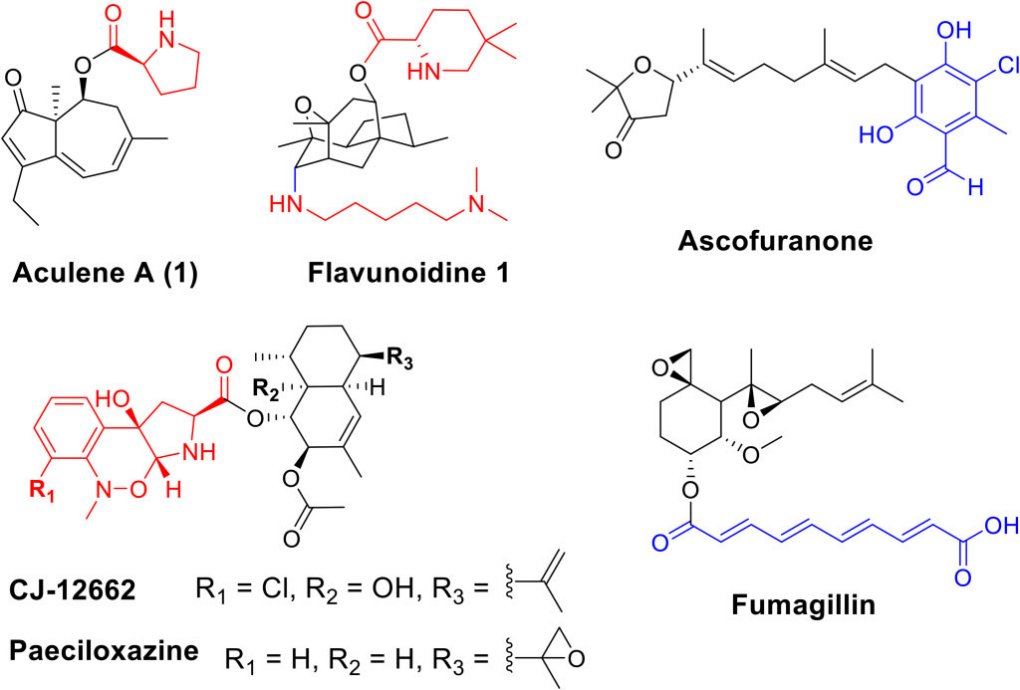
Structure of aculene A (**1**) and representative terpene-amino acid and terpene-polyketide meroterpenoids (color for core biosynthetic enzymes: black, terpene cyclase or prenyltransferase; blue, polyketide synthase (PKS); red, nonribosomal peptide synthetases-related).

Hybrid natural products are compounds with diverse biosynthetic origins that undergo a process of conjugation, allowing for the expansion of their chemical space and biological functions (Fischbach et al., 2008; Walsh & Fischbach, 2010). One well-documented class of hybrid molecules is the terpene-polyketide hybrids, known as meroterpenoids, which include examples like fumagillin (Lin et al., 2013) and ascofuranone (Araki et al., 2019) (Fig. 1). While terpene-polyketide meroterpenoids are extensively studied, terpene-amino acid meroterpenoids, a less common category, have gained increasing attention in recent years due to the discovery of several noteworthy examples. For instance, CJ-12 662 (Cheng et al., 2022), an anthelmintic compound, and paeciloxazine (Yan et al., 2022), an insecticidal agent, both feature a highly oxygenated amorpha-4,11-diene core that is acylated with a pyrrolobenzoxazine carboxylic acid. Another example, flavunoidine (Yee et al., 2020), showcases a tetracyclic oxygenated sesquiterpene connected to dimethylcadaverine and acylated with dimethylpipecolate. Additionally, aculene A (**1**), an oxygenated nordaucane sesquiterpene, is esterified with L-proline (Lee et al., 2019). These unique scaffolds of terpene-amino acid meroterpenoids have sparked significant interest, leading to many total synthesis efforts (Commandeur et al., 2009; Das et al., 2017; Didier et al., 2004; Schwaebisch et al., 2004; Yokokawa et al., 2023). The synthetic strategy for their conjugation typically involves modifying the amino acid or its derivatives with protective groups prior to esterification (Didier et al., 2004; Yokokawa et al., 2023). Subsequently, deprotection steps are undertaken to achieve the total synthesis of these intriguing compounds.

Fungal nonribosomal peptide synthetases (NRPSs) are complex, multifunctional megaenzymes organized into modules that catalyze the sequential synthesis of nonribosomal peptides (NRPs) (Brown et al., 2018; Walsh, 2016). These NRPs are constructed through the stepwise condensation of amino acids and hydroxycarboxylic acid building blocks (Reimer et al., 2019). Each elongation module typically consists of three essential domains: an adenylation (A) domain, a thiolation domain (T), and a condensation (C) domain. A domain selects and activates the incoming building block at the expense of ATP (Walsh et al., 2013). T domain (also known as the peptidyl carrier protein domain, PCP), equipped with a thiol-containing phosphopantetheine arm, serves as a shuttle, tethering the activated amino acid and shuttling the growing NRP intermediate to the next NRPS module. C domain, which contains donor and acceptor binding sites for upstream and downstream PCP-bound substrates, catalyzes amide or ester bond formation (Izoré et al., 2021). The final peptide chain is liberated from the last NRPS module as either a linear or cyclic NRP. This release is typically catalyzed by a terminal condensation (C_T_) domain, which catalyzes hydrolysis or cyclization (Gao et al., 2012; Zhang et al., 2016). In recent years, it has become increasingly evident that the catalytic versatility of C domains reaches well beyond conventional peptide bond formation. C domains have been demonstrated to fulfill an array of highly diverse functions, such as heterocyclization, esterification, chain length control, cycloaddition, Pictet–Spengler cyclization, and Dieckmann condensation (Dekimpe & Masschelein, 2021). Additional structural diversity of the NRPs is achieved through the integration of modification domains within the NRPS itself (McErlean et al., 2019; Zhang et al., 2023). Other tailoring enzymes may act either in trans or after the peptide is released, further enhancing the structural complexity and functional diversity of the final NRP product.

In the biosynthesis of terpene-amino acid meroterpenoids, single-module NRPSs have been identified to be involved in the esterification step to fuse the modified terpene and amino acid portions together. The fusion of terpenes with amino acids offers a means to introduce nitrogen atoms into hydrocarbon-based terpenoids during biosynthesis (Cheng et al., 2022). In aculenes biosynthesis (Lee et al., 2019), AneC terpene cyclase catalyzes the formation of dauca-4,7-diene sesquiterpene (C_15_), and three cytochrome P450 monooxygenases are essential for a stepwise demethylation process, ultimately yielding nordaucane (C_14_). Within the biosynthetic gene cluster, *aneB*, encoding an NRPS, and *aneE*, encoding an α,β-hydrolase, are putative modification enzymes responsible for incorporating L-proline onto the norsesquiterpene core. Deletion of *aneB* resulted in the abolishment of aculenes A (**1**) and B (**2**) and the accumulation of aculene D (**4**) and asperaculane B (**5**) (Fig. 3). These results reveal that AneB catalyzes the transfer of L-proline to **4**, leading to the formation of **2**. On the other hand, in CJ-12 662 and flavunoidine biosynthesis (Cheng et al., 2022; Yee et al., 2020), heterologous expression of corresponding pathway genes with the NRPSs ThmA and FlvI in *Aspergillus nidulans*, respectively, suggesting that ThmA plays a catalytic role in esterifying L-tryptophan to the specific secondary alcohol of 3-*O*-acetyl-amorpha-4,11-diene-1,2,3-triol and FlvI is involved in esterifying dimethylpipecolate to dimethylcadaverine (Fig. 1). Despite the function of these NRPSs have been investigated by gene deletion or *in vivo* experiments, the enzymatic basis and mechanistic understanding of this family of single-module NRPS remain unclear.

In this study, we characterized the function of AneB through *in vitro* assays, molecular docking, and mutagenesis. AneB was found to selectively esterify the C10-OH of daucane/nordaucane sesquiterpene with L-proline. In contrast to ThmA, which is specific to oxygenated amorpha-4,11-diene sesquiterpenes for L-tryptophan transfer, AneB showed broad substrate selectivity, accepting various substrates, including oxygenated amorpha-4,11-diene and 2-phenylethanol, resulting in diverse unnatural prolyl compounds. Site-directed mutagenesis pinpointed the importance of H794 and D798 in AneB's esterification activity. Domain swapping between AneB and ThmA revealed the potential of the C domain of AneB for generating terpene-amino acid meroterpenoid derivatives.

## Materials and Methods

### Strains and Culture Conditions

*Saccharomyces cerevisiae* BJ5464-NpgA and *S. cerevisiae* RC01 (BJ5464-NpgA harboring *AtCPR*) (Tang et al., 2015) were cultured in Yeast Extract-Peptone-Dextrose (YPD) medium (containing 10 g yeast extract, 20 g peptone, and 1% glucose in 1 L) at 28°C and 230 rpm. *S. cerevisiae* BJ5464-NpgA for the seed cultures was grown in yeast synthetic drop-out medium. The *Aspergillus aculeatus* ATCC 16,872 strain (=BCRC 32 190) was obtained from the Bioresource Collection and Research Center in Taiwan, R.O.C. For the extraction of genomic DNA and total RNA, *A. aculeatus* was cultivated in Czapek yeast extract liquid medium (containing 30 g/L sucrose, 5 g/L yeast extract, 3 g/L NaNO_3_, 1 g K_2_HPO_4_, 0.5 g MgSO_4_·7H_2_O, 0.5 g/L KCl, 0.01 g/L FeSO_4_‧7H_2_O, 0.01 g/L ZnSO_4_‧7H_2_O, 0.005 g/L CuSO_4_‧5H_2_O) at 28°C for 4 days and 7 days. For long-term storage, the mycelia of *A. aculeatus* were suspended in a 33% glycerol stock and stored at −80°C.

### Chemical Analysis

Liquid chromatography-diode array detector-mass spectrometry (LC-DAD-MS) analysis was conducted using a Shimadzu 2020 liquid chromatography-tandem mass spectrometry system with a Kinetex® 2.6 μm Polar C18 100 Å 2.1 × 100 mm column. Electrospray ionization (ESI) was employed to generate ions in both positive and negative modes. The elution method consisted of a linear gradient from 5 to 95% acetonitrile/water with 0.05% formic acid over 10 min, followed by 95% acetonitrile/water for 4 min, with a flow rate of 0.5 mL/min. All solvents and chemicals used were of analytical grade. Authentic compounds **1**‒**5, 7**, and **8**, obtained in previous studies, were used as standards for comparison in chemical analyses. For nuclear magnetic resonance (NMR) analysis, ^1^H, ^13^C, and 2D NMR spectra were recorded on either a Bruker AvanceTM III 600 MHz or a Bruker NEO 500 MHz spectrometer equipped with a 5 mm dual cryoprobe. These analyses were performed at the High Field NMR Center, Institute of Biomedical Sciences, Academia Sinica.

For structural analysis by liquid chromatography-tandem mass spectrometry (LC-MS/MS), we conducted ion mobility/time-of-flight mass spectrometry using a timsTOF Pro instrument manufactured by Bruker Daltonics in Bremen, Germany. This instrument was equipped with an online electrospray ionization source and coupled to an ultra-performance liquid chromatography system, specifically the LC-40D X3 by Shimadzu in Kyoto, Japan. Chromatographic separations were executed on a Waters Acquity UPLC BEH C18 column measuring 2.1 mm in diameter and 100 mm in length, with a particle size of 1.7 μm. Mobile phase A consisted of acetonitrile modified with 0.1% (v/v) formic acid, while mobile phase B comprised water with 0.1% (v/v) formic acid. A gradient profile was employed, transitioning from 70% B to 5% B over a 6-min interval. In both analyses, the flow rate was maintained at 0.30 mL/min, and the sample injection volume was set at 5 μL. Data acquisition occurred in the positive-ion mode, adhering to the subsequent parameters: a needle voltage of 3,500 V, source temperature at 30°C, dry temperature set at 200°C, and a dry gas flow rate of 3 L/min. Precursors for data-dependent acquisition were isolated within a range of ± 1 Th and subjected to tandem mass spectrometer (MS/MS) fragmentation with the application of collision energy. Structural elucidation was facilitated by utilizing Mass Frontier 8.0 software, developed by Thermo Fisher Scientific in Waltham, United States, to obtain the necessary fragments.

### Heterologous Reconstitution and Biotransformation by *S. Cerevisiae*

Different combinations of plasmids were co-transformed into *S. cerevisiae* or *S. cerevisiae* RC01 using the Frozen-EZ Yeast Transformation II Kit™ from Zymo Research. The transformants were then inoculated into 2.0 mL of yeast drop-out medium and grown for 72 hr with constant shaking at 230 rpm. Next, 3 μL aliquots of the seed cultures were used to inoculate 3.0 mL of YPD medium for an additional 3 days of growth.

For biotransformation, the cultures were concentrated to 1 mL by removing 2 mL of the medium after 3 days of cultivation. Aculene D (**4**) or asperaculane B (**5**) was then added to the concentrated culture, each at a final concentration of 0.1 mM. These cultures were allowed to continue to grow for an additional 2 days. Subsequently, for LC-DAD-MS analysis, the small-scale cultures were harvested by centrifugation at 13,000 rpm for 10 min to separate the cell and medium components. The cell-free medium was extracted using ethyl acetate, while the cell pellet was extracted with acetone and sonicated for 30 min. Solvent removal was performed under vacuum, and the crude extract was dissolved in MeOH before being analyzed by LC-DAD-MS. Details of the *S. cerevisiae* transformants are listed in Supplementary Table S3.

### Overexpression and Purification of AneB and AneB T‒C Domain from *S. Cerevisiae*

The *S. cerevisiae* strain expressing pXW55H-AneB or pXW55-AneB T-C domain was cultivated in 2 Lof YPD medium for 3 days with constant shaking at 230 rpm at 28°C. After 3 days of cultivation, yeast cells were harvested by centrifugation at 3,750 rpm for 15 min and suspended in 25 mL of yeast lysis buffer (50 mM NaH_2_PO_4_,150 mM NaCl, 10 mM imidazole, pH 8.0). Yeast cell disruption was carried out using a NanoLyzer N-2 High-Pressure Homogenizer at 23 kpsi for two cycles. The cell lysates were then centrifuged at 20,000 rpm for 1 hr to precipitate cell debris. The supernatant was filtered through a 0.2 μm filter to further remove any remaining cell debris. Next, 2 mL of Ni-NTA (nickel-nitrilotriacetic acid) agarose resin (Thermo Fisher Scientific) was added to the lysate and incubated for 6 hr at 4°C. The recombinant His-tag fusion protein was purified using an affinity open column. Non-binding proteins were removed using a 10 mM imidazole elution buffer (500 mM NaCl, 20 mM Tris-HCl, 20 mM imidazole, pH 7.9). The His-tagged fusion protein was then eluted using a 250 mM imidazole elution buffer (500 mM NaCl, 20 mM Tris-HCl, 250 mM imidazole, pH 7.9). The purified recombinant proteins were concentrated and exchanged into a storage buffer (50 mM Tris-HCl, pH 7.5, 100 mM NaCl, 10% glycerol) using an Amicon Ultra-30 Centrifugal Filter Unit and stored at ‒80°C.

### Overexpression and Purification of AneB C Domain from *Escherichia Coli*

To express the protein in *Escherichia coli*, the pColdI-MBP-AneB C domain plasmid was transformed into *E. coli* BL21 (DE3) and cultivated in 5 mL of LB broth with 100 μg/mL ampicillin at 37°C and 230 rpm overnight. The overnight seed culture was then inoculated into 1 liter of LB broth (containing 100 μg/mL ampicillin) at 37°C and 230 rpm until reaching an OD600 of 0.4–0.6. Subsequently, the 1-L culture was induced with a final concentration of 100 μM isopropyl β-D-1-thiogalactopyranoside and incubated at 16°C and 180 rpm overnight. *Escherichia coli* BL21 cells were harvested by centrifugation at 3,750 rpm for 15 min and resuspended in 50 mL of maltose binding protein (MBP) binding buffer (200 mM NaCl, 20 mM Tris-HCl, 1 mM EDTA, 10 mM imidazole, pH 7.5). The cells were lysed using a NanoLyzer N-2 High-Pressure Homogenizer (twice at 18 kpsi), and the lysate was then centrifuged at 20,000 rpm for 1 hr to remove cell debris. The cell lysate was mixed with 2 mL of MBP resin (Cytiva, Dextrin Sepharose^TM^ High Performance) and stirred at 4°C for 6 hr. The recombinant MBP-tagged fusion protein was purified using an affinity open column. A total volume of 50 mL of MBP binding buffer was loaded to remove non-binding proteins, and the MBP-fused protein was subsequently eluted with 20 mL of MBP binding buffer containing 10 mM maltose. The purified MBP-fused protein was digested with tobacco etch virus (TEV) protease (0.01 mg of protease for 1.0 mg of protein) to remove the MBP tag. Following TEV protease digestion, the MBP tag and TEV protease were removed using a Ni-NTA agarose resin open column. The purified proteins were concentrated and exchanged into a storage buffer (50 mM Tris-HCl, pH 7.5 100 mM NaCl, 10% glycerol) using an Amicon Ultra-30 Centrifugal Filter Unit and stored at ‒80°C.

### *In Vitro* Assay of AneB and T‒C Domain and C Domain of AneB

The enzymatic reaction of AneB was conducted in a reaction buffer containing 50 mM Tris-HCl (pH 7.0), 10 mM MgCl_2_, 10 mM ATP, and the reactants, which included 1 mM L-proline along with 0.5 mM each of **3**‒**5, 7, 9**, and **12**. Additionally, 10 μM of the enzyme AneB was used. The enzymatic reaction of truncated AneB (AneB T‒C domain and AneB C domain) was performed in a buffer consisting of 50 mM Tris-HCl (pH 7.0). The reactants included 5 mM L-proline-*S*-*N*-acetylcysteamine (L-prol-SNAC) and 0.5 mM **4**. Truncated AneB enzyme (10 μM) was added to the reaction. Negative controls were established by using boiled enzymes, which were subjected to heating at 95°C for 10 min. Following incubation at 28°C for 17 hr, the reaction mixtures were quenched and extracted using *n*-butanol. The organic solvents were subsequently evaporated under reduced pressure. The resulting extracts were dissolved in MeOH and then subjected to analysis by LC-DAD-MS.

### Isolation of Asperaculane H (6)

The *S. cerevisiae* strain expressing pXW55-AneB and pXW06-AneE was cultivated in 2 Lof YPD medium for 3 days with continuous shaking at 230 rpm and a temperature of 28°C. After 3 days, the culture was centrifuged at 3,750 rpm for 15 min. Subsequently, 850 mL of the broth was removed, and the cell pellets were resuspended. The remaining 150 mL of the concentrated culture was supplemented with 500 µL of a 100 mM solution of **5** and then incubated at 28°C with continuous shaking at 230 rpm for 1 day. The cells and medium were separated by another round of centrifugation at 3750 rpm for 15 min. The cellular part was extracted with acetone three times, while the medium part was partitioned with ethyl acetate three times. The organic solvents from both parts were subsequently removed under reduced pressure. The residual water from the acetone extract and medium was combined and subjected to partitioning with an equivalent volume of *n*-butanol three times. The *n*-butanol extract and ethyl acetate extract were combined and further separated using a Sephadex LH-20 column eluted with a methanol:water mixture (8:2) at a flow rate of 0.66 mL/min. Fraction 2 was then subjected to additional purification using a preparative high performance liquid chromatography (HPLC) column (Galaksil EF-C18M, 5 μm, 120 Å, 250 × 30 mm) eluted by 15% acetonitrile/water to 28% acetonitrile/water with 0.05% formic acid and a semi-preparative HPLC column (Luna, 5 μm, 18C(2), 250 × 10 mm) from 5% acetonitrile/water to 65% acetonitrile/water with 5% methanol, ultimately yielding compound **4** (2.2 mg).

### Isolation of Proline Phenethyl Ester (10)

The *S. cerevisiae* strain expressing pXW55H-AneB was cultivated in 3 Lof YPD medium for 5 days at 28°C with constant shaking at 230 rpm. The culture medium was partitioned three times with ethyl acetate and concentrated under reduced pressure, resulting in an ethyl acetate crude extract (1.66 g). The extracts from the culture medium were further isolated using a medium-pressure liquid chromatography column. A gradient elution was performed from 10% acetonitrile/water to 30% acetonitrile/water with 0.05% formic acid over 60 min, yielding fraction F1 (226.44 mg). Fraction F1 was subsequently subjected to purification using a preparative HPLC column (Galaksil EF-C18M, 5 μm, 120 Å, 250 × 30 mm) with a gradient elution from 10% acetonitrile/water to 20% acetonitrile/water with 0.1% formic acid over 60 min, leading to the isolation of fraction F1-1 (4.9 mg). Finally, fraction F1-1 was further purified using a semi-preparative HPLC column (Luna, 5 μm, 18C (2), 250 × 10 mm). A gradient elution from 20% acetonitrile/water to 30% acetonitrile/water with 0.1% trifluoroacetic acid over 60 min resulted in the isolation of compound **10** (1.3 mg).

### Isolation of Amorpha-4,11-Diene (11) and Amopha-4,11-Diene-2-Ol (12)

The *S. cerevisiae* strain expressing pXW55H-ThmB was cultivated in 4 L of YPD medium for 4 days at 28°C with constant shaking at 230 rpm. The cells were subjected to acetone extraction six times. The acetone extracts were concentrated under reduced pressure and then partitioned with hexanes and water six times. The hexanes fraction was concentrated under reduced pressure to yield a hexanes crude extract (223.6 mg). The hexanes-soluble components were separated by a silica gel 60 column using 100% hexane as the eluent, affording compound **11** (9.41 mg, colorless oil). Additionally, the *S. cerevisiae* strain expressing pXW55H-ThmB and pXW06H-ThmI was cultivated in 4 L of YPD medium for 7 days at 28°C with constant shaking at 230 rpm. The cells were extracted with acetone six times. The acetone extracts were concentrated under reduced pressure and then partitioned with ethyl acetate and water six times. The ethyl acetate fraction was concentrated under reduced pressure to obtain an ethyl acetate crude extract (455.27 mg). The culture medium was also subjected to ethyl acetate extraction three times, and the resulting extract was concentrated under reduced pressure to give an ethyl acetate crude extract (1.34 g). The extracts from both the culture medium and cells were isolated using a preparative HPLC column (Galaksil EF-C18M, 5 μm, 120 Å, 250 × 30 mm). A gradient elution with 70% acetonitrile/water containing 0.05% formic acid resulted in the isolation of fraction F1 (32.2 mg). Fraction F1 was further purified using a semi-preparative HPLC column (Luna, 5 μm, 18C(2), 250 × 10 mm) with 70% acetonitrile/water containing 0.05% formic acid, leading to the isolation of compound **12** (1.7 mg, colorless oil).

### Isolation of Tryptophan Phenethyl Ester (16)

The *S. cerevisiae* strain expressing pXW55H-ThmA (A domain)-AneB (TC domain) was cultured in 2 L of YPD medium for 3 days at 28°C with constant shaking at 230 rpm. The cells were subjected to acetone extraction six times. The acetone extracts were concentrated under reduced pressure and subsequently partitioned with ethyl acetate and water for six cycles. The ethyl acetate phase was concentrated under reduced pressure to yield an ethyl acetate crude extract (192.4 mg). Additionally, the culture medium was partitioned with ethyl acetate three times and concentrated under reduced pressure to provide an ethyl acetate crude extract (556.29 g). The extracts obtained from both the culture medium and the cells were separated using a preparative HPLC column (Galaksil EF-C18M, 5 μm, 120 Å, 250 × 30 mm). A gradient elution from 30% acetonitrile/water to 35% acetonitrile/water, containing 0.1% trifluoroacetic acid over 60 min, resulted in the isolation of fraction F1 (5.7 mg). Fraction F1 was further purified using a semi-preparative HPLC column (Luna, 5 μm, 18C(2), 250 × 10 mm) with 35% acetonitrile/water containing 0.1% trifluoroacetic acid, leading to the isolation of compound **16** (0.8 mg).

## Results

### Functional Characterization of AneB *In Vivo* and *In Vitro*

**Fig. 2.**
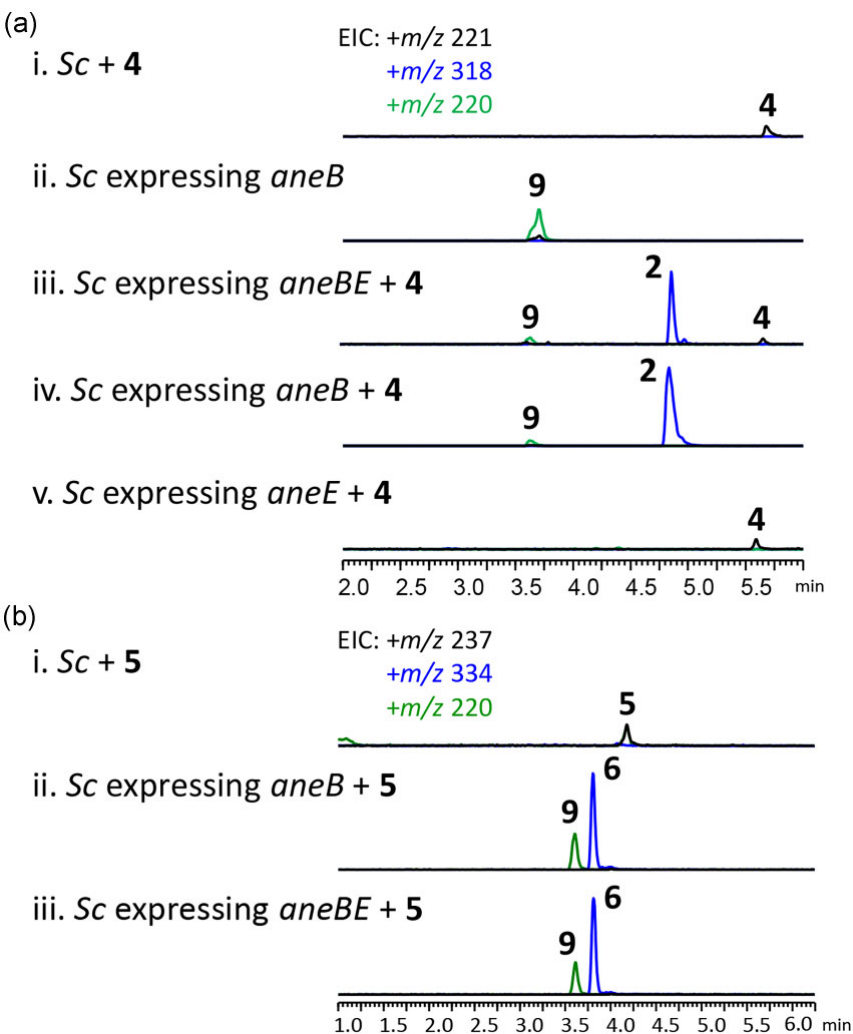
LC-MS analysis of *Saccharomyces cerevisiae* control and *S. cerevisiae* expressing *aneB, aneE*, or *aneB*/*E* supplemented with (a) **4** and (b) **5**.

We first set out to determine the roles of the enzymes in the conjugation of aculenes and L-proline. A single-module NRPS (AneB) and an α,β-hydrolase (AneE) are encoded on the *ane* gene cluster in the genome of *A. aculeatus* ATCC16872. AneB NRPS comprises A, T, and C domains, where the C domain contains a cd19545 FUM14_C_NRPS-like conserved domain but exhibits no significant sequence identity to C domain of the ester-bond-forming *Fusarium verticillioides* FUM14 protein (accession number Q8J2Q6.2, with cd19536 conserved domain). We hypothesized that AneB is responsible for activating L-proline, while AneE may catalyze the transfer or release of L-proline from AneB to the sesquiterpene moiety. Previous gene inactivation experiments on *aneB* and *aneE* genes showed that they are involved in the hybridization process between aculene D (**4**) and L-proline to give aculene B (**2**) (Lee et al., 2019). To confirm the function of AneB and AneE, we cloned intron-free *aneB* and *aneE* from *A. aculeatus* ATCC16872 and expressed them in *S. cerevisiae* strain BJ5464-NpgA. In both strains expressing *aneB* and co-expressing *aneB*/*E*, and upon supplementation with compounds **4** and asperaculane B (**5**), we observed the production of compounds **2** and **6**, respectively (Fig. 2) Compound **6** was structurally characterized as a new compound and named asperaculane H (Supplementary Figs. S7‒S12; Supplementary Table S4). These results suggested that AneB alone possesses the catalytic capability to incorporate L-proline into nordaucane sesquiterpenes.

**Fig. 3.**
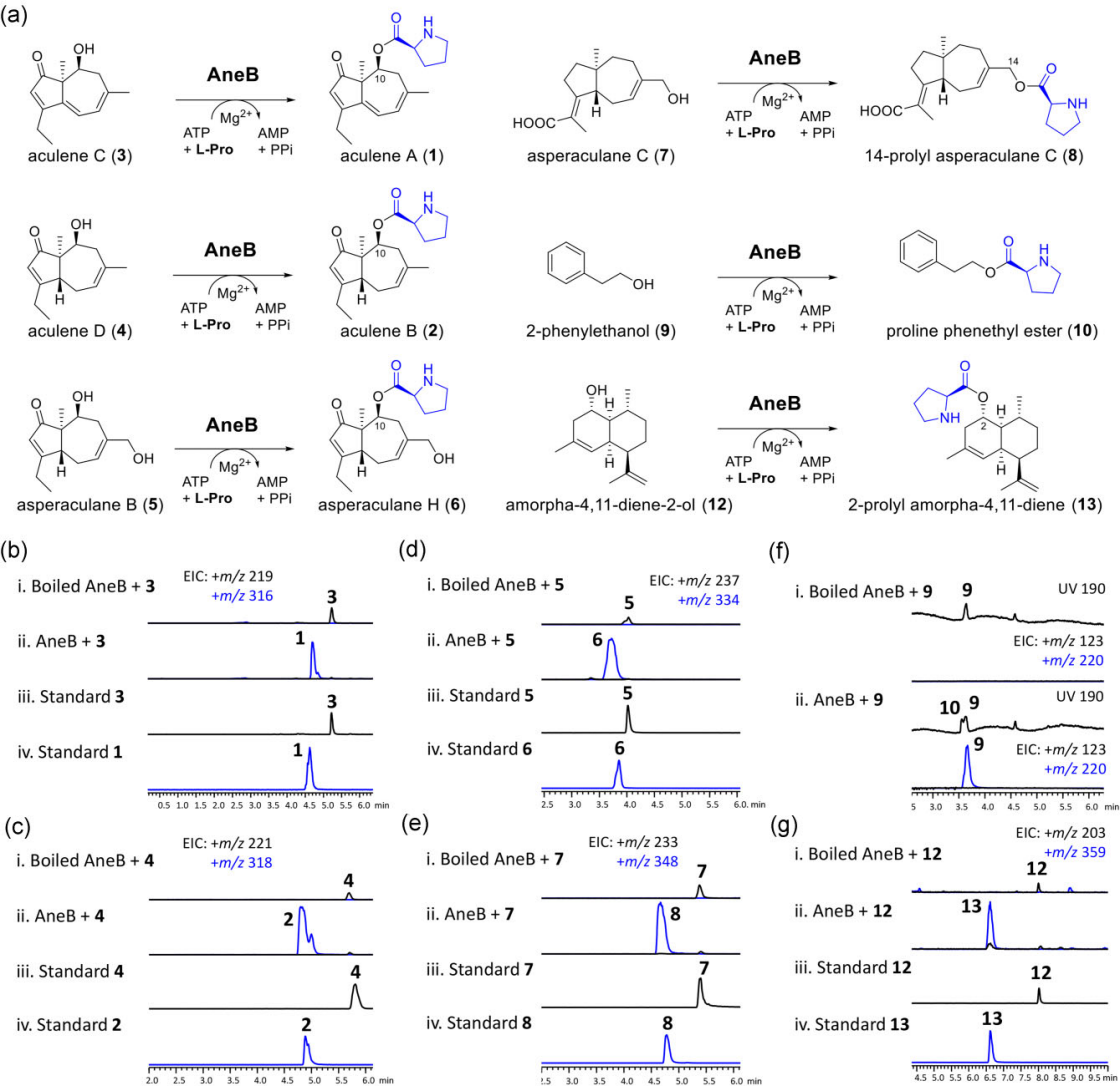
Verification of function of AneB. (a) Structure of daucane/nordaucane sesquiterpenes, 2-phenylethanol, and amorpha-4,11-diene-2-ol for AneB. LC-MS analysis of *in vitro* assays of AneB with substrates (b) **3**, (c) **4**, (d) **5**, (e) **7**, (f) **9**, and (g) **12**.

To verify the function of AneB, we purified recombinant proteins from *S. cerevisiae* strain BJ5464-NpgA (Ma et al., 2009), which has had its vacuolar proteases removed and is equipped with a phosphopantetheinyl transferase (NpgA) capable of priming the apo forms of NRPS carrier domains (Supplementary Fig. S1). When AneB was incubated with aculene C (**3**) or **4**, L-proline, ATP, and MgCl_2_, we observed the formation of **1** and **2**, respectively (Fig. 3b and 3c). These results demonstrate that AneB catalyzes esterification, enabling the incorporation of L-proline into **3** and **4**, ultimately yielding **1** and **2**, respectively. To explore the regioselectivity of AneB, we conducted tests with **5**, which contains an additional 14-hydroxyl group compared to **4**. In this case, we exclusively observed the production of **6** (Fig. 3d). This result indicates that AneB selectively esterifies the C10-OH position rather than the C14-OH position of the nordaucane sesquiterpene when reacting with L-proline. In addition, we tested AneB's activity with the daucane sesquiterpene, asperaculane C (**7**), which possesses a C14-OH group. The production of 14-prolyl asperaculane C (**8**) was observed (Fig. 3e), indicating that AneB exhibits catalytic activity in installing L-proline at the C14-OH position of **7**.

### Characterization of Functional Domains and Catalytic Residues

Fungal NRPSs employ their C_T_ domains to catalyze the cyclization and release of peptide substrates linked to the T-domain partner of the C_T_ domain (Gao et al., 2012; Zhang et al., 2016). This process relies on a catalytic histidine that deprotonates the amine nucleophile, enabling it to initiate cyclization by attacking the thioester carbonyl group. The reaction requires specific protein–protein interactions with the upstream T domain, which facilitate catalysis (Gao et al., 2013; Haynes et al., 2012). In the context of AneB catalysis of the terpene-amino acid conjugation reaction, we hypothesized that the AneB C domain might recognize the terpene moiety and aid in nucleophilic attack, possibly involving the hydroxyl group on the terpene for esterification. To understand the mechanism catalyzed by AneB, we conducted molecular modeling, docking analysis, and site-directed mutagenesis to unravel the roles played by the T and C domains of AneB and the catalytic residues involved.

**Fig. 4.**
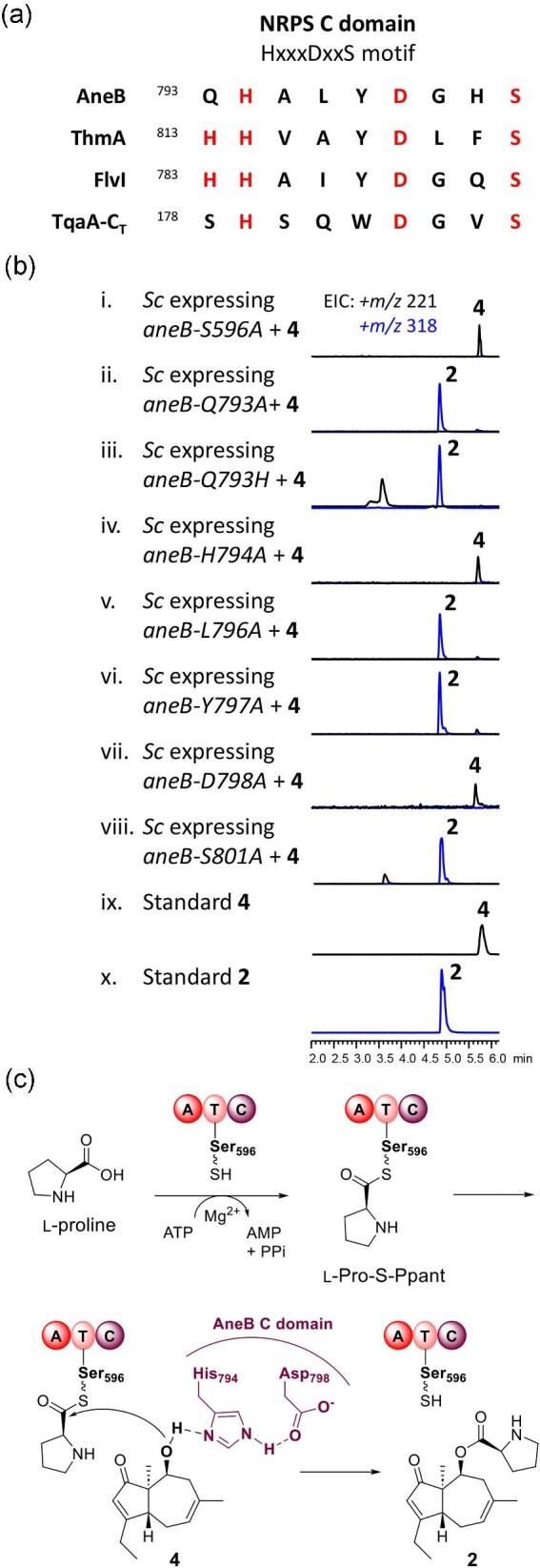
Verification of AneB active site motifs. (a) Alignment of amino acid sequences of the conserved motifs of the C domain of single-module NRPSs, AneB, ThmA, and FlvI, and the C_T_ domain of TqaA. (b) LC-MS analysis of yeast expressing the AneB mutants S596A, Q793A, Q793H, H794A, L796A, Y797A, D798A, and S801A. (c) Proposed mechanism of AneB-mediated esterification of **2** and L-proline to give **1**.

First, when disabling the T domain by mutating the key residue S596 (Supplementary Fig. S2), which serves as the attachment site for the phosphopantetheinyl group, the abolishment of **1** in yeast expressing *aneB*-S596A was observed when **4** was supplied (Fig. 4b). Second, we performed a sequence alignment of the C domains from AneB, ThmA, and FlvI and aligned them with the C_T_ domain of TqaA (Gao et al., 2012; Zhang et al., 2016) (Fig. 3a). The latter enzyme catalyzes the formation of a 10-membered macrocycle from the tripeptidyl thioester anthranilate, followed by a spontaneous intramolecular annulation to yield fumiquinazoline F. The C domain of AneB contains the QHXXXDXXS motif, differing from the HHXXXDXXS motif found in other NRPS C domains. To verify the key residues involved in the condensation reaction, we conducted site-directed mutagenesis on Q793, H794, L796, Y797, D798, and S801, replacing them with alanine. Functional analysis using *S. cerevisiae* BJ5464-NpgA expressing the mutants and supplemented with **4** showed that H794A and D798A mutations abolished the formation of **2**, while the other mutants retained AneB activity (Fig. 3b). Additionally, we constructed the Q793H mutant, but the production of **2** was still observed, suggesting that the first histidine in the conserved motif is not involved in esterification to form **2** (Fig. 4b).

We generated a protein model of AneB using AlphaFold2 (Jumper et al., 2021) and docked it with compounds **4, 5**, and **7**, respectively, using AutoDock Vina (Eberhardt et al., 2021; Trott & Olson, 2010) (Supplementary Fig. S3). A pocket site containing residues of the conserved QHXXXDXXS motif was observed, indicating the substrate-binding region. The resulting structures showed that C domain of AneB can accommodate **4** or **5**, with the C-10 hydroxyl group of **4** and **5** positioned near the H794 residue. On the other hand, the hydroxyl group at C-13 of **7** is also in proximity to the H794 residue. Based on these findings as well as the mutagenesis results, it is likely that the D798 and H794 residues in AneB function as an acid−base pair for proton transfer through an acid−base nucleophilic mechanism. We propose a plausible mechanism in which D798 forms a hydrogen bond with H794, allowing H794 to act as a general base, thus activating the nucleophile C10-OH in **4**. This activation facilitates its attack on the thioester bond of tethered L-proline on the T domain, ultimately leading to esterification and the formation of **2** (Fig. 4c).

**Fig. 5.**
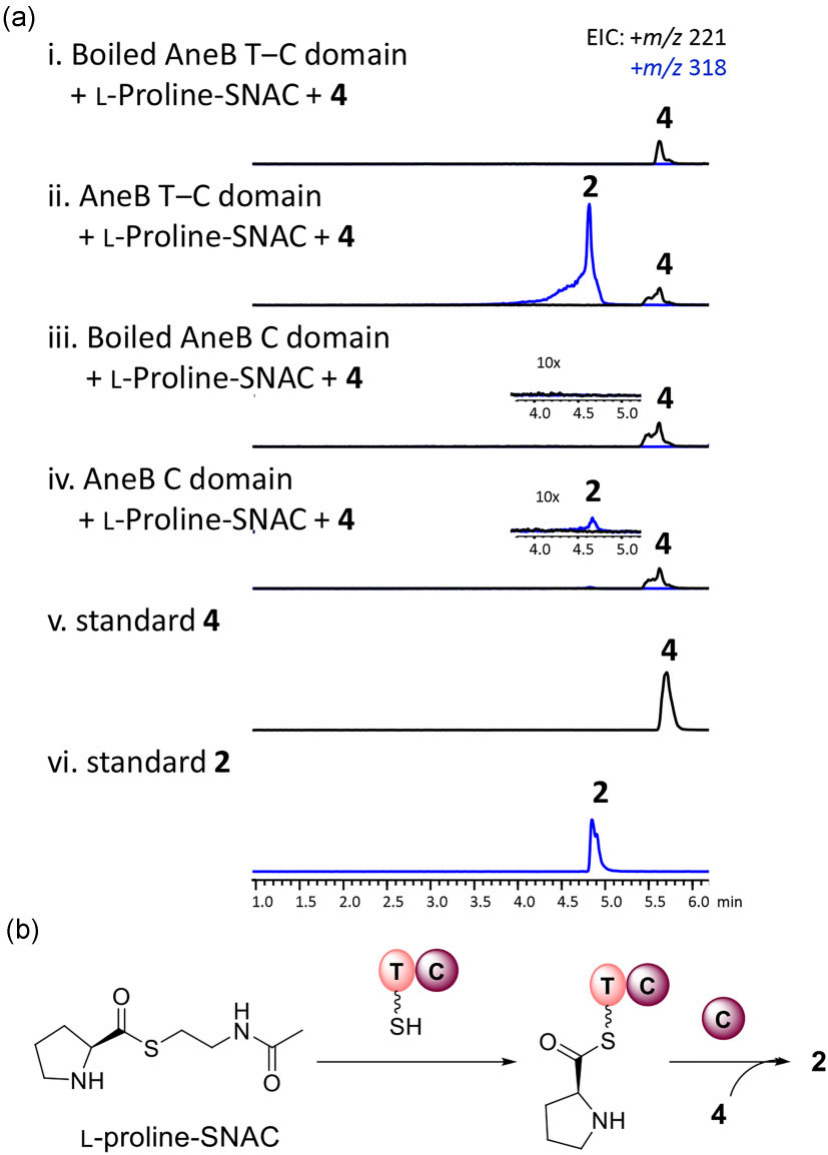
(a) Verification of function of the T‒C domain and C domain of AneB with L-proline-SNAC, and (b) the proposed mechanism.

Furthermore, to investigate the T domain dependence of C domain of AneB, we purified the recombinant T–C domain and the standalone C domain in the soluble form of *S. cerevisiae* BJ5464-NpgA and *E. coli* BL21 (DE3) (Supplementary Fig. S1), respectively, and analyzed the function of the dissected domains. A surrogate substrate, L-Pro-SNAC, was chemically synthesized for the experiments (Supplementary material). When L-Pro-SNAC was incubated with the T–C domain, we observed the production of **2** (Fig. 5). In contrast, incubating L-Pro-SNAC with the standalone C domain resulted in the formation of only trace amounts of **2** (Fig. 5). These findings demonstrate that a specific T-domain partner is required for the C-domain-catalyzed esterification reaction.

### Substrate Selectivity of AneB and ThmA

In our *in vivo* experiments using *S. cerevisiae* expressing *aneB*, we observed the emergence of compound **10**, both with and without the supplementation of **2** or **3** (Fig. 2b and 2c). These results suggest that AneB can also utilize endogenous yeast metabolites as substrates. Compound **10** was identified as proline phenethyl ester (Supplementary Figs. S13‒S17; Supplementary Table S5), a synthetic product previously reported and employed as a building block in the synthesis of FKBP12 inhibitors (Choi et al., 2002). Indeed, when we incubated 2-phenylethanol, L-proline, ATP, and MgCl_2_ with AneB, we observed the production of **10** (Fig. 2g). These results suggest the substrate promiscuity of AneB.

**Fig. 6.**
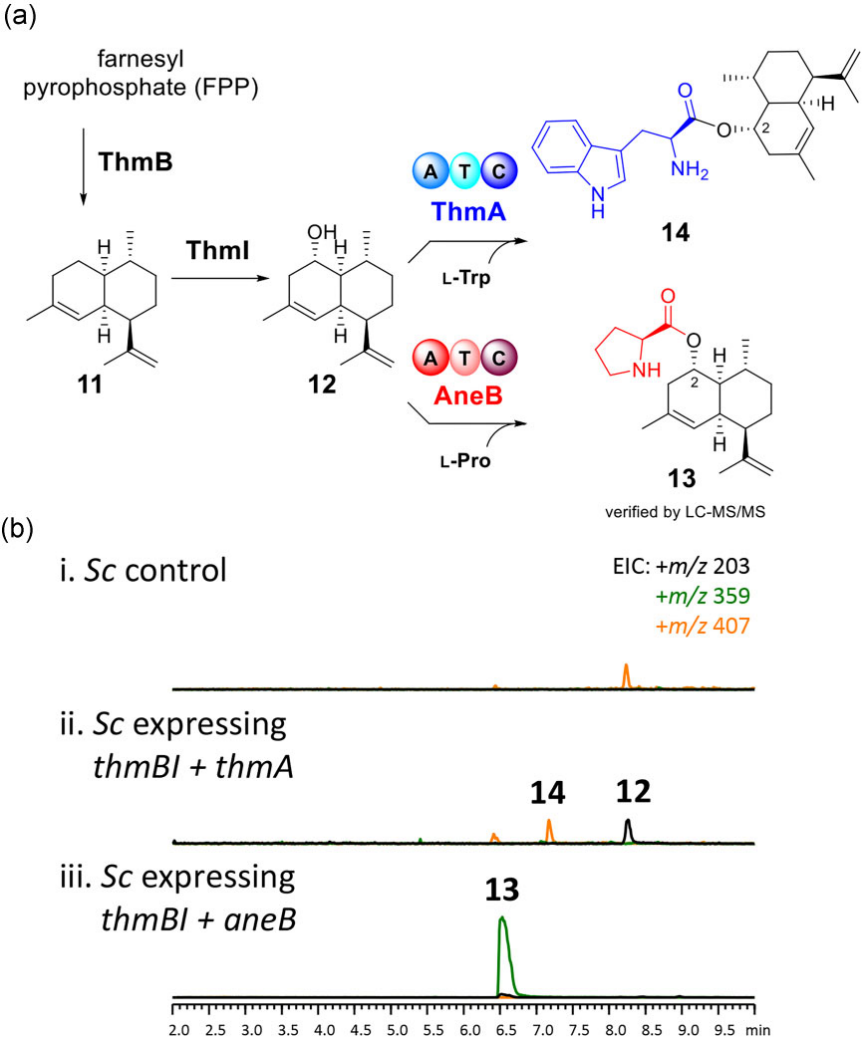
(a) Biosynthesis of amorpha-4,11-diene (**11**) and amorpha-4,11-diene-2-ol (**12**) and the divergent pathways catalyzed by ThmA and AneB with L-tryptophan and L-proline, respectively. (b) LC-MS analysis of *Saccharomyces cerevisiae* control and *S. cerevisiae* expressing *thmB/I* and *thmA* or *aneB*.

These above findings prompted us to investigate substrate selectivity of AneB. In the biosynthesis of CJ-12 662, ThmB terpene synthase in conjunction with P450 monooxygenase enzymes is responsible for generating a range of oxygenated amorpha-4,11-diene products (Cheng et al., 2022). To investigate whether AneB can accept other types of sesquiterpenes as substrates, we cloned synthetic DNA sequences of ThmB terpene synthase and ThmI P450 monoxygenase from the genome of azole-resistant *Aspergillus thermomutatus* strain HMR-AF-39 (*Neosartorya pseudofischeri*) (Parent-Michaud et al., 2019) into yeast gene expression vectors (Harvey et al., 2018; Hewage et al., 2023) and co-transformed into *S. cerevisiae* BJ5464-NpgA, which harbored *AtCPR* (Tang et al., 2015). As a result, the production of (‒)-amorpha-4,11-diene (**11**) and amorpha-4,11-diene-2-ol (**12**) was observed in *S. cerevisiae* strains expressing *thmB* and *thmB*/*I*, respectively (Supplementary Fig. S18‒S21; Supplementary Table S6‒S7). These results align with the findings obtained when using *Aspergillus nidulans* as a heterologous host for the reconstitution of the CJ-12 662 pathway (Cheng et al., 2022). To assess the catalytic property of AneB on amorpha-4,11-diene sesquiterpenes, we conducted co-expression experiments by introducing *aneB* with *thmB*/*I*. The production of **13** with an *m/z* value of 359 [M + H + CH_3_CN]^+^, corresponding to a prolyl-amorpha-4,11-diene-diol compound, was observed (Fig. 6). These results illustrate AneB's catalytic ability on mono-hydroxylated amorpha-4,11-diene substrates as a substrate for L-proline transfer.

**Fig. 7.**
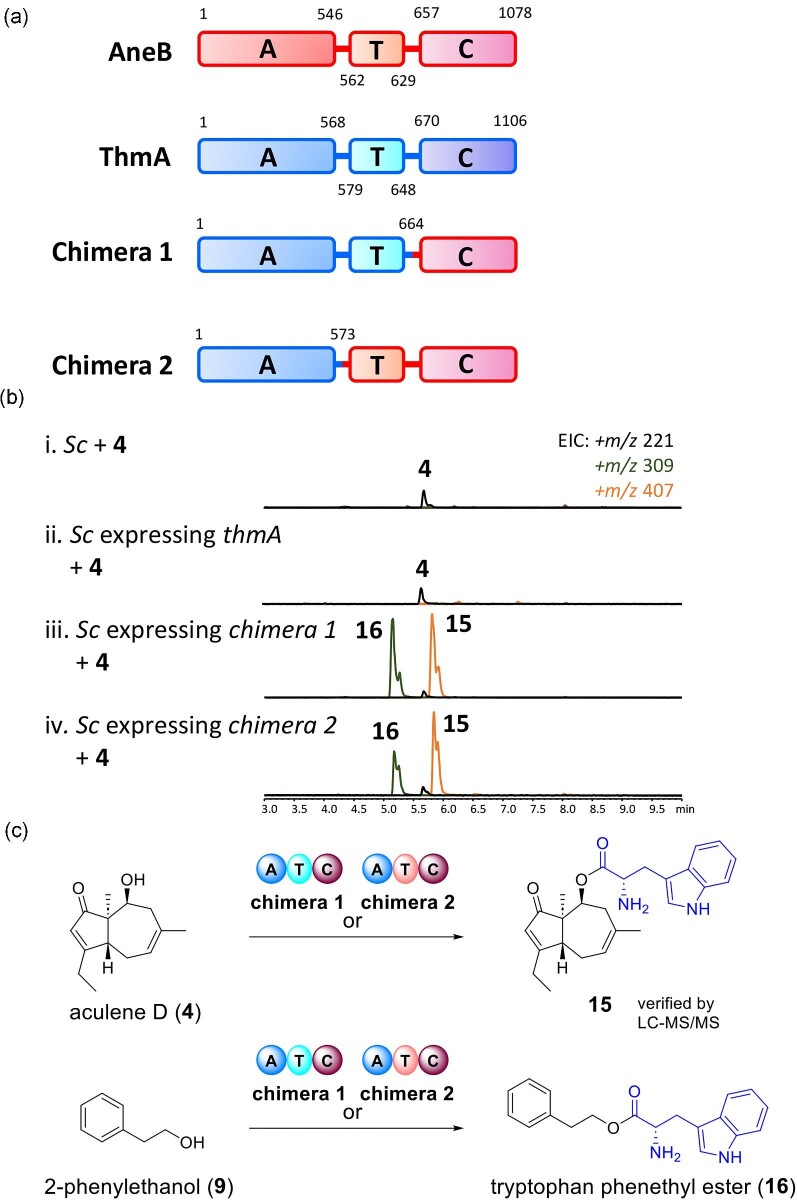
Verification of the function of ThmA and the chimeras 1 and 2 on **4** and **15**. (a) Schematic of the AneB and ThmA single-module nonribosomal peptide synthetases domains, and the chimera 1 (ThmA_1‒664_-AneB_647‒1078_) and chimera 2 (ThmA_1‒573_-AneB_552‒1078_) proteins. (b) LC-MS analysis of *Saccharomyces cerevisiae* expressing *thmA, chimeras 1* or *2* supplemented with **4**. (c) Conversion of **4** and **9** to **15** and **16**, respectively, with L-tryptophan by chimeras 1 or 2.

ThmA NRPS, which shares 34% amino acid sequence identity with AneB, has previously been reported to catalyze **12** for L-tryptophan installation (Cheng et al., 2022). Indeed, co-expression of *thmB*/*I*/*A* in *S. cerevisiae* leads to the production of tryptophanyl-amorpha-4,11-diene product (**14**) with *m/z* value of 407 [M + H]^+^ (Fig. 6). To determine if ThmA can utilize aculene sesquiterpene as a substrate, we supplemented **4** to *S. cerevisiae* expressing *thmA*. Instead, no product was observed, indicating that ThmA does not exhibit catalytic activity toward **4** (Fig. 7b).

In summary, these results demonstrate that AneB displays a broad substrate scope, including daucane and nordaucane sesquiterpenes, 2-phenylethanol, and amorpha-4,11-diene-2-ol, for the installation of L-proline. In contrast, ThmA exhibits high substrate selectivity, particularly select (‒)-amorpha-4,11-dienes for L-tryptophan transfer.

### Domain Swapping of AneB and ThmA Single-Module NRPSs

Our investigation into the domain functions and catalytic residues of AneB in this study has revealed that the C domain of AneB plays a crucial role in substrate recognition for the installation of L-proline. To explore the possibility of converting AneB into a transferase capable of installing L-tryptophan, we designed two chimeric enzymes. These chimeras consist of (i) the A and T domains of ThmA and the C domain of AneB (ThmA_1‒664_-AneB_647‒1078_) and (ii) the A domain of ThmA and the T and C domains of AneB (ThmA_1‒573_-AneB_552‒1078_), denoted as chimeras 1 and 2 (Fig. 6a). Remarkably, when we supplemented **4** to *S. cerevisiae* expressing chimeras 1 or 2, we observed the production of **15** with *m/z* 407 [M + H]^+^ and **16** with *m/z* 309 [M + H]^+^ (Fig. 6b). Compound **16** was purified and structurally characterized as tryptophan phenethyl ester (Supplementary Fig. S20–S22 and Supplementary Table S8). However, compound **15** was unstable during purification process and easily hydrolyzed into **4**. Therefore, the LC-MS/MS data of **15** were obtained to support its structure (Supplementary Fig. S25). These above results demonstrate several key characteristics of this single-module NRPS family: (i) the A domain determines the amino acid to be incorporated, like the A domains in other NRPSs from bacteria and fungi (Prieto et al., 2012); (ii) substrate selectivity, such as the types of sesquiterpenes used, is controlled by the C domain; (iii) both the T domains of AneB and ThmA can facilitate L-tryptophan transfer when incorporated into these chimeric enzymes, and they are indispensable for the C domain-catalyzed esterification reaction (as confirmed by the described *in vitro* experiments involving the T–C domain of AneB).

## Discussion

Single-module NRPS and NRPS-like enzymes have garnered increased interest as biocatalysts due to their functional diversity. These enzymes catalyze carboxylic acid substrates, select and activate them via the A domain, and tether them as thioesters onto the 4’-phosphopantetheine arm of the T domain. Depending on the function of downstream releasing domains, the thioester intermediates attached to the T domain undergo a wide range of modifications, resulting in the generation of natural product scaffolds with structural diversity. For example, the C domain within A‒T‒C catalyzes amidation (Gao et al., 2011) or *N*-cinnamoylation (Li et al., 2021), while the thioesterase (TE) domain in A‒T‒TE facilitates Dieckman/aldol condensation, converting two tethered indole-3-pyruvate molecules into *bis*-indolylquinones (Balibar et al., 2007; Schneider et al., 2007) or dimerizing activated α-keto carboxylic acids to yield hydroxybenzoquinones/lactones (Hühner et al., 2019; van Dijk et al., 2016). The reductase (R) domain in A-T-R catalyzes reduction, leading to the formation of the dibenzylpyrazine/dibenzylpiperazine scaffold from aromatic amino acids (Li et al., 2021; Pham et al., 2022; Yu et al., 2016), as observed in A‒T‒R‒R, which reduces tethered glycine betaine to choline (Hai, Huang, et al., 2019). The A‒T‒R‒P domain carries out reduction and pyridoxal phosphate (PLP)-dependent aldol condensation, playing a crucial role in the generation of isoquinoline (Baccile et al., 2016). Lastly, the epimerization (E) domain in A‒T‒E‒C unidirectionally catalyzes stereoinversion, converting activated L-tryptophan to D-tryptophan (Hai, Jenner, et al., 2019). On the other hand, a single-module NRPS, DrcB, featuring the A‒T‒C_T_ domain, was discovered in the biosynthesis of polyketide-amino acid hybrids. DrcB catalyzes the formation of an amide bond between depside and L-threonine, yielding a depside−threonine hybrid, duricamidepside (Chen et al., 2022). These findings shed light on the catalytic versatility of the class of single-module NRPS in synthesizing a diverse range of hybrid molecules.

In the biosynthesis of terpene-amino acid natural products, we have demonstrated that the C domain within A‒T‒C of AneB and ThmA serves as the selector of sesquiterpene substrates and catalyzes ester bond formation with amino acids, selected and activated by the A domain. Domain swapping experiments have indicated that the A‒T domains of ThmA can be effectively utilized by the C domain of AneB, suggesting the possibilities of incorporating various amino acids through A domain selectivity. The substrate promiscuity observed in the C domain of AneB requires further examination to unveil the potential for incorporating various sesquiterpenes, thereby generating terpene-amino acid meroterpenoid derivatives. Obtaining protein structural information regarding the C domains of AneB and ThmA will offer insights into sesquiterpene substrate recognition and provide valuable ideas for expanding substrate scope through protein design.

In conclusion, our study elucidates the catalytic properties of AneB, showing its ability to catalyze esterification with L-proline on a range of substrates, including daucane/nordaucane sesquiterpenes, oxygenated amorpha-4,11-diene, and 2-phenylethanol. Furthermore, we demonstrated that the choice of amino acid can be interchanged with L-tryptophan by swapping the A domain of ThmA NRPS, enabling the generation of tryptophanyl derivatives. Additionally, we verified the esterification function of the C and T‒C domains of AneB on L-proline-SNAC through *in vitro* experiments, demonstrating the indispensable role of the T domain for L-proline transfer using surrogate substrate, and confirming the catalytic residues of the C domain. This work highlights the potential for utilizing AneB in domain engineering to synthesize terpene-amino acid meroterpenoid derivatives.

## Supplementary Material

kuad043_Supplemental_File
