# Correction to: Characterization and catalytic investigation of fungal single-module nonribosomal peptide synthetase in terpene-amino acid meroterpenoid biosynthesis

**DOI:** 10.1093/jimb/kuae002

**Published:** 2024-01-17

This is a correction to: Cheng-Chung Tseng, Li‐Xun Chen, Chi‐Fang Lee, Zhijay Tu, Chun-Hung Lin, Hsiao-Ching Lin, Characterization and catalytic investigation of fungal single-module nonribosomal peptide synthetase in terpene-amino acid meroterpenoid biosynthesis, *Journal of Industrial Microbiology and Biotechnology*, Volume 50, Issue 1, 2023, kuad043, https://doi.org/10.1093/jimb/kuad043

In the originally published version of this manuscript, the graphical abstract required correction.

The required change was on the stereochemistry of tryptophan of the two compounds shown on the left, which should have been drawn as L-form, not as D-form.

The graphical abstract has been updated to the correct version.

