# Supplementary material for: Characterization and catalytic investigation of fungal single-module nonribosomal peptide synthetase in terpene-amino acid meroterpenoid biosynthesis
Source: J Ind Microbiol Biotechnol. 2023 Dec 4;50(1):kuad043. doi: 10.1093/jimb/kuad043 (PMC10720950; doi:10.1093/jimb/kuad043)
Supplement: kuad043_Supplemental_File [file kuad043_supplemental_file.pdf]

Supplementary Material

Characterization and Catalytic Investigation of Fungal Single-Module Nonribosomal Peptide Synthetase in Terpene-Amino Acid Meroterpenoid Biosynthesis

Cheng-Chung Tseng[1,2], Li-Xun Chen[1,3], Chi-Fang Lee[1,3], Zhijay Tu[1], Chun-Hung Lin[1,3], Hsiao-Ching Lin*[1,2,3]

[1] Institute of Biological Chemistry, Academia Sinica, Taipei 115, Taiwan R.O.C.
[2] School of Pharmacy, College of Medicine, National Taiwan University, Taipei 100, Taiwan R.O.C.
[3] Institute of Biochemical Sciences, National Taiwan University, Taipei 106, Taiwan R.O.C.

Table of Contents

Supplementary Material........1

Table of Contents........2

List of Tables........3

List of Figures........3

List of Scheme........4

1 Experimental Procedures........5

1.1 General molecular biology experiments........5

1.2 Plasmid cloning and construction........5

1.2.1 Construction of plasmids pXW55-AneB (NRPS) and pXW06-AneE (hydrolase) for expression in S. cerevisiae........5

1.2.2 Construction of plasmids pXW55H-AneB-S596A, pXW55H-AneB-Q793A, pXW55H-AneB-Q793H, pXW55H-AneB-H794A, pXW55H-AneB-L796A, pXW55H-AneB-Y979A, pXW55H-AneB-D798A and pXW55H-AneB-S801A for expression in S. cerevisiae........5

1.2.3 Construction of plasmids pXW55-AneB TC domain and pColdI-MBP-AneB C domain........6

1.2.4 Construction of plasmids pXW06H-ThmA, ThmA (AT domain)-AneB (C domain) and ThmA (A domain)-AneB (TC domain)........6

1.2.5 Construction of plasmids pXW55H-ThmB (TPS) and pXW06H-ThmI (P450)6

1.2.6 Construction of plasmids pXW55H-ThmB (TPS)-AneB(NRPS)........7

1.3 Chemical synthesis of L-Proline-SNAC........7

2 Supplementary Tables........8

3 Supplementary Figures........18

References........48

List of Tables

Table S1. Primer list 8
Table S2. Synthesized DNA sequences in this study. 10
Table S3. *S. cerevisiae* transformants in this study. 12
Table S4. ^{1}H NMR (500 MHz), ^{13}C NMR (125 MHz) and 2D spectroscopic data of asperaculane H (**6**) in DMSO-*d6*. 13
Table S5. ^{1}H NMR (500 MHz), ^{13}C NMR (125 MHz) and 2D spectroscopic data of proline phenethyl ester (**10**) in CD3OD. 14
Table S6. Comparison of ^{1}H NMR (500 MHz) and ^{13}C NMR (125 MHz) spectroscopic data of amorpha-4,11-diene (**11**) to reported data.[1] 15
Table S7. Comparison of ^{1}H NMR (500 MHz) and ^{13}C NMR (125 MHz) spectroscopic data of amorpha-4,11-diene-2-ol (**12**) to the reported data.[2] 16
Table S8. Comparison of ^{1}H NMR (500 MHz) and ^{13}C NMR (125 MHz) spectroscopic data of tryptophan phenethyl ester (**16**) to the reported data.[3] 17

List of Figures

Fig. S1. SDS-PAGE of purified proteins. (A) AneB (120.5 kDa), (B) AneB TC domain (59.8 kDa), (C) AneB C domain (48.9 kDa). 18
Fig. S2. Sequence alignment of single-modular NRPSs AneB, ThmA and FlvI. 19
Fig. S3. Predicted structures by AlphaFold and simulated substrate binding of AneB and ThmA. 20
Fig. S4. LC-MS analysis of *in vitro* assays of AneB and (A) asperaculane A with L-proline, and (B) **4** with L-tryptophan, L-leucine, L-histidine, L-asparagine and L-aspartate, respectively 21
Fig. S5. HPLC profiles of (A) *S. cerevisiae* expressing *chimera 1* supplemented with **4**, analyzed by (A) analytical column and (B) semi-preparative column. 22
Fig. S6. Sequence alignment of the predicted linker regions between A and T domains, and T and C domain of AneB, ThmA and FlvI. Based on the alignments, chimera 1 and 2 designed in this study are $ThmA_{1-664}$-$AneB_{647-1078}$ and $ThmA_{1-573}$-$AneB_{552-1078}$. 23
Fig. S7. ^{1}H-NMR spectrum (DMSO-d_6, 600 MHz) of **6**. 24
Fig. S8. ^{13}C-NMR and DEPT135 spectra (DMSO-*d6*, 125 MHz) of **6**. 25
Fig. S9. HSQC spectrum (DMSO-d_6, 500 MHz) of **6**. 26
Fig. S10. HMBC spectrum (DMSO-d_6, 500 MHz) of **6**. 27
Fig. S11. COSY spectrum (DMSO-d_6, 500 MHz) of **6**. 28

Fig. S12. NOESY spectrum (DMSO-d_6, 500 MHz) of **6**. 29
Fig. S13. ^{1}H-NMR spectrum (CD_3OD, 500 MHz) of **10**...30
Fig. S14. ^{13}C-NMR, DEPT135 and DEPT90 spectra (CD_3OD, 125 MHz) of **10**.......31
Fig. S15. HSQC spectrum (CD_3OD, 500 MHz) of **10**...32
Fig. S16. HMBC spectrum (CD_3OD, 500 MHz) of **10**. 33
Fig. S17. COSY spectrum (CD_3OD, 500 MHz) of **10**...34
Fig. S18. ^{1}H-NMR spectrum ($CDCl_3$, 500 MHz) of **11**...35
Fig. S19. ^{13}C-NMR, DEPT135 and DEPT90 spectra ($CDCl_3$, 125 MHz) of **11**.........36
Fig. S20. ^{1}H-NMR spectrum ($CDCl_3$, 500 MHz) of **12**...37
Fig. S21.^{13}C-NMR, DEPT135 and DEPT90 spectra ($CDCl_3$, 125 MHz) of **12**..........38
Fig. S22. ^{1}H-NMR spectrum (DMSO-d_6, 500 MHz) of **16**...39
Fig. S23.^{13}C-NMR spectrum (DMSO-d_6, 125 MHz) of **16**...40
Fig. S24. MS/MS spectra of **10** and **16**...41
Fig. S25. MS/MS spectra of **2** and **15**...42
Fig. S26. MS/MS spectra of **12** and **13**...43
Fig. S27. HRMS spectra of asperaculane H (**6**)..44
Fig. S28. UV and MS spectra of **1−5**. MS spectra are at positive mode. 45
Fig. S29. UV and MS spectra of **6−10**. MS spectra are at positive mode. 46
Fig. S30. UV and MS spectra of **12−16**. MS spectra are at positive mode. 47

List of Scheme

Scheme S1. Synthetic route to the L-Proline-SNAC HCl salt......................................7

1 Experimental Procedures

1.1 General molecular biology experiments

Polymerase chain reaction (PCR) was carried out using Q5® High Fidelity DNA polymerase sets (New England Biolabs). PCR products were purified using the Zymoclean™ Gel DNA Recovery Kit (ZYMO RESEARCH), and DNA fragments were assembled using the NEBuilder® HiFi DNA Assembly Master Mix (New England Biolabs) or ligated with the Zero BluntTM TOPOTM PCR Cloning Kit (Invitrogen). The assembled or ligated DNA constructs were transformed into *E. coli* DH10β (Invitrogen) strain for cloning. Restriction enzymes for digestion verification were used as recommended by the manufacturer (New England Biolabs). RNA was extracted using the RNeasy® Plant Mini Kit (QIAGEN) and the RiboPure® Yeast Kit (Ambion). Reverse transcription PCR (RT-PCR) was performed with the GoScript™ Reverse Transcription System (Promega) to obtain complementary DNA (cDNA) from total RNA.

1.2 Plasmid cloning and construction

1.2.1 Construction of plasmids pXW55-AneB (NRPS) and pXW06-AneE (hydrolase) for expression in *S. cerevisiae*

The intron-free *aneB* and *aneE* genes were amplified using the primer pairs AneB-XW55-F/R and AneE-XW55-F/R, respectively. To construct pXW55-AneB and pXW06-AneE, the intron-free *aneB* and *aneE* DNA fragments were ligated into the linearized YEplac195-derived 2μ expression plasmid, which was linearized with NdeI/PmlI (containing URA3 marker) for pXW55-AneB and NdeI/SpeI (containing TRP1 marker) for pXW06-AneE.

1.2.2 Construction of plasmids pXW55H-AneB-S596A, pXW55H-AneB-Q793A, pXW55H-AneB-Q793H, pXW55H-AneB-H794A, pXW55H-AneB-L796A, pXW55H-AneB-Y979A, pXW55H-AneB-D798A and pXW55H-AneB-S801A for expression in *S. cerevisiae*

To construct mutant plasmids, DNA fragments were generated by amplification from pXW55H-AneB using the primers listed in Table S1. Specifically, pXW55H-AneB-**S596**A, pXW55H-AneB-Q793A, pXW55H-AneB-H794A, pXW55H-AneB-L7**96**A, pXW55H-AneB-Y797A, pXW55H-AneB-D798A, pXW55H-AneB-Y**797**A, and pXW55H-AneB-D7**98**A were amplified using the primer pair, AneB-S5**96**A/KP337-P2, AneB-Q793A/AneB-NRPS-CM-R, AneB-H794A/AneB NRPS-CM-R2, L796A/AneB-NRPS-CM-R, AneB-C-Y797A/AneB NRPS-CM-R, and AneB-H794A/AneB NRPS-CM-R2, respectively. For constructing the mutant plasmids AneB-Q793H and AneB-S801A, overlapping regions were amplified and assembled

using the following primer pairs: AneB-Q793H-F/URA3-R, AneB-Q793H-R/URA3-F, AneB-S801A-F/URA3-R, and AneB-S801A-R/URA3-F.

1.2.3 Construction of plasmids pXW55-AneB TC domain and pColdI-MBP-AneB C domain

Truncated AneB plasmids were generated by amplifying from pXW55H-AneB using the primers listed in Table S1. Specifically, the pXW55-AneB TC domain was amplified from the AneB sequence containing amino acids 554-1078 using the primer pair AneB-Td-XW55-F/AneB-XW55-R and assembled into the NdeI/PmlI linearized YEplac195-derived 2μ expression plasmid. Furthermore, the pColdI-MBP-Ane C domain was amplified from the AneB sequence covering amino acids 641-1078. This amplification was achieved using the primer pairs pColdI-6xHis-MBP-F/TEV-MBP-R and TEV-AneB Cd-F/pColdI-AneB-R. Subsequently, the assembled fragment was ligated into the pColdTM I vector, which had been linearized through NdeI/Sal1 restriction enzyme digestion. This construct was designed for the expression of N-6xHis-MBP tagged recombinant proteins in *E. coli*.

1.2.4 Construction of plasmids pXW06H-ThmA, ThmA (AT domain)-AneB (C domain) and ThmA (A domain)-AneB (TC domain)

To construct pXW06H-ThmA, the intron-free *thmA* (NRPS) sequences were synthesized by Integrated DNA Technologies IDT (Table S2). Additionally, pXW06H-ThmA (AT domain)-AneB (C domain) was generated by PCR amplification. The ThmA sequence spanning amino acids 1-664 was amplified using the primer pair ThmA-XW06-F/ThmA-664-R, and the AneB sequence from amino acids 647-1078 was amplified with the primer pair AneB-647-F/AneB-XW06-R. Similarly, pXW06H-ThmA (A domain)-AneB (TC domain) was constructed by amplifying the ThmA sequence from amino acids 1-573 using the primer pair ThmA-XW06-F/ThmA-573-R. The AneB sequence containing amino acids 552-1078 was amplified with the primer pair AneB-552-F/AneB-XW06-R. The DNA fragments mentioned above were assembled into the NdeI/PmlI-linearized YEplac195-derived 2μ expression plasmid.

1.2.5 Construction of plasmids pXW55H-ThmB (TPS) and pXW06H-ThmI (P450)

To construct pXW55H-ThmB (TPS) and pXW06H-ThmI (P450), intron-free sequences for thmB and thmI were synthesized by Integrated DNA Technologies IDT (Table S2). These DNA fragments were then assembled into the NdeI/PmlI-linearized YEplac195-derived 2μ expression plasmid and the NdeI/SpeI-linearized plasmid, respectively.

1.2.6 Construction of plasmids pXW55H-ThmB (TPS)-AneB(NRPS)

To construct pXW55-ThmB-AneB, linearized DNA fragments were assembled, which were amplified using the primer pairs Vector-F/R from pXW55H-ThmB (TPS) and Insert-F/R from pXW55H-AneB (NRPS).

1.3 Chemical synthesis of L-Proline-SNAC

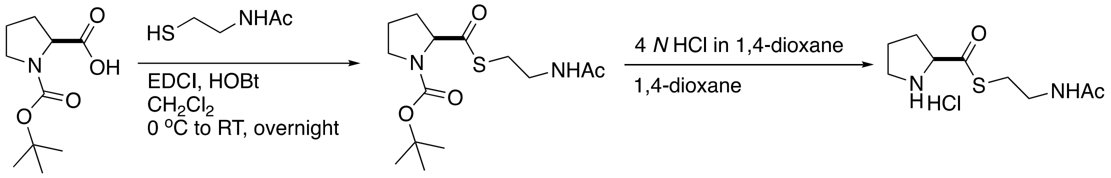

Scheme S1. Synthetic route to the L-Proline-SNAC HCl salt.

To the solution of commercially available Boc-L-Proline (300 mg, 1.39 mmol) and 1-hydroxybenzotriazole (HOBt) (188 mg, 1.39 mmol) in dry CH_2Cl_2 (10 mL) was sequentially added the 1-ethyl-3-(3-dimethylaminopropyl)carbodiimide HCl salt (EDCI-HCl) (400 mg, 2.09 mmol) and *N*-acetylcysteamine (SNAC) (182 mg, 1.53 mmol) with stirring at 0 °C under nitrogen atmosphere. The resulting mixture was allowed to gradually warm to room temperature with stirring overnight, which was evaporated to a dryness residue. The residue was re-dissolved in EtOAc (10 mL) and washed with water and brine for several times. The combined organic layers were dried over $MgSO_4$, filtered and concentrated *in vacuo* to provide the crude. The crude was chromatographically purified on Büchi Pure C-850 FlashPrep automated purification machine monitored by ELSD/UV scan detector to afford the desired thiol-ester, Boc-L-Proline-SNAC (110 mg, 25 %) as a colorless syrup.

^{1}H NMR (500 MHz, $CDCl_3$): rotamers δ 6.22 (br, 1H, NH), 6.03 (br, 1H, NH), 4.44 (dd, *J*= 8.7, 2.9 Hz, 1H, CH), 4.37 (dd, *J*= 8.7, 3.4 Hz, 1H, CH), 3.59-3.33 (m, 8H, CH_2x4), 3.10-2.97 (m, 4H, CH_2x2), 2.28-2.15 (m, 2H, CH_2x1), 2.03-1.88 (m, 12H, CH_2x3+CH_3x2), 1.48 (s, 9H, ^{t}Bu), 1.42 (s, 9H, ^{t}Bu). ^{13}C NMR (125 MHz, $CDCl_3$): rotamers δ 203.4 (C=O), 202.7 (C=O), 170.8 (C=O), 170.5 (C=O), 154.9 (C=O), 154.0 (C=O), 80.7 (C), 80.6 (C), 66.4 (CH), 66.2 (CH), 47.1 (CH_2), 46.8 (CH_2), 39.9 (CH_2), 39.4 (CH_2), 31.8 (CH_2), 30.8 (CH_2), 28.6 (CH_3), 28.5 (CH_3), 28.3 (CH_2), 28.1 (CH_2), 24.4 (CH_2), 23.6 (CH_2), 23.3 (CH_3), 23.2 (CH_3). HRMS (ESI): *m/z* calcd for $C_{14}H_{24}N_2O_4S$ $[M+H]^+$: 317.1530; found: 317.1533.

2 Supplementary Tables

Table S1. Primer list

Primer name	Sequence (5' to 3')
pXW55H-AneB	
AneB-XW55-F	AACTATCAACTATTAACTATATCGTAATACCATATGACTCTGGACGACAATCCTGTC
AneB-XW55-R	GATGGTGATGCACGTGCGCCCCCAGGTGCTG
pXW55-AneE	
AneE-XW06-F	TTAACTATATCGTAATACCATCATATGACCAAAGATAACGAAGAGAAAAC
AneE-XW06-R	TGGTGGTGGTGGTGACTAGTCTGGCTTCCGCTCTTCAGCG
pXW55-AneB-S596A	
AneB-S596A	AACCGCATCGCTGCGAGGGCCTCGCCCCCGAGCGA
KO337-P2	GCAGAGCTATCAGTCGCAGCTCC
pXW55-AneB-Q793A	
AneB-Q793A	GATGTTGATCGCGCATGCGCTC
AneB NRPS-CM-R	CGAACCCGGCGAATTTCTCTCG
pXW55-AneB-Q793H	
AneB-Q793H-F	GGCTGGTGATGTTGATCCATCATGCGCTCTACGATGG
AneB-Q793H-R	CCATCGTAGAGCGCATGATGGATCAACATCACCAGCC
pXW55-AneB-H794A	
AneB-H794A	GGCTGGTGATGTTGATCCAGGCTGCGCTCTACGATGGTCACTC
AneB NRPS-CM-R2	GATGTCACAGGTGTCGAAATGCCAGGAGAG
pXW55-AneB-L796A	
AneB-L796A	CAGCATGCGGCCTACGATG
AneB NRPS-CM-R	CGAACCCGGCGAATTTCTCTCG
pXW55-AneB-Y797A	
AneB-C-Y797A	GCATGCGCTCGCCGATGGTC
AneB NRPS-CM-R	CGAACCCGGCGAATTTCTCTCG
pXW55-AneB-D798A	
AneB-C-D798A	CATGCGCTCTACGCTGGTCAC
AneB NRPS-CM-R	CGAACCCGGCGAATTTCTCTCG
pXW55-AneB-S801A	
AneB-S801A-F	GCGCTCTACGATGGTCACGCGCTCGGGCTCATCTTCC
AneB-S801A-R	CGCGTGACCATCGTAGAGCGC
pXW55-AneB T+C domain	
AneB-Td-XW55-F	ATCAACTATTAACTATATCGTAATACCATATGAGGAGCACGGAGGGGATG
AneB-XW55-R	GATGGTGATGCACGTGCGCCCCCAGGTGCTG
pColdI-MBP-AneB C domain	

pColdI-6xHis-MBP-F	CACAAAGTGCATCATCATCATCATCATATGAAAATCGAAGAAGGTAAACTGGTAATCTGG
TEV-MBP-R	GGATTGGAAGTACAGGTTCTCCCCGAGGTTGTTGTTATTGTTATTGTTGTTG
TEV-AneB Cd-F	GAGAACCTGTACTTCCAATCCCATATGGAGAGCTTTCCGCCATTCTCG
pColdI-AneB-R	CAGAGATTACCTATCTAGACTGCAGGTCGACCTACGCCCCCAGGTGCTGGCG
pXW06H-ThmA(AT domain)-AneB(C domain)	
ThmA-XW06-F	AACTATCAACTATTAACTATATCGTAATACCATATGCCTGGAGTTGAAGCAATCG
ThmA-664-R	AAATGGCCCTGGGTTCGTAACC
AneB-647-F	GGTTACGAACCCAGGGCCATTTTCGTTGGTGCAGTGCCAGCAGG
AneB-XW06-R	TCAGTGGTGGTGGTGGTGGTGACTAGTCGCCCCCAGGTGCTGG
pXW06H-ThmA(A domain)-AneB(TC domain)	
ThmA-XW06-F	AACTATCAACTATTAACTATATCGTAATACCATATGCCTGGAGTTGAAGCAATCG
ThmA-573-R	CCCGTCTTCGTCCCCGCG
AneB-552-F	GCTTATCGCGGGGACGAAGACGGGGGTAGCAGGAGCACGGAGG
AneB-XW06-R	TCAGTGGTGGTGGTGGTGGTGACTAGTCGCCCCCAGGTGCTGG
other	
Insert-F	GGCGTAATCATGGTCATAGCTGTGCGGCCGCAAAACGTAGG
Insert-R	GCGTTGGCCGATTCATTAATGCACACGACGTTGTAAAACGACGG
Vector-F	CCTACGTTTTGCGGCCGCACAGCTATGACCATGATTACGCC
Vector-R	CCGTCGTTTTACAACGTCGTGTGCATTAATGAATCGGCCAACGC
URA3-F	CTAGGTTCCTTTGTTACTTCTTCTGCCG
URA3-R	CGGCAGAAGAAGTAACAAAGGAACCTAG

Table S2. Synthesized DNA sequences in this study.

Gene name	Sequence (5’ to 3’)
thmB	atgcgttacatcaatcattttaactgtcccgtagactgggcggtctccgtcaggaccagtcatgacaagttcgggttccttgatccgaatcgtccggaga<br>agtcaaacagctgtcccaattttgagatcctatgccctgccgcgttgggggttgactggaggacctccctgaggggttcgaagcagaatagatatgac<br>aagatggccgaagaagcggcatacgagctatggcatgagattcggcgggcggatatcgaccagcagggggccttgcccaaaaccatcaagaaga<br>gggataaacagttcgaggtgatgaatacggctattgactgtgctattaatctctattcatcctgtgatccccagaagataagagtggtcactaaagcgatg<br>atgctcttgttcttacacgacgatgtcattgaatacgcctattccaaggatagcgatacgatcctagagcaagaaataggcatccacggcacagccgag<br>gagacattgcgtaccgaccctgacaagggggccatctgggcgagatttgtacgacagacgctggagattgatcctgtctggggccctggcctcctgc<br>gaggcatgattgcctggagtgcatttacgaatgaacatcaacattcgctccggagttccttctctaccctgtccgagtacctacagtttcgagagatggat<br>gtcggcaatgaggtgatcatcgcgtgcctccgatacagctgcgaattccaactgacgccaagcgacgtcgacgccgtgcacgacctggagacgctg<br>atggtcagacattgtcttctaacaaatgacctgttttccttcaataaggaacagtgtgagcgtgtctcaacgggagcgtcaattgtcaataccgtcgagtg<br>cctgcgaactttaatgacgacctctgtgagtgctgccaaatccatggcgctgacccttctctgggaggttgagcagaagatgcatgaagtctacgagc<br>gagggttgctagaatggaccgacttacaggctctttatgcccaacgcgtgatcgagttatccgcaggtaactatttcttctctgcaactacctaccgctac<br>tccaccgaagccatcaaagtgcatcttgactttgcctctaaggttggcgcatctttg
thmI	atggatctctttgcgtttctccttgtgagtttcgcgataacagttctgcgcctgttctatgagcattaccgtgatcgaaaactgcctccagggcctccccga<br>cttccattcgtcggcaaccttctccagatgcctttgaagcgccagtggttgaaattcgacgaatggagtcgcaaatacggtcccatcttctccctcaacct<br>ggctgggtctgtgtatgtcgttatttccgatcctcagattgcgtgcagagtcctggaaaagcgctcggccagcgaccggccgcgcatggtaatgtttca<br>agatcatgtgggaaaggggatgtacaatgcctacggaatgtaccatgagggctaccgacttcatcagcgcatgcaaggccctgtgatgagccgtcgg<br>gccgcgcagacatattcttccgtgcaggacctcgagacaaagcacaccctgtatgaaatgttgtcctccaacgactataagaagctgtatacgcggttc<br>aacgccagcgtcatcttttcgctagtctatgggaagcgcattatcacaggcgaggaaactgaactgcaggagtggctgcagagacatcacaatttcac<br>catggtgaccgcctccccgtggctggtggactttttccccgtcttggaccacattgttcccagattcctcgctccttggaagaagatcggagacgagata<br>gcctcgtttgaatcccgcgtattactcaagcacatgaagatgggtctccaaagttcctcctggaactgggccaaggaatttaccgcctcccctgaaggt<br>cggcaagtcagtccactccagatcgcctacgatctaggtctattaatcgatgccgggctcgaaaccacctctaccatcatggaagtattcaccctggcg<br>gcgatcgttgcccccgaagccatgcgcaaagcccaggaagaactggatcgtgtcgttggccctgaccggctgccatcgctcggggactgtgagag<br>attaccctacatgtcagccgtggtcgacgaaacccaacgttggcgcctcatcagtcccctcatgttcccgcatggaatgatggaagaagacacttgctt<br>gggatatacaatccccaagggagcaaccgttttcgccctcgggtataccatgtcactagatgagagcaggttcgacaaacccctggagtttcgcccgg<br>agagatggctggagaaggttgagaacggtgggttcaaaagtttctttggatacgggcgccggggttgccctggtagagacattgggaaaaactcatta<br>ttcttaatgttctcgcgcatcctatgggcctacaatgttaaaaatggcgactccaaggctcctgatgaccgagcctcttcgtcggcgttgatctccgttccg<br>cagcaattcaaggtcatcttcgagcctcggagcactcatcaccgtatgatgatcgaaagggaatggatcaatgccgagaaggatatcgacttgttgatg<br>cgggaaatcaaggagcgtcagatgaagctaggaatggatcgtggaggg
thmA	atgcctggagttgaagcaatcgcacttccagagggctggcagagatccacccccttctgatcaacaaatgtcaaactggaataagacgcccattgcatc<br>ggttaatctgtgtatacatgaactcatcctccaacgctgccagcagcatccggagaaaatcgcggtctctgcttgggatggggagttcacctacgaaca<br>gctcgaccgccactctgctgcgttggcccacctccttcatgagtacgcgattgggcctgagacattcattcctatctgctttgagaagtctcgctggacg<br>gtggtggccattctggggatcctccgagcgggagcggcttttgtgcttctcgacccatcctacccagttcagcgcctcggagagatcttccgtgaggtt<br>gctgccccattgcttgttgctactatttcctgtgccaaggcagcatctgagctatcctcgaaggtgatcatcattgacggcgacagttggataccatcagg<br>tgaaggaatagagccgccattgggccaccgatctcccgtcaagcctagcaatgccgcctatgctgtcttcacctctggaacgactggcaagccgaag<br>ggggtggtaattgagcatgctgcgtttgccaccatggccatcgcgcagattgagctaattcctttcacgacacaaacccgcctcctgcagttttcctcgt<br>acgccttcgacgtcagtatctctgaccagctcttgagcctgatggccggggcgtgtatttgcgtgccgtccgagatagatcgccaggaaaacctacgg<br>ggttttatcaaccagcatcaggtgaattgtgcttttgtcacttcctccgtggctcggctactttcaccagacgcggttccttcactgcagctgctgggcctg<br>gggggagagaaagtggcccggtccgacattgaaacctggtcgccgtatgttcggctgctgggtgagtacggcccggccgaatgttcggttacttgcg<br>cggttaatctcgatttagcaaggtcaattgacgcgcccggtaatattggattccctttggcagcgagattctgggttatagatccagaaaatgcggaggtt<br>ctgatgcctgtagggtcacccggggagttggccattgaagggccgacactggcccgtgggtatctcaatgatcctgacagaacggctgccgcgttcg<br>tcagcccagattggttgtcgtccctacgacgaggagagaggtctcgcgtttatcggaccggcgatttagttcagtacgagcgcgacggctcgcttatct<br>ttctgggccgcaaagacgcacaggtcaaaattaacggtcagcgggttgagctggaggatatcgagcatcacctgcgaaagtgcttcccaggtgctct<br>ggatatcatcgctgaggtcgtcttctttggtgctggggagtcttctaggctggttgcattcgtcaaggagaccatagaaggcgaggagaatcaatctcat<br>atctctgacaacaccctccttcgacccccgacaaagaacttccagaatcgcattgtagagtccgaagcagctctgctacgacagcttccccggtacatg<br>gttcccagcctcttcttccctgtggatcatatccccatcacgacatcgggaaaggcaaaccgccgggcattgcaggaaatggtgatgcagctcccctc<br>agagcgattagccgcttatcgcggggacgaagacgggttcagcccacccagaaacgtaatggaagagacgctgcgcagactgtggacaaagacc

	cttcgaacttcgttttccactgtcgggcgccactccaactggtggcgcctagggggcgactccttgcgggccatccggttggtcggagagctacagat<br>ggaagggctctttatcactgtgcgggagatcttccagcatccagtgcttgcggacatggcggcggtgatcaagagggagaccgcatcctccagtgcg<br>gttacgaacccagggccatttgaacttctgggtaaggacgaaacgactcggatgagacgtatgctggctttgagcaatgactgtggcctcacctttgac<br>gagatcgaggacatctacccctgctctggcccgcaagcactgatcgtccaaatgtgccttcaaaatatgggcaatttcacactcctatttgaaggtgaac<br>tgcacccggacctcgatcgaggccgttttatcagtgcgtggcacaaggtagtgcaggctaatcccatgctacggacgcgattggtacccatgggcag<br>cagtagcacggatggggacttccttcaagttgtactcaagcaagcaagcgtccctgtagagagccttgacagcagaaacggtgattatgacggcaca<br>tttgacatctggggcttcaaccacccattaatacgactagcgacgctgcaggatcggtttctcgtcatgatccaccatgtggcatacgaccttttctccttc<br>gctcagctgtttgctctgctgagagaggcatatgaggggcgtgacctgccatatcgtccctatagcccgtttgtgcgatgggaacggcgctcttcacca<br>gacacagtccagttctggaagaagacatttgccggtttccagagcaagaaactctttcctcacgtcttgccgtccgcgaactacacacccacaatcaac<br>tctcaactggacgcccgatttccgatcaccatcgaagcctcggaccatggaacgctcgcctctaagctctatctggctctggcaattgccatatctcacg<br>agatagatgattctgatatcgtcttcggcggatcgttcatcagacgcggggcgcctgtgtcgggaattatggagatgatgggcccaaccacttgcatatt<br>gcccgtgcgcattcagttagatagagaggaaacgcttctaaacgcccaacaaaaggttgccaaccaactaatggaaatgtcggattttgaagatatcg<br>atactgtgcggataggaaggctgagtccagacgcgacggcagcgtgcagactgcgaaccactgttgtaatcatgcagggtgagatgcttcagagca<br>catcttatttctttgcgcgcacatggcagcataagaatatgggcttcccgacggctttgttactcatgtgctctgtatcaagtggatcagccgtggttcaag<br>ccatgtatgaccagaacatgctcaaaccggctaggatgcaacagctgttgaaccaactggcacaggcagtgcagtttattgatgaggagccacatatc<br>aaagttatgaatgtggtatcgacacagcataaggtggagagttttgagagtataaattta

Table S3. *S. cerevisiae* transformants in this study.

Transformant	Construct combination
S. cerevisiae transformants in *ane* BGC part	
Sc expressing *aneB*	pXW55H-AneB
Sc expressing *aneBE*	pXW55H-AneB+pXW06H-AneE
Sc expressing *aneB*-S596A, Q793A, Q793H, H794A, L796A, Y797A, D798A and S801A	pXW55H-AneB-S596A, pXW55H-AneB-Q793A, pXW55H-AneB-H794A, pXW55H-AneB-L796A, pXW55H-AneB-Y979A, pXW55H-AneB-D798A and pXW55H-AneB-S801A
Sc expressing *aneB* TC domain	pXW55H-AneB TC domain
S. cerevisiae transformants in *thm* BGC part	
Sc expressing *thmB*	pXW55H-ThmB
Sc expressing *thmBI*	pXW55H-ThmB+pXW06H-ThmI
Sc expressing *thmA*	pXW06H-ThmA
Sc expressing *thmBIA*	pXW55M-ThmBI+pXW06H-ThmA
S. cerevisiae transformants in combinatorial biosynthesis part	
Sc expressing *thmBI*+*aneB*	pXW55H-(ThmB+AneB)+pXW06H-ThmI
Sc expressing *thmA*(AT domain)+*aneB*(C domain)	pXW06H -ThmA(AT domain)+AneB(C domain)
Sc expressing *thmA*(A domain)+*aneB*(TC domain)	pXW06H -ThmA(A domain)+AneB(TC domain)

Table S4.^{1}H NMR (500 MHz), ^{13}C NMR (125 MHz) and 2D spectroscopic data of asperaculane H (**6)** in DMSO-*d6*.

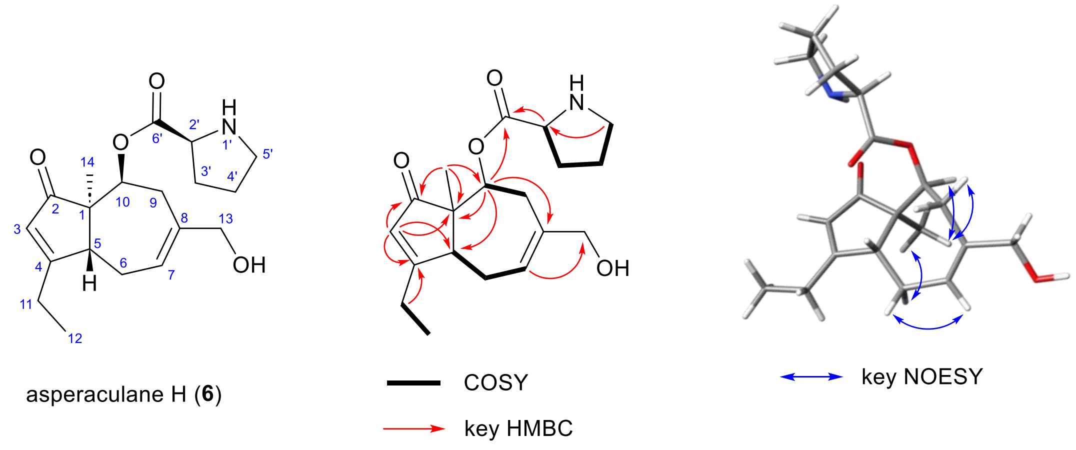

No	δ_H (mult, *J* in Hz)	δ_C (type)	HMBC (H→C)	COSY	NOESY
1		54.7 (C)			
2		208.5 (C)			
3	5.80 (q, 2.0)	123.2 (CH)	45.2, 54.7, 183.7, 208.5		1.12
4		183.7 (C)			
5	3.33 (br. dt, 13.0)	45.2 (CH)	54.7, 183.7	2.13, 2.59	
6	2.13 (t, 15.3)	24.9 (CH_2)		2.59, 3.33	0.95, 2.59
6	2.59 (m)		45.2, 54.7, 122.3, 135.9	2.13, 5.77	2.13
7	5.77 (d, 6.8)	122.3 (CH)	45.2, 31.9, 68.2	2.59	2.59, 3.70
8		135.9 (C)			
9	2.53 (m)	31.9 (CH_2)		2.40, 5.22	
9	2.40 (m)		54.7, 72.1, 122.3, 135.9	2.53	
10	5.22 (dd, 2.1, 4.9)	72.1 (CH)	45.2, 135.9, 172.6 (w)	2.40, 2.53	0.95, 2.40, 2.53
11	2.41 (m)	23.6 (CH_2)	11.2, 183.7	1.12	1.12
12	1.12 (t, 7.3)	11.2 (CH_3)	23.6, 183.7	2.41	2.41
13	3.70 (s)	68.2 (CH_2)	31.9, 122.3, 135.9		2.40, 2.53 (w), 5.77
14	0.95 (s)	17.8 (CH_3)	45.2, 54.7, 72.1, 208.5		2.13, 2.53, 5.22
2’	3.65 (q, 2.4)	59 (CH)	29.3, 46.0, 172.6	1.47, 1.83	
3’	1.47 (m)	29.3 (CH_2)	24.4, 172.6	1.83, 3.65	1.83, 3.65 (w)
	1.83 (m)		172.6	1.47, 1.59, 3.65	1.47
4’	1.59 (m)	24.4 (CH_2)			
	1.59 (m)				
5’	2.78 (m)	46.0 (CH_2)	24.4, 29.3, 59.0	1.59	
	2.89 (m)		24.4, 29.3, 59.0	1.59	
6’		172.6 (C)			

Table S5. ^{1}H NMR (500 MHz), ^{13}C NMR (125 MHz) and 2D spectroscopic data of proline phenethyl ester (**10**) in CD3OD.

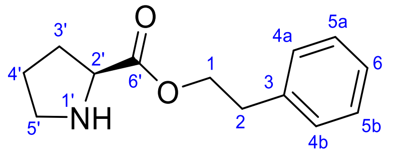

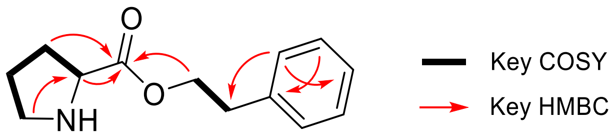

proline phenethyl ester (**10**)

No	δ_H (mult, *J* in Hz)	δ_C (type)	HMBC (H→C)	COSY
1a	4.56 (dt, 10.8,6.7)	68.2 (CH_2)	35.9, 138.9, 170.2	3.02
1b	4.44 (dt, 10.8, 6.7)			3.02
2	3.02 (t, 6.7)	35.9 (CH)	68.2, 130.1, 138.9	4.44, 4.56
3		138.9 (C)		
4a, 4b	7.26 m	130.1 (CH)	35.9, 128.0, 130.1	
5a, 5b	7.31 m	129.8 (CH)	129.8, 138.9	
6	7.23 overlapping	128.0 (CH)	130.1	
2’	4.37 (dd, 8.5, 6.3)	60.8 (CH)	24.6, 29.5, 170.2	1.99, 2.34
3a’	2.34 m	29.5 (CH_2)	24.6, 47.3, 170.2	1.99, 4.37
3b’	1.99 overlapping		24.6, 47.3, 60.8, 170.2	1.99, 4.37
4’	1.99 overlapping	24.6 (CH_2)	29.5, 47.3, 60.8	1.99, 3.33
5’	3.33 overlapping	47.3 (CH_2)	24.6, 29.5, 60.8	
6’		170.2 (C)		

Table S6. Comparison of ^{1}H NMR (500 MHz) and ^{13}C NMR (125 MHz) spectroscopic data of amorpha-4,11-diene (**11**) to reported data.[1]

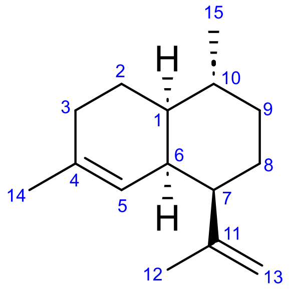

11

	In this study **11** (in $CDCl_3$) ^{1}H NMR 500 MHz, ^{13}C NMR 125 MHz		Reported **11** (in $CDCl_3$)[1] ^{1}H NMR 500 MHz, ^{13}C NMR 125 MHz	
No	δ_H (mult, *J* in Hz)	δ_C (type)	δ_H (mult, J in Hz)	δ_C (type)
1	1.30 (m)	42.0 (CH)	1.33 (m)	41.8 (CH)
2	1.94 (m)	26.0 (CH_2)	1.96 (m)	25.8 (CH_2)
	1.49 (m)		1.52 (m)	
3	1.90 (m)	26.7 (CH_2)	1.90 (m)	26.5 (CH_2)
	1.78 (m)		1.78 (m)	
4		134.8 (C)		134.6 (C)
5	5.04 (br s)	121.1 (CH)	5.06 (S)	120.9 (CH)
6	2.53 (br s)	37.8 (CH)	2.55 (S)	37.6 (CH)
7	1.97 (m)	47.8 (CH)	1.99 (d)	47.6 (CH)
8	1.49 (m)	26.3 (CH_2)	1.52 (m)	26.1(CH_2)
	1.24 (m)		1.26 (m)	
9	1.65 (m)	35.6 (CH_2)	1.67 (m)	35.4 (CH_2)
	0.96 (m)		0.97 (m)	
10	1.40 (m)	28.1 (CH)	1.40 (m)	27.9 (CH)
11		148.2 (C)		148.0 (C)
12	1.72 (br s)	22.8 (CH_3)	1.74 (S)	22.6 (CH_3)
13	4.85 (m)	110.0 (CH_2)	4.87 (d, *J*=1.2)	109.8 (CH_2)
13’	4.62 (m)		4.65 (S)	
14	1.58 (br s)	23.9 (CH_3)	1.61 (S)	23.7 (CH_3)
15	0.87 (d, *J*=6.5)	20.1 (CH_3)	0.89 (d, *J*=6.7)	19.8 (CH_3)

Table S7. Comparison of ^{1}H NMR (500 MHz) and ^{13}C NMR (125 MHz) spectroscopic data of amorpha-4,11-diene-2-ol (**12**) to the reported data.[2]

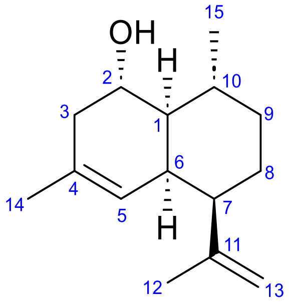

12

	In this study **12** (in $CDCl_3$) ^{1}H NMR 500 MHz, ^{13}C NMR 125 MHz		Reported **12** (in $CDCl_3$)[2] ^{1}H NMR 500 MHz, ^{13}C NMR 125 MHz	
No	δ_H (mult, *J* in Hz)	δ_C (type)	δ_H (mult, J in Hz)	δ_C (type)
1	1.40 (m)	48.1 (CH)	1.42 (m)	47.9 (CH)
2	4.19 (br s)	67.6 (CH)	4.21 (s)	67.4 (CH)
3	2.20 (d, 18.0)	35.2 (CH_2)	2.22 (d, 18.0)	35.0 (CH_2)
	1.86 (d, 18.0)		1.88 (d, 18.0)	
4		131.2 (C)		131.1 (C)
5	5.14 (s)	120.8 (CH)	5.16 (s)	120.6 (CH)
6	2.76 (s)	31.3 (CH)	2.78 (s)	31.1 (CH)
7	1.95 (d, 12.5)	46.8 (CH)	1.96 (d, 12.5)	46.6 (CH)
8	1.52 (m)	26.3 (CH_2)	1.54 (d, 12.5)	26.0 (CH_2)
	1.27 (m)		1.29 (m)	
9	1.61 (m)	35.7 (CH_2)	1.65 (m)	35.5 (CH_2)
	0.99 (m)		1.01 (m)	
10	1.16 (m)	30.0 (CH)	1.76 (m)	29.7 (CH)
11		148.0 (C)		147.8 (C)
12	1.75 (s)	22.9 (CH_3)	1.76 (s)	22.7(CH_3)
13	4.88 (br s)	110.4 (CH_2)	4.90 (s)	110.2 (CH_2)
13’	4.66 (s)		4.68 (s)	
14	1.61 (s)	23.6 (CH_3)	1.63 (s)	23.7 (CH_3)
15	0.88 (d, 6.5)	20.0 (CH_3)	0.89 (d, 6.7)	19.8 (CH_3)

Table S8. Comparison of ^{1}H NMR (500 MHz) and ^{13}C NMR (125 MHz) spectroscopic data of tryptophan phenethyl ester (**16**) to the reported data.[3]

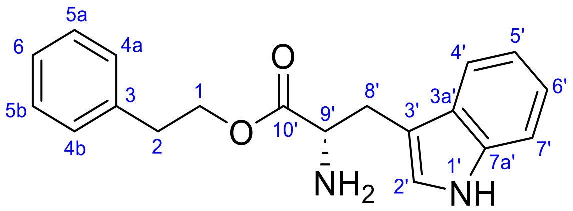

tryptophan phenethyl ester (**16**)

	In this study **16** (in DMSO-*d6*) ^{1}H NMR 500 MHz, ^{13}C NMR 125 MHz		Reported **16** (in DMSO-*d6*)[3] ^{1}H NMR 400 MHz, ^{13}C NMR 100 MHz	
No	δ_H (mult, *J* in Hz)	δ_C (type)	δ_H (mult, *J* in Hz)	δ_C (type)
1	4.22 (dt, 6.7, 2.3)	65.8 (CH_2)	4.22 (t, 6.8)	66.2 (CH_2)
2	2.76 (m)	34.0 (CH)	2.75 (m)	34.0 (CH_2)
3		137.6 (C)		137.6 (C)
4a, 4b	7.17 (d, 7.1)	128.9 (CH)	7.16 (m)	129.0 (CH)
5a, 5b	7.27 (t, 7.4)	128.4 (CH)	7.28 (t, 7.2)	128.5 (CH)
6	7.21 (t, 7.2)	126.5 (CH)	7.22(t, 6.4)	126.6 (CH)
2’	7.10 (m)	124.5 (CH)	7.16 (m)	124.9 (CH)
3’		107.2 (C)		106.5 (C)
3a’		127.0 (C)		127.0 (C)
4’	7.39 (d, 7.9)	118.0 (CH)	7.41 (d, 7.9)	118.0 (CH)
5’	7.00 (t, 7.4)	118.6 (CH)	7.00 (t, 7.0)	118.7 (CH)
6’	7.09 (m)	121.1 (CH)	7.10 (t, 7.1)	121.3(CH)
7’	7.37 (d, 8.1)	111.5 (CH)	7.38 (d, 8.1)	111.6 (CH)
7a’		136.2(C)		136.3 (C)
8’	3.12 (d, 6.5)	27.3 (CH_2)	3.24 (m)	26.3 (CH_2)
9’	4.07 (t, 6.7)	53.3 (CH)	4.17 (t, 6.4)	52.8 (CH)
10’		170.8 (C)		169.5 (C)

3 Supplementary Figures

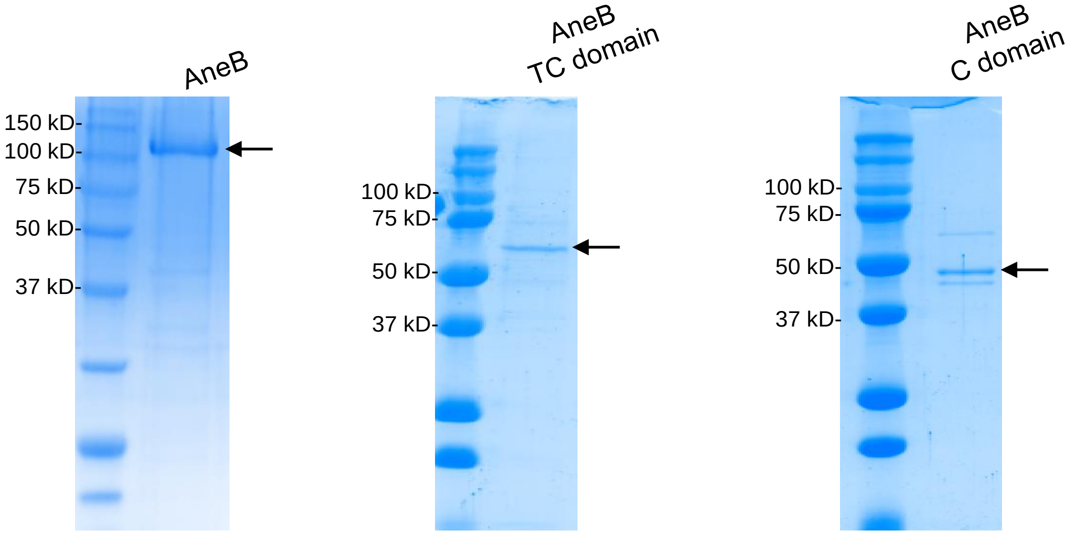

Fig. S1. SDS-PAGE of purified proteins. (A) AneB (120.5 kDa), (B) AneB T-C domain (59.8 kDa), (C) AneB C domain (48.9 kDa).

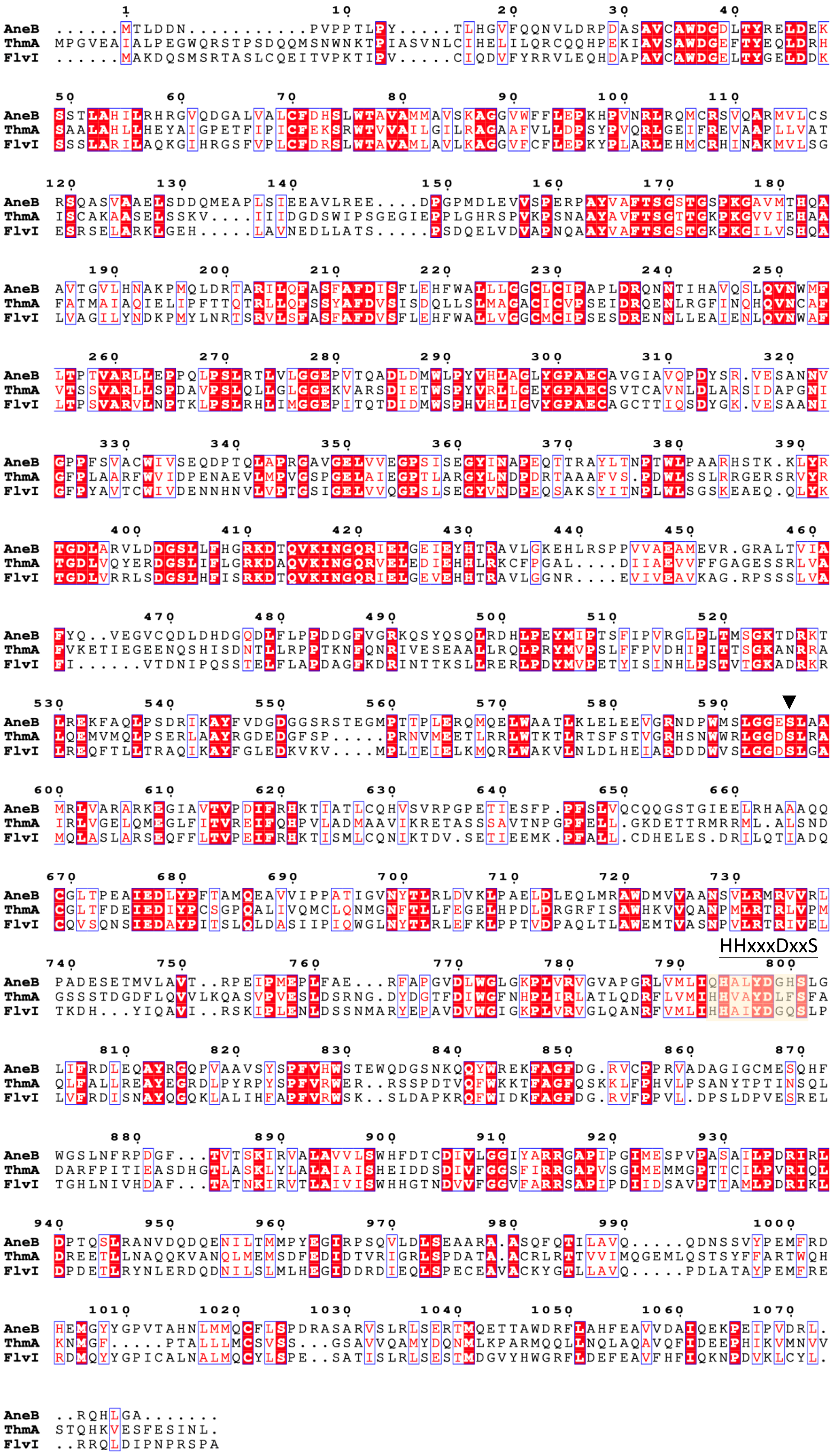

Fig. S2. Sequence alignment of single-modular NRPSs AneB, ThmA and FlvI.

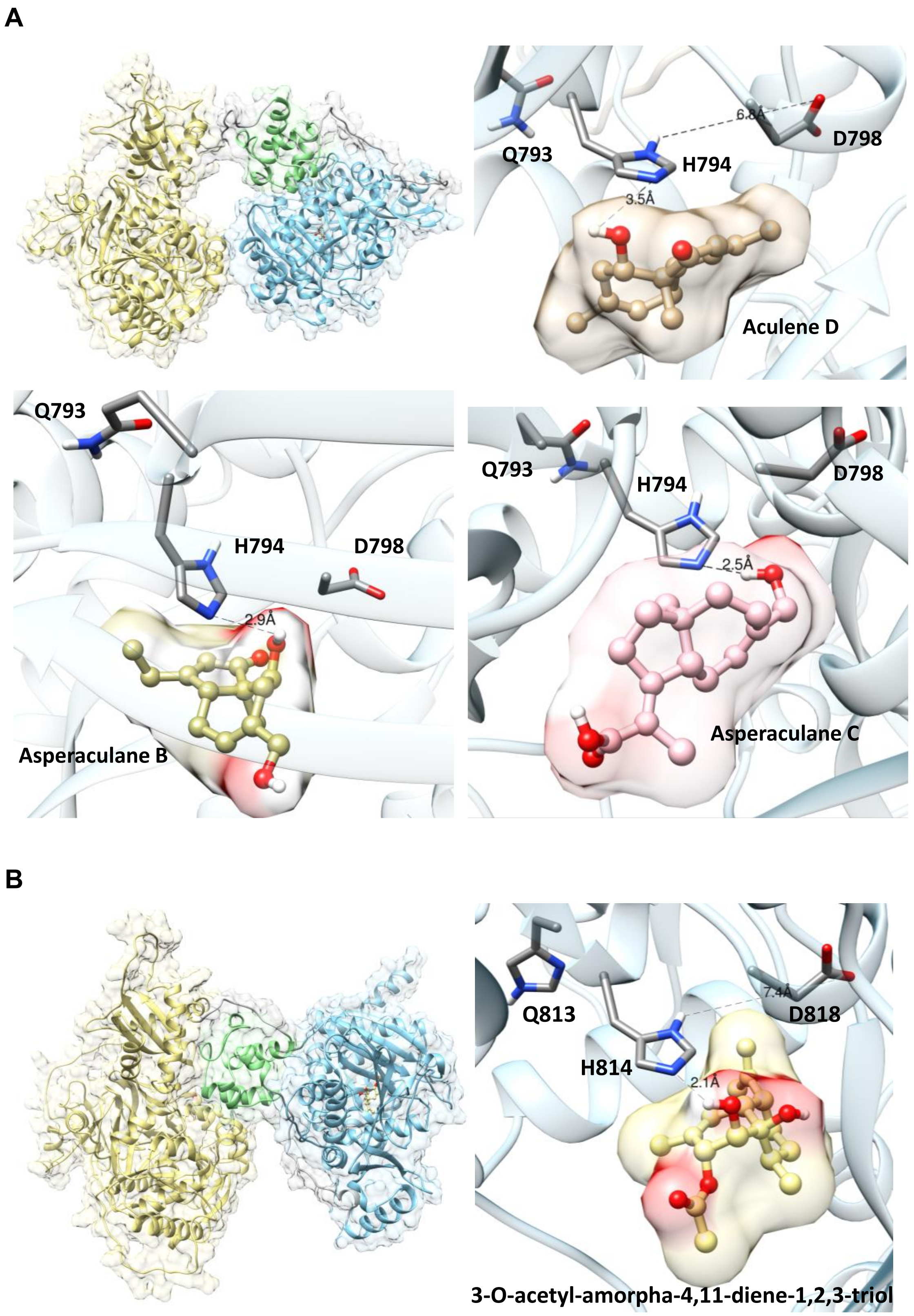

Fig. S3. Predicted structures by AlphaFold and simulated substrate binding of (A) AneB with aculene D (**4**), asperaculane B (**5**) and asperaculene C (**7**), and (B) ThmA 3-*O*-acetyl-amorpha-4,11-diene-1,2,3-triol (yellow: A domain, green: T domain, blue: C domain).

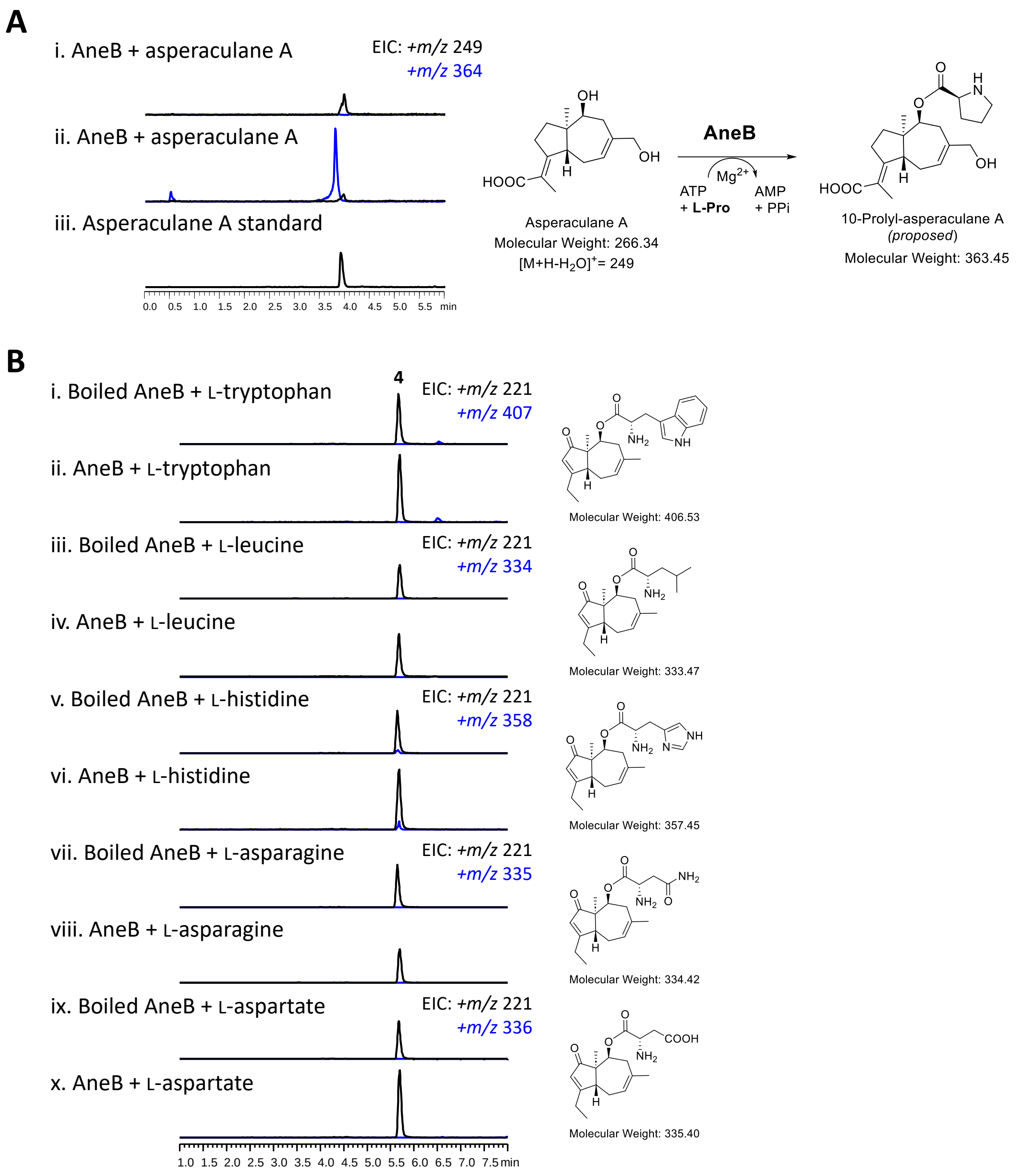

Fig. S4. LC-MS analysis of *in vitro* assays of AneB and (A) asperaculane A with L-proline, and (B) **4** with L-tryptophan, L-leucine, L-histidine, L-asparagine and L-aspartate, respectively

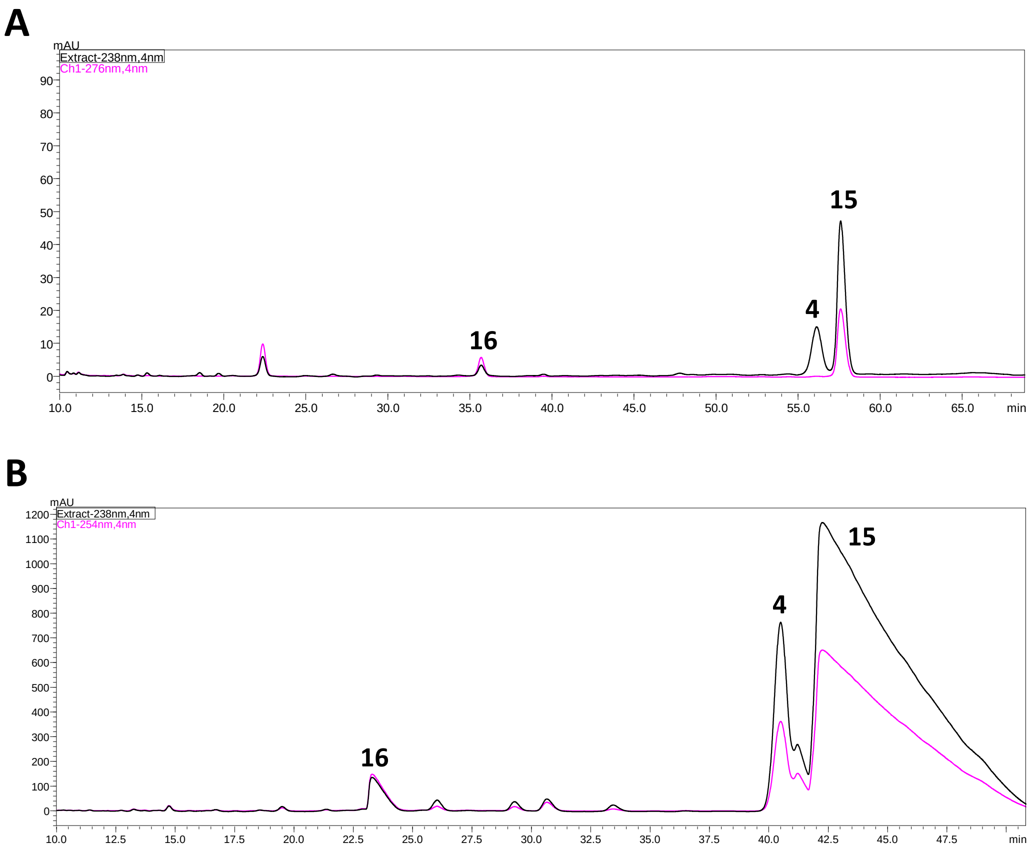

Fig. S5. HPLC profiles of (A) *S. cerevisiae* expressing *chimera 1* supplemented with **4** analyzed by (A) an analytical column (Luna, 5µm, 18C(2), 250 x 4.6 mm), eluted by 30% to 38% acetonitrile/water containing 0.1% trifluoroacetic acid at a flow rate of 0.42 mL/min), and (B) a semi-preparative column (Luna, 5µm, 18C(2), 250 x 10 mm), eluted by 35% acetonitrile/water containing 0.1% trifluoroacetic acid at a flow rate of 2.0 mL/min). The ethyl acetate extracts (injected 15 µL, 5.3 mg/100 µL) of the culture medium and the cells from 1 L culture (concentrated to 15 mL and supplemented with 12.0 mg of **4**) was analyzed. Detailed methods for sample preparation of heterologous reconstitution and biotransformation by *S. cerevisiae* are described in Materials and Methods.

A. Chimera 1

		T domain / Linker region / C domain	
AneB	627	L C Q H V S V R P G P E T I E S F P · P F S L V Q C Q Q G S T G I E E L	661
ThmA	644	M A A V I K R E T A S S S A V T N P G P F E L L · G K D E T T R M R R M	678
FlvI	620	L C Q N I K T D V · S E T I E E M K · P F A L L · C D H E L E S · D R I	651

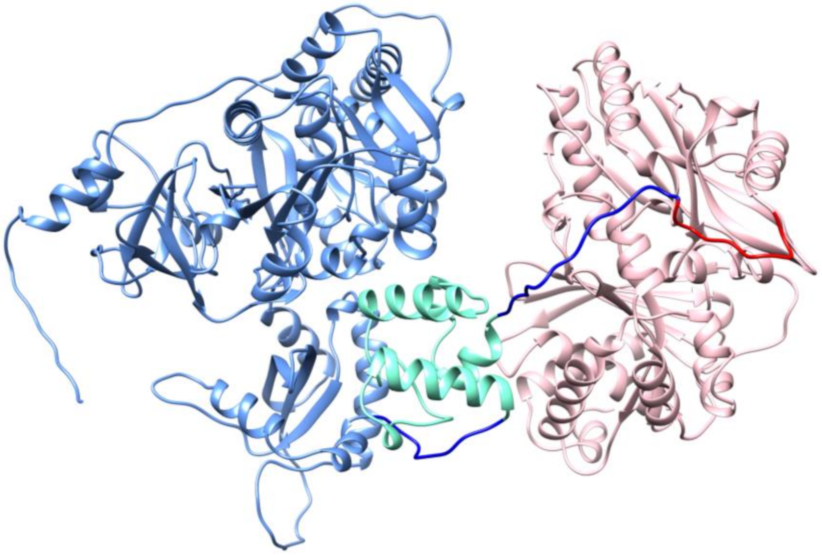

B. Chimera 2

		A domain / Linker region / T domain	
AneB	544	A Y F V D G D G G S R S T E G M P T T P L E	565
ThmA	566	A Y R G D E D G F S P · · · · · P R N V M E	579
FlvI	541	A Y F G L E D K V K V · · · · M P L T E I E	558

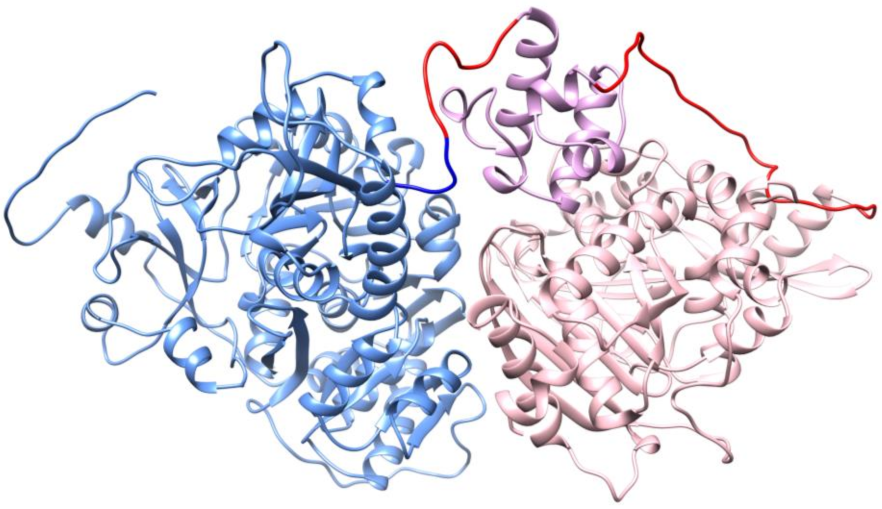

Fig. S6. Sequence alignment of the predicted linker regions between A and T domains, and T and C domain of AneB, ThmA and FlvI. Based on the alignments, chimeras 1 and 2 designed in this study are $ThmA_{1\text{-}664}$-$AneB_{647\text{-}1078}$ and $ThmA_{1\text{-}573}$-$AneB_{552\text{-}1078}$.

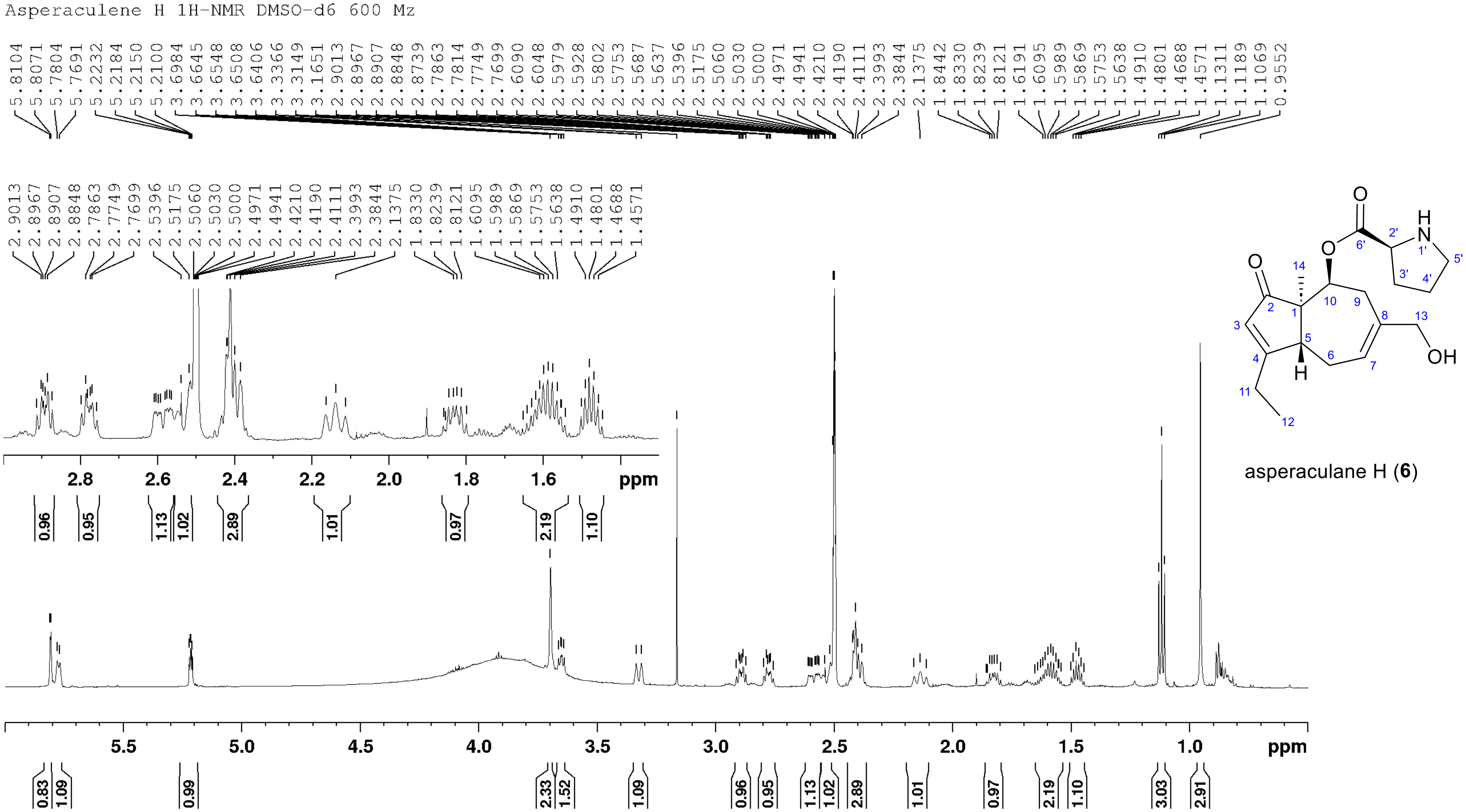

Fig. S7. ^{1}H NMR spectrum (DMSO-*d6*, 600 MHz) of **6**.

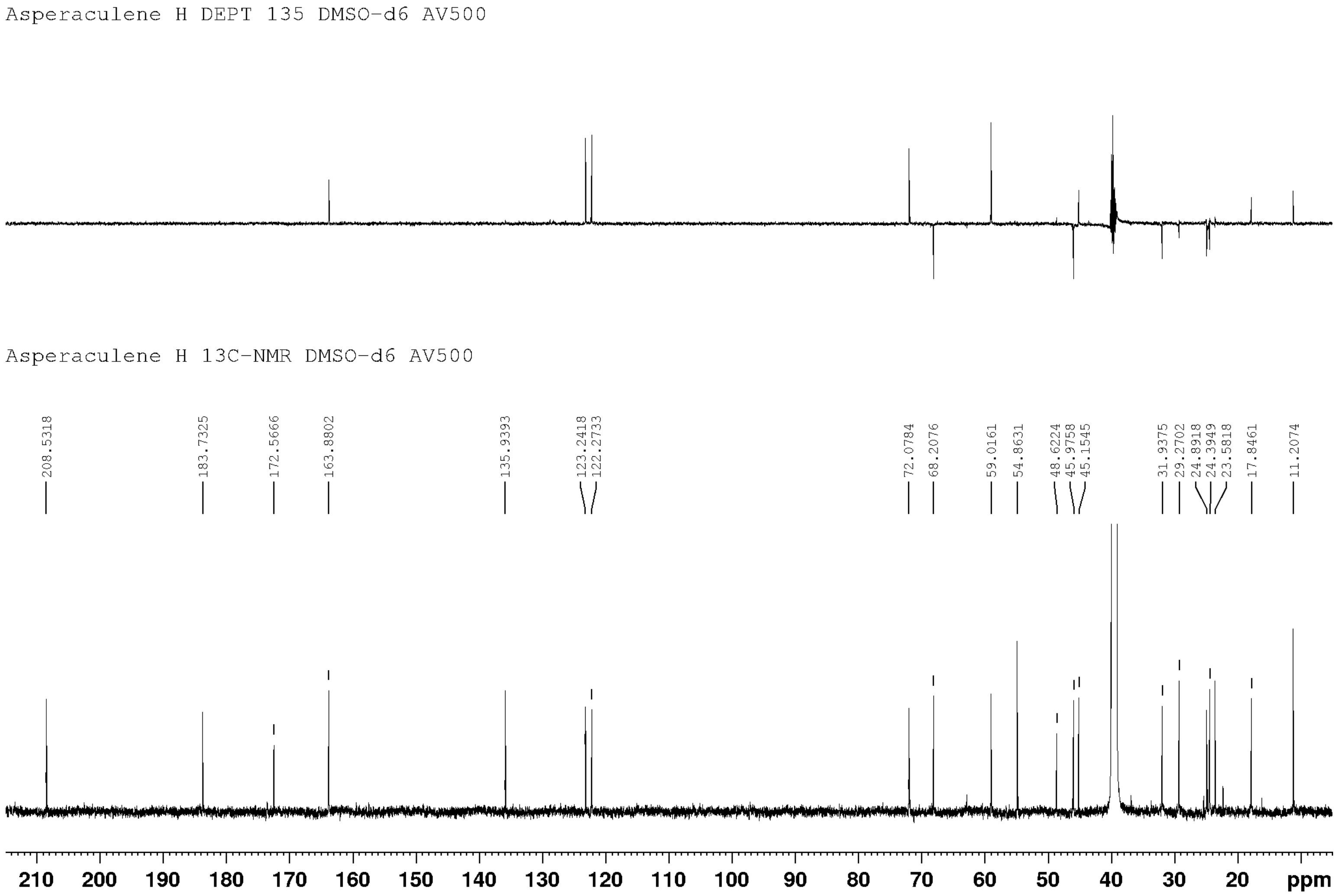

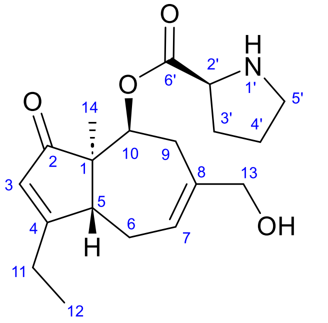

asperaculane H (**6**)

Fig. S8. ^{13}C NMR and DEPT135 spectra (DMSO-d_6, 125 MHz) of **6**.

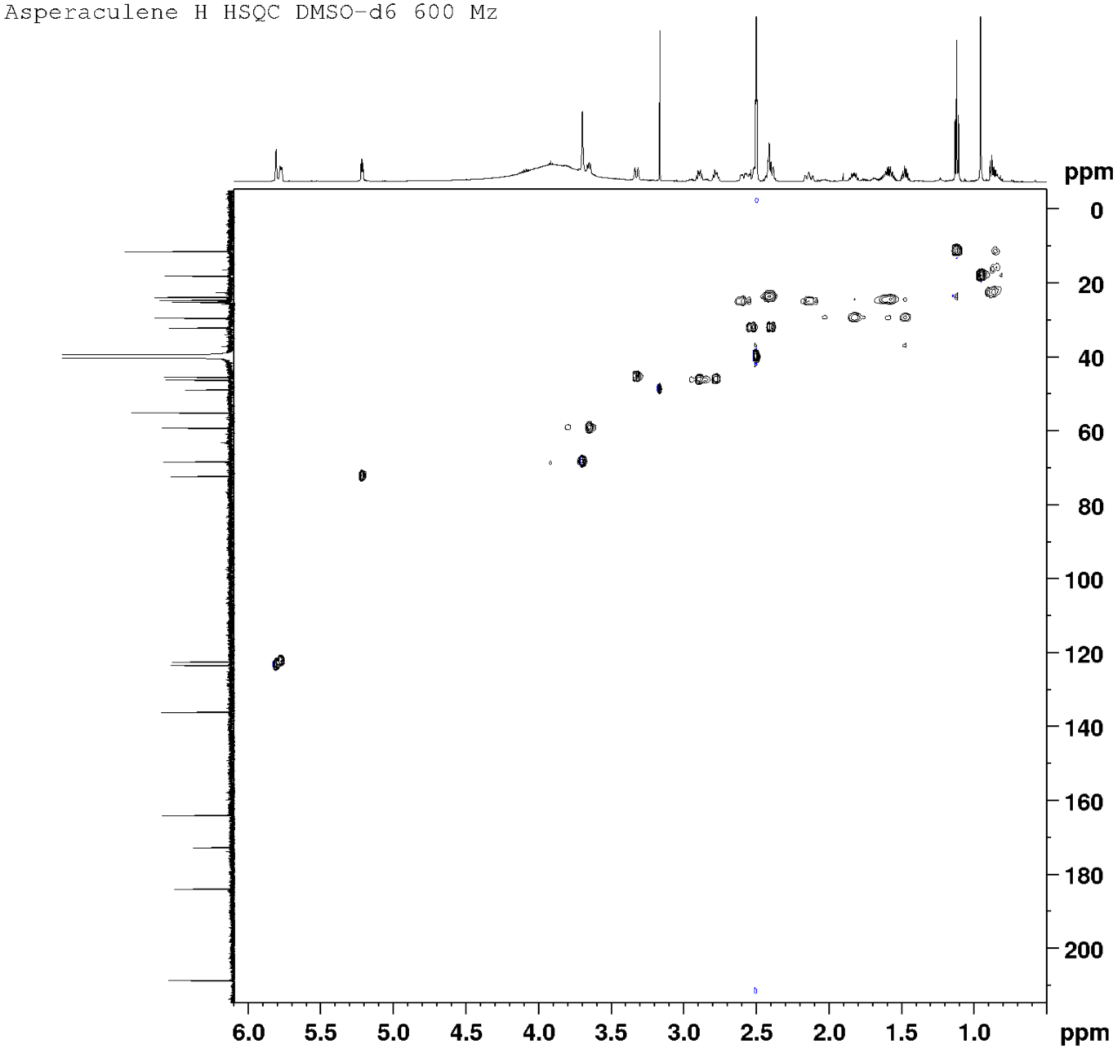

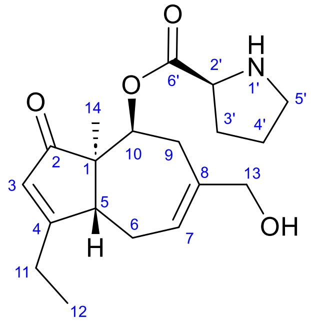

asperaculane H (**6**)

Fig. S9. HSQC spectrum (DMSO-*d6*, 500 MHz) of **6**.

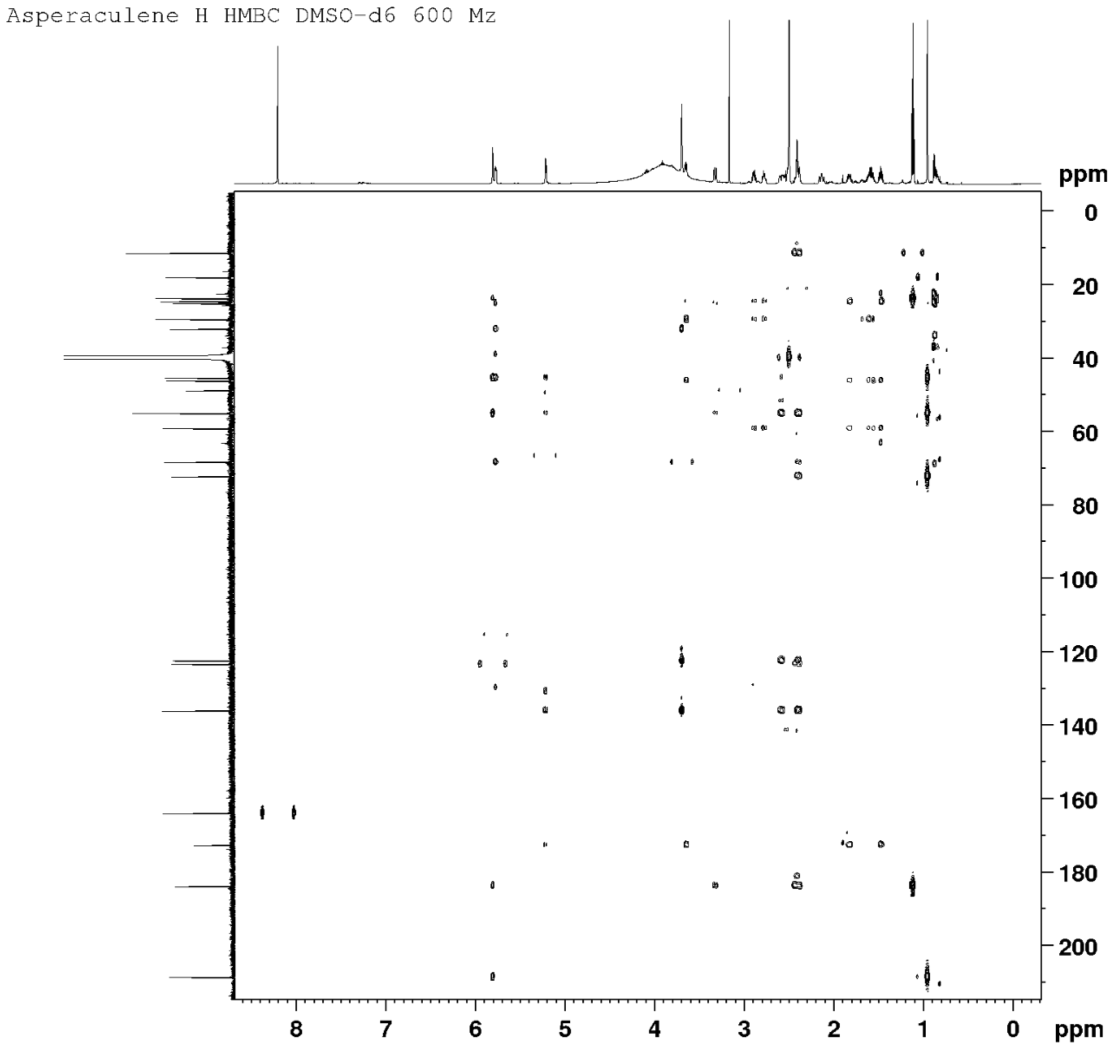

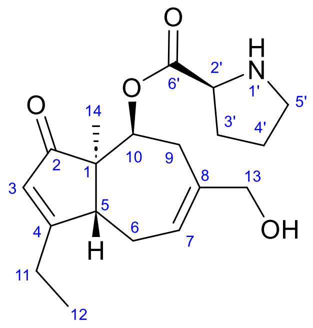

asperaculane H (**6**)

Fig. S10. HMBC spectrum (DMSO-d_6, 500 MHz) of **6**.

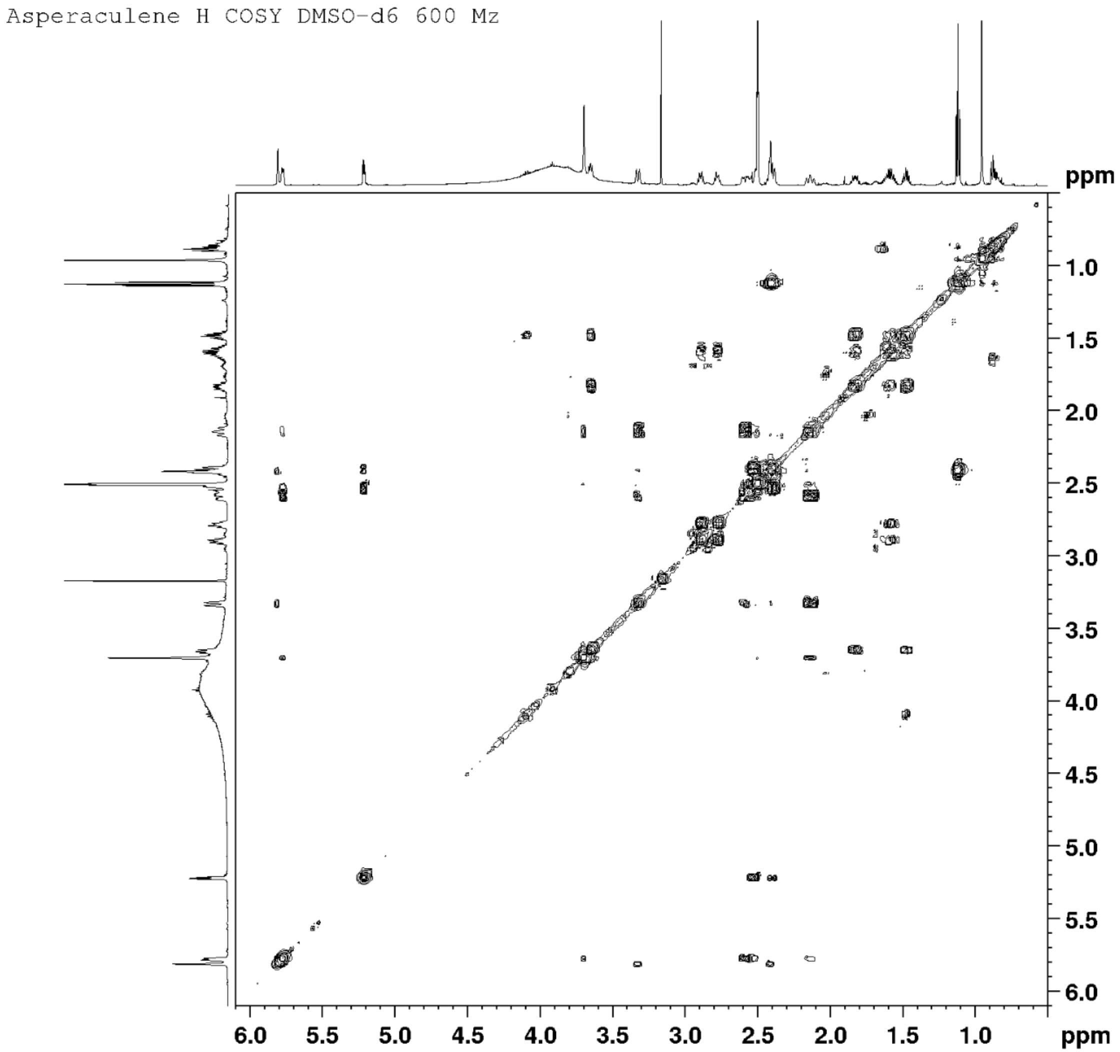

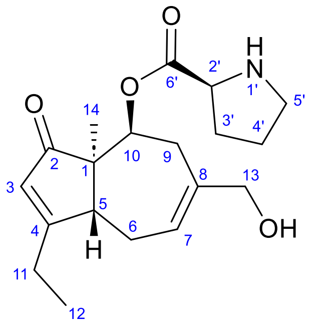

asperaculane H (**6**)

Fig. S11. COSY spectrum (DMSO-d_6, 500 MHz) of **6**.

Asperaculene H NOESY DMSO-d6 600 Mz

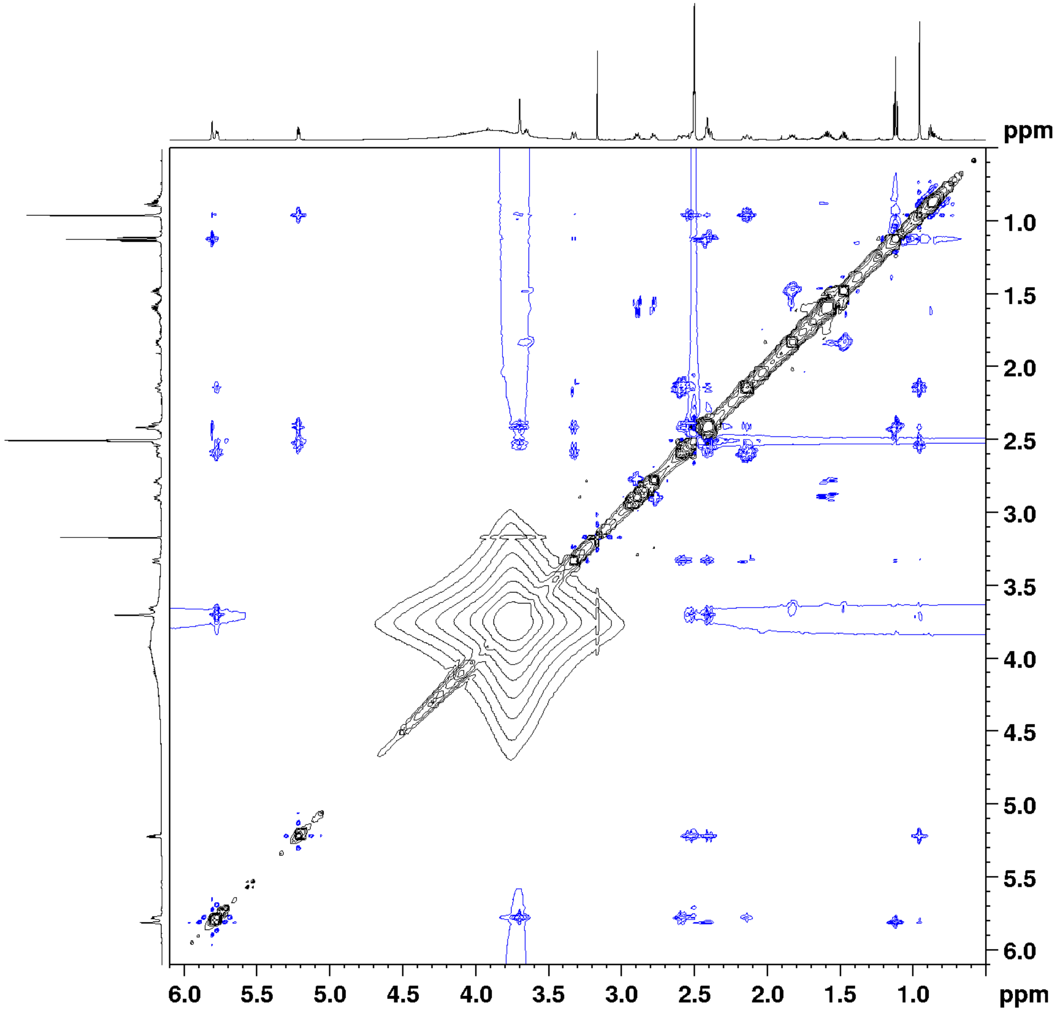

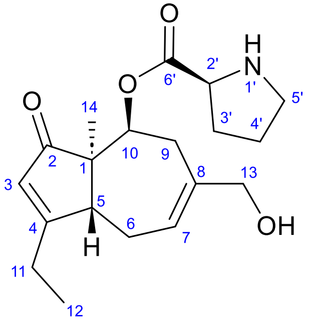

asperaculane H (**6**)

Fig. S12. NOESY spectrum (DMSO-d_6, 500 MHz) of **6**.

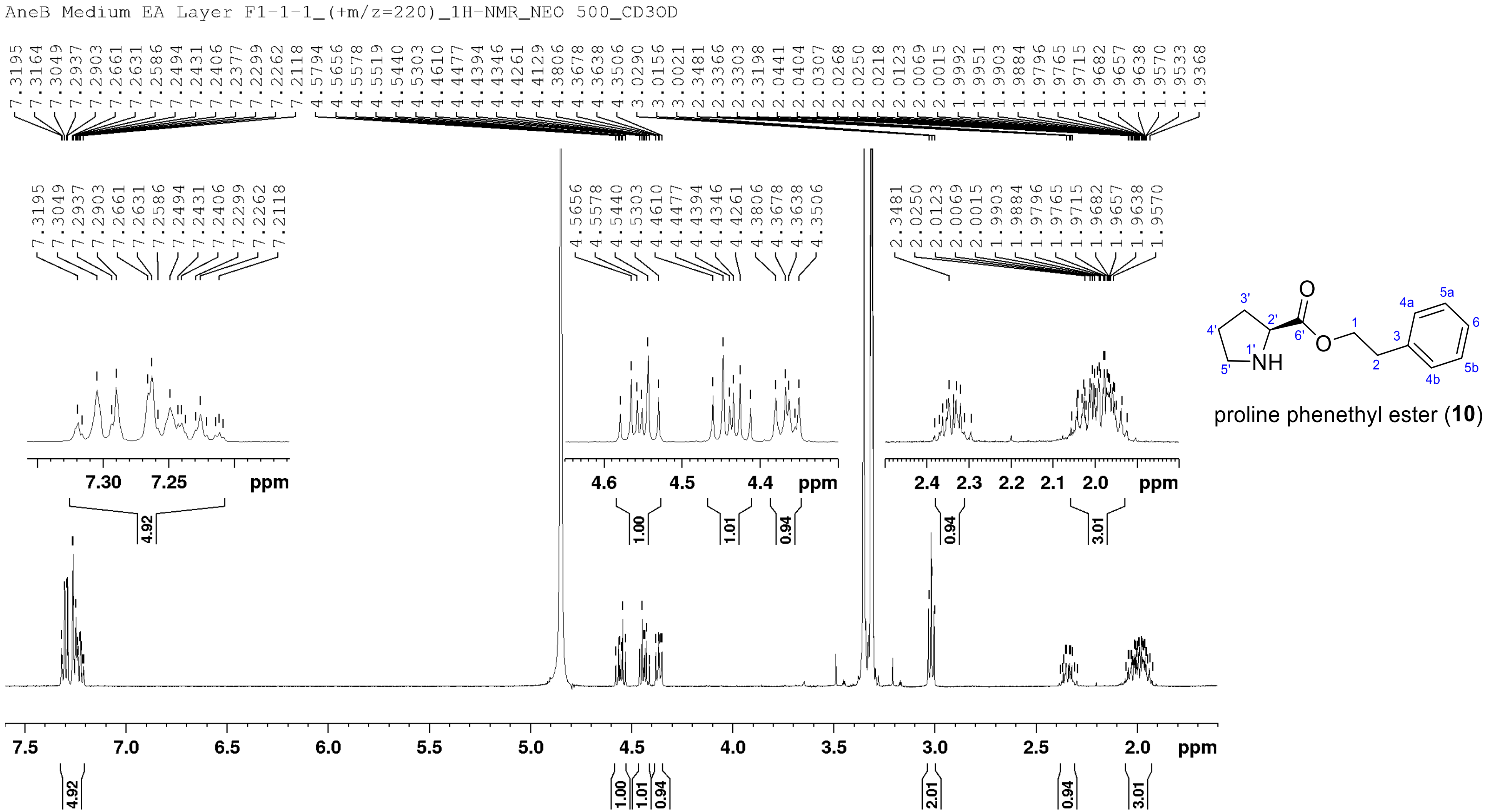

Fig. S13. ^{1}H NMR spectrum (CD_3OD, 500 MHz) of **10.**

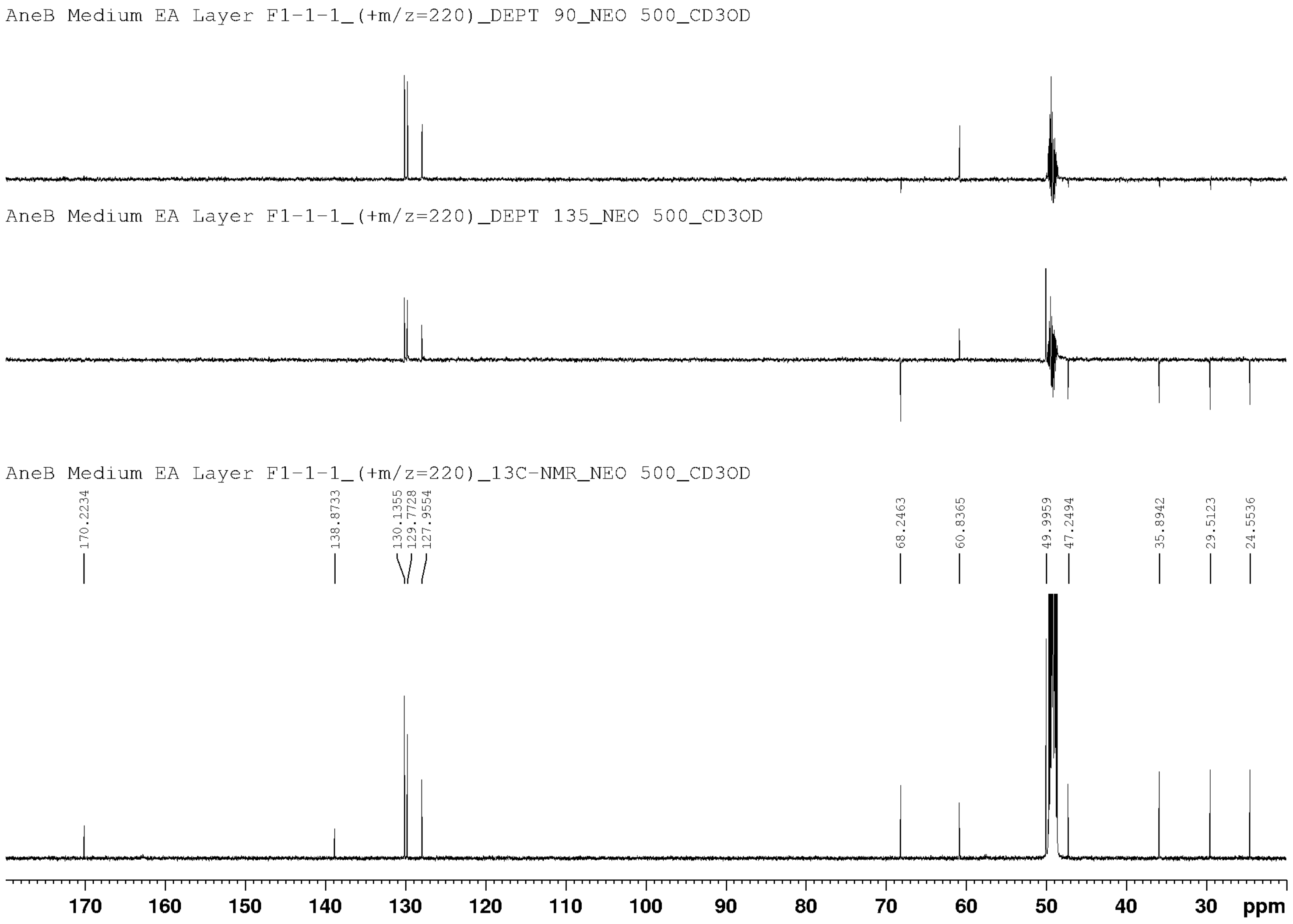

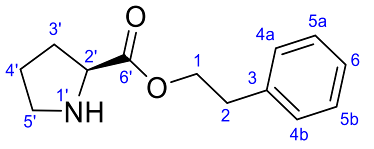

proline phenethyl ester (**10**)

Fig. S14. ^{13}C NMR, DEPT135 and DEPT90 spectra (CD_3OD, 125 MHz) of **10**.

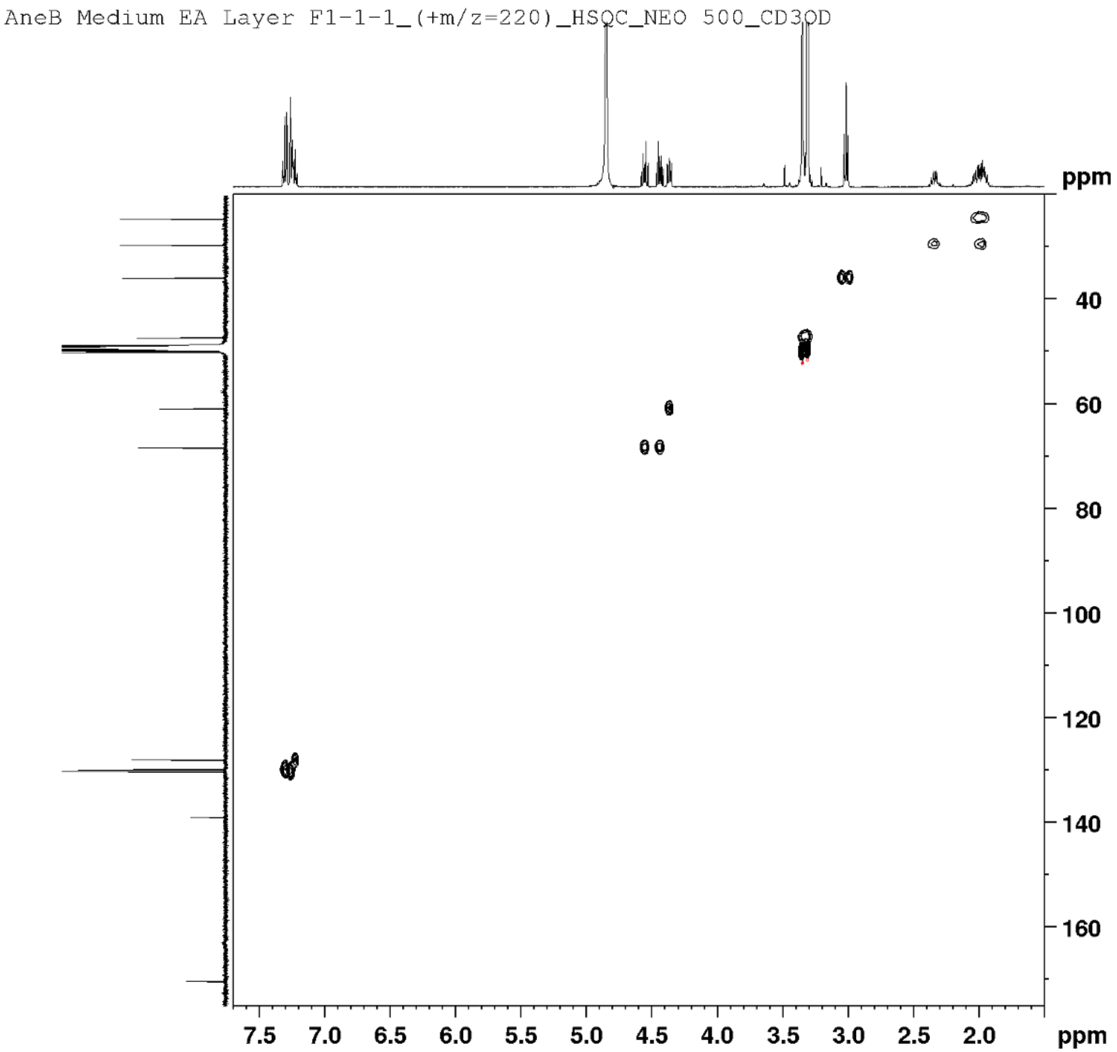

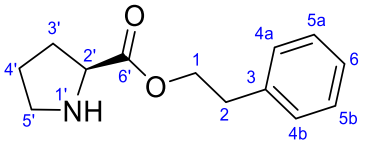

proline phenethyl ester (**10**)

Fig. S15. HSQC spectrum (CD_3OD, 500 MHz) of **10**.

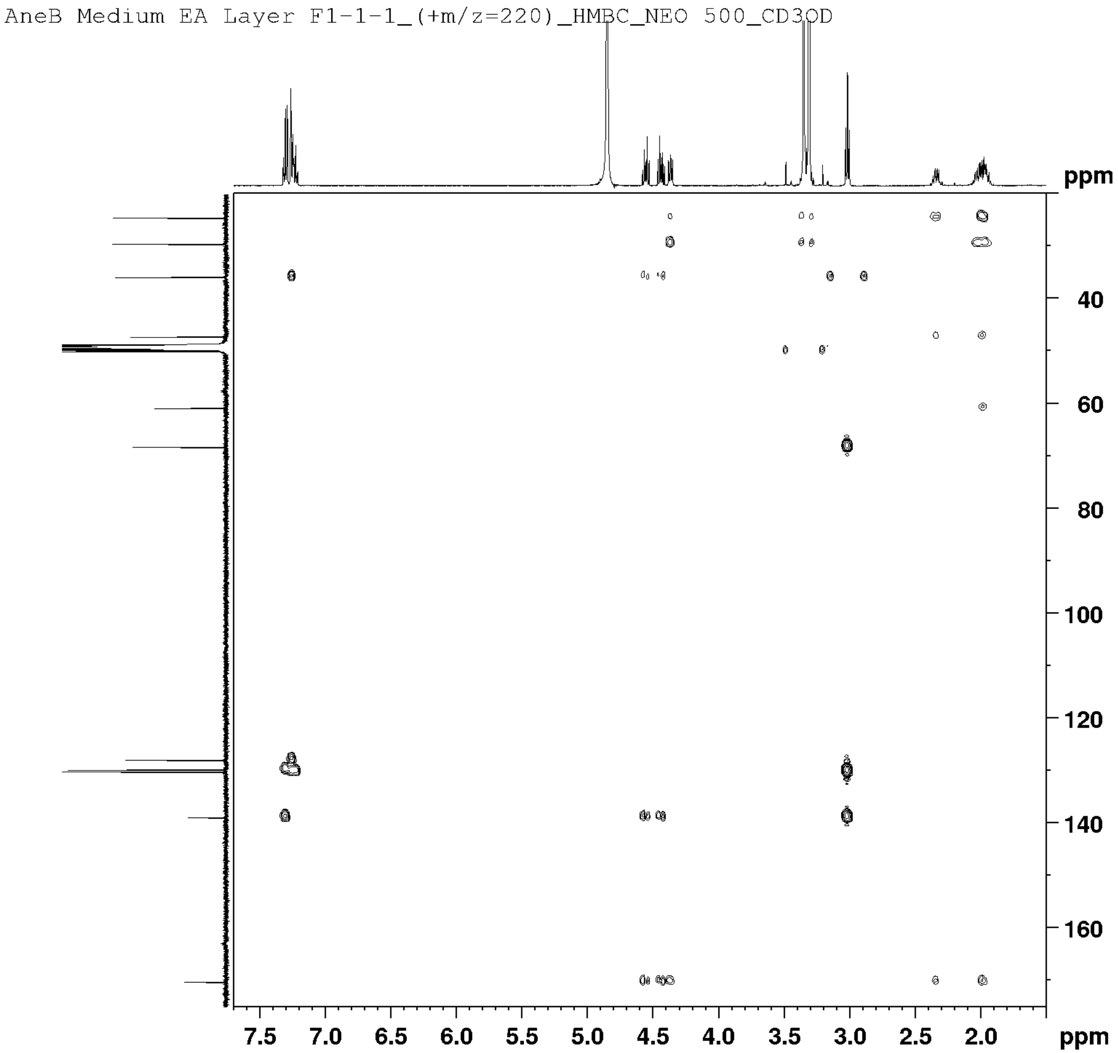

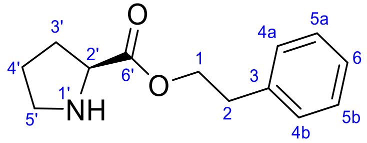

proline phenethyl ester (**10**)

Fig. S16. HMBC spectrum (CD_3OD, 500 MHz) of **10**.

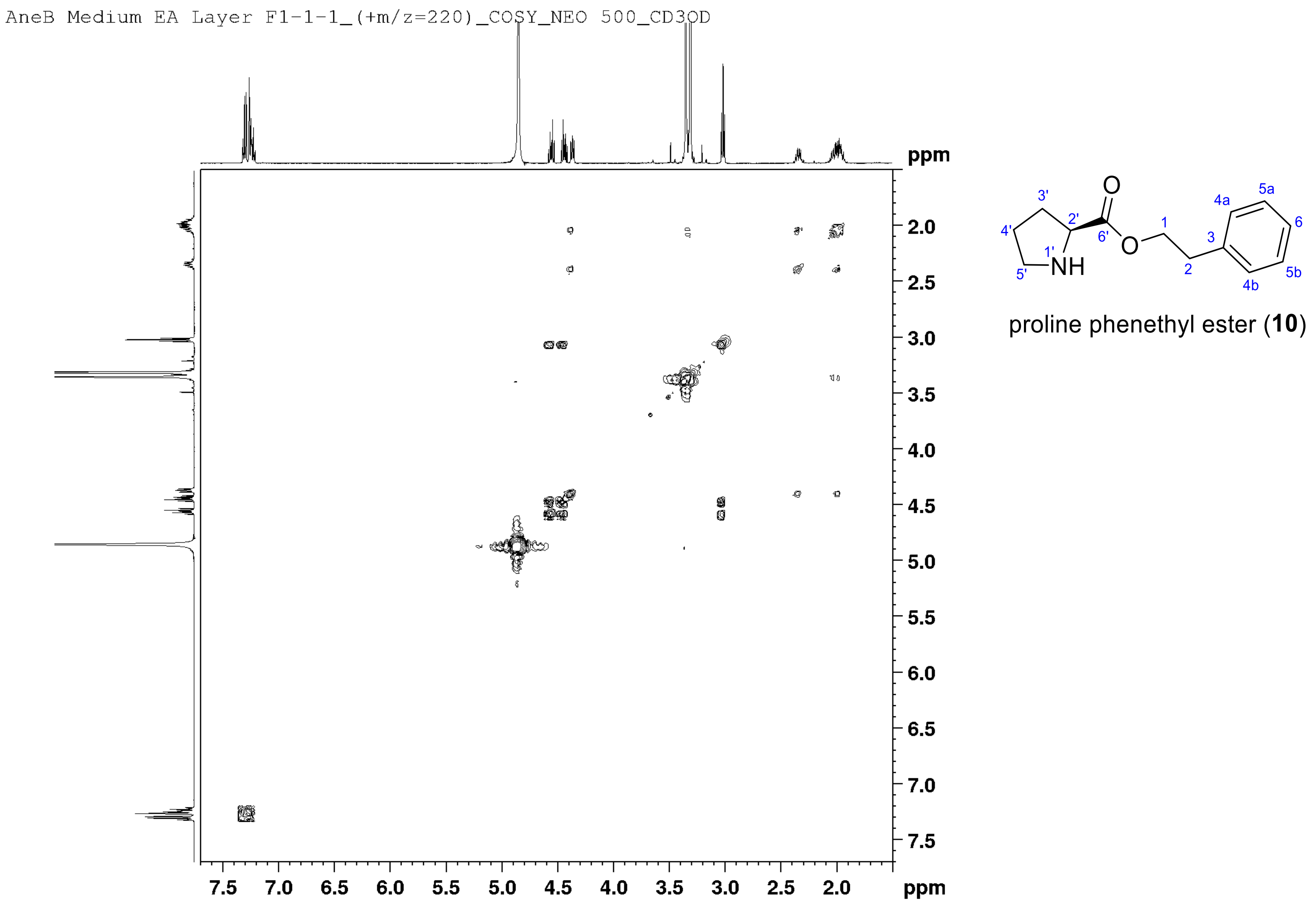

Fig. S17. COSY spectrum (CD_3OD, 500 MHz) of **10**.

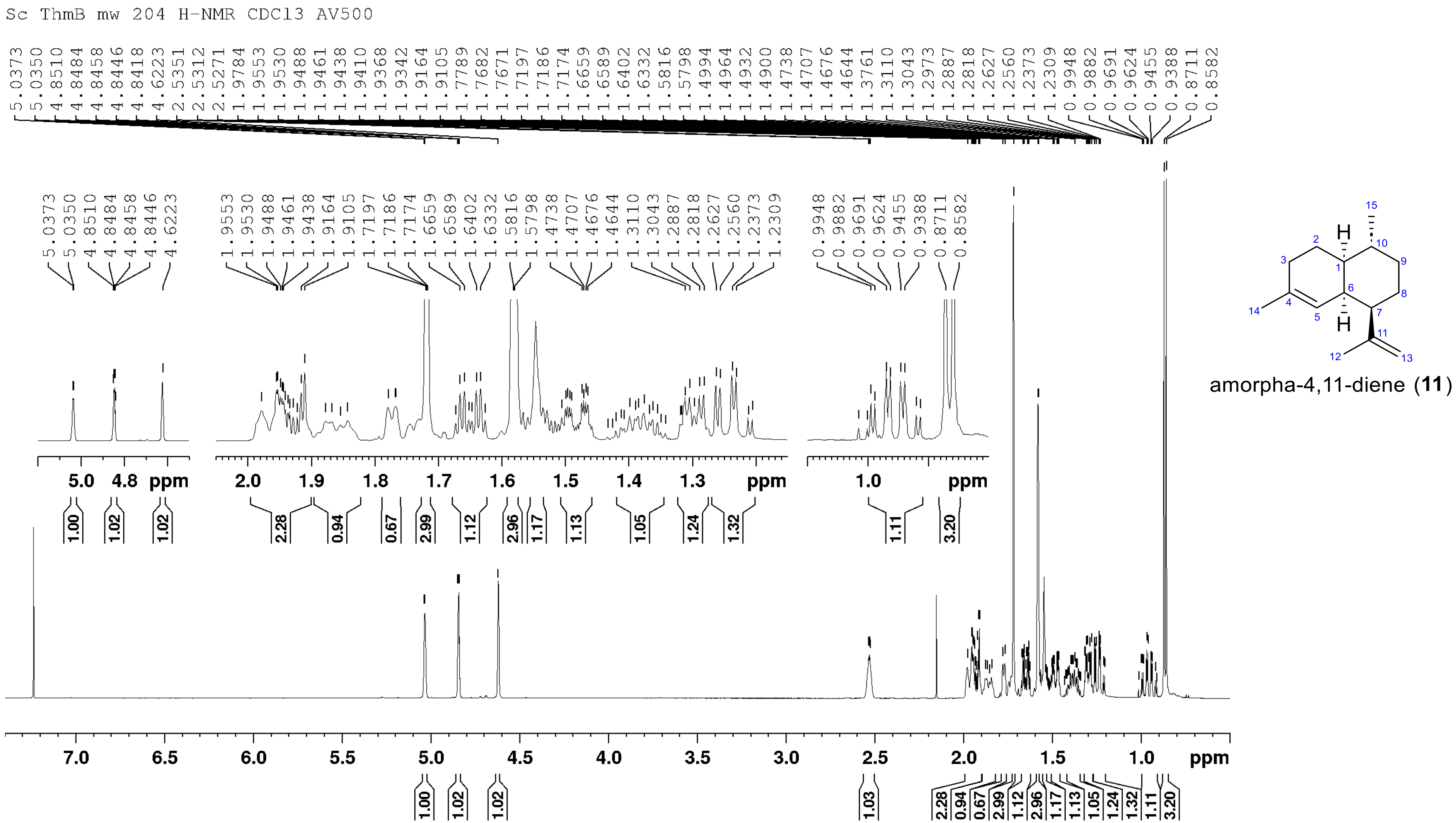

Fig. S18. ^{1}H NMR spectrum (CDCl$_3$, 500 MHz) of **11**.

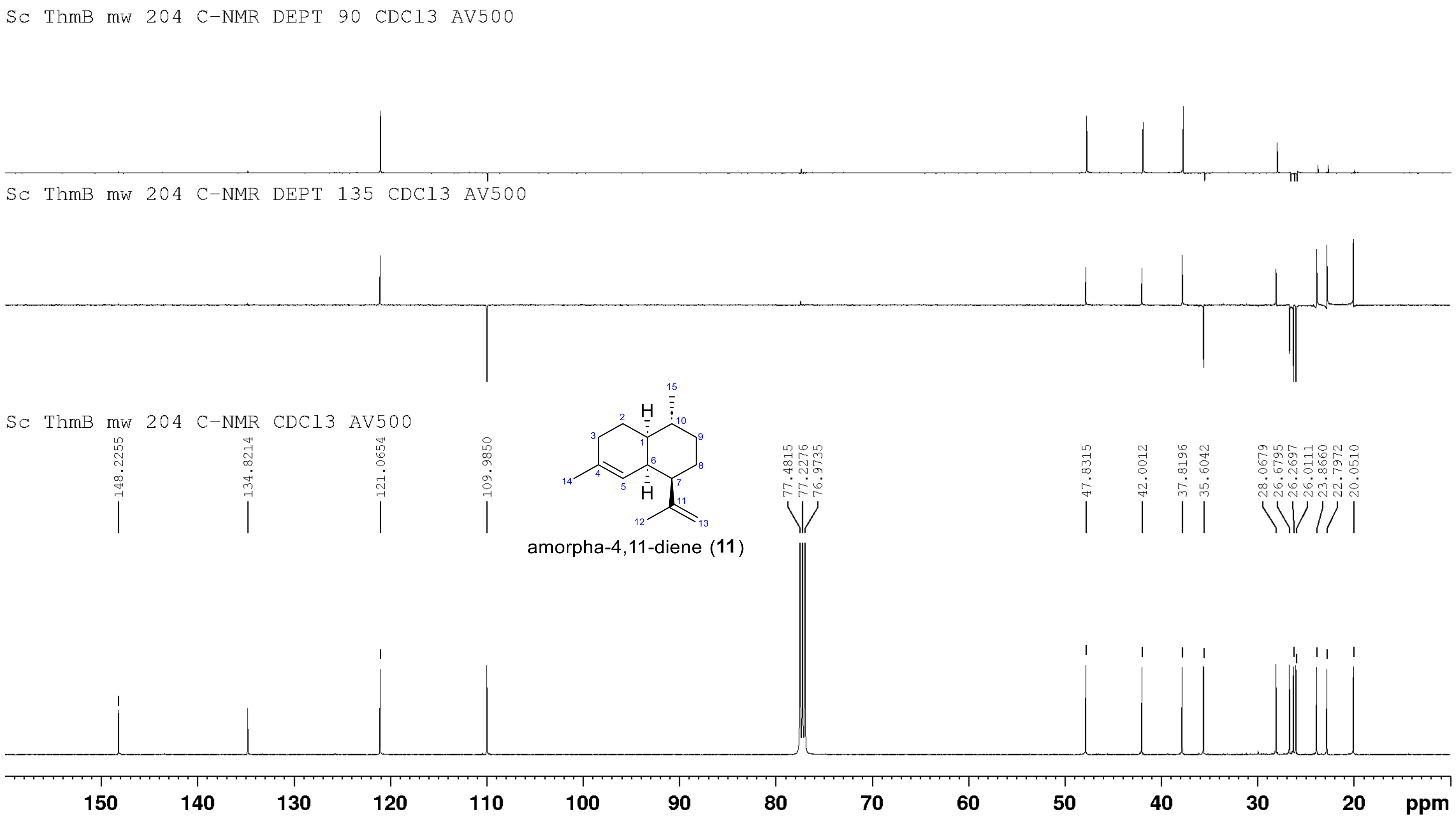

Fig. S19. ^{13}C NMR, DEPT 135 and DEPT 90 spectra ($CDCl_3$, 125 MHz) of **11**.

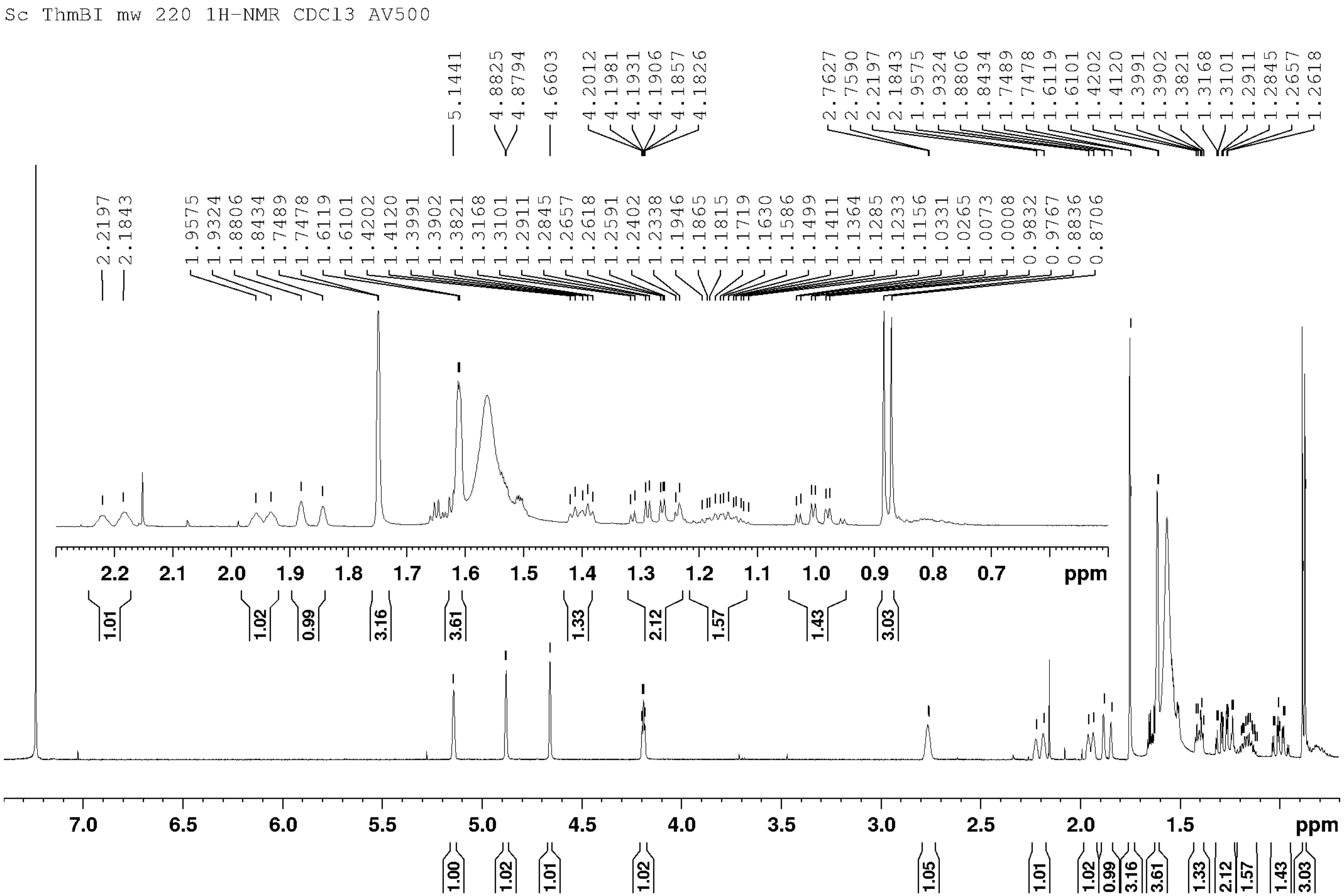

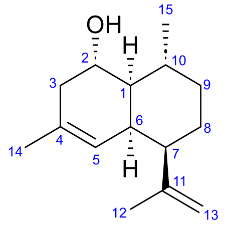

amorpha-4,11-diene-2-ol (**12**)

Fig. S20. ^{1}H NMR spectrum ($CDCl_3$, 500 MHz) of **12**.

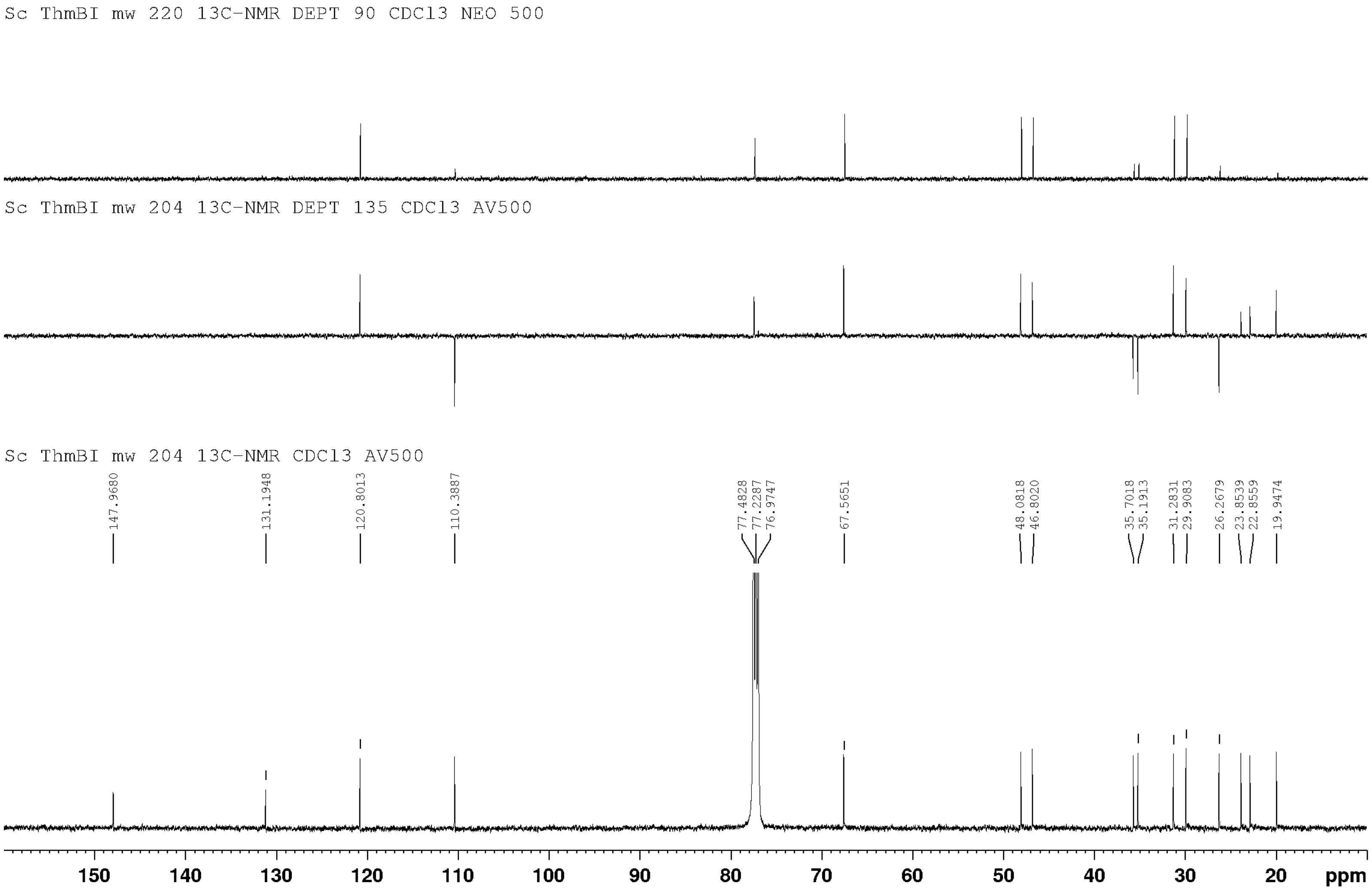

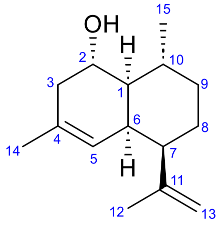

amorpha-4,11-diene-2-ol (**12**)

Fig. S21. ^{13}C NMR, DEPT 135 and DEPT 90 spectra ($CDCl_3$, 125 MHz) of **12**.

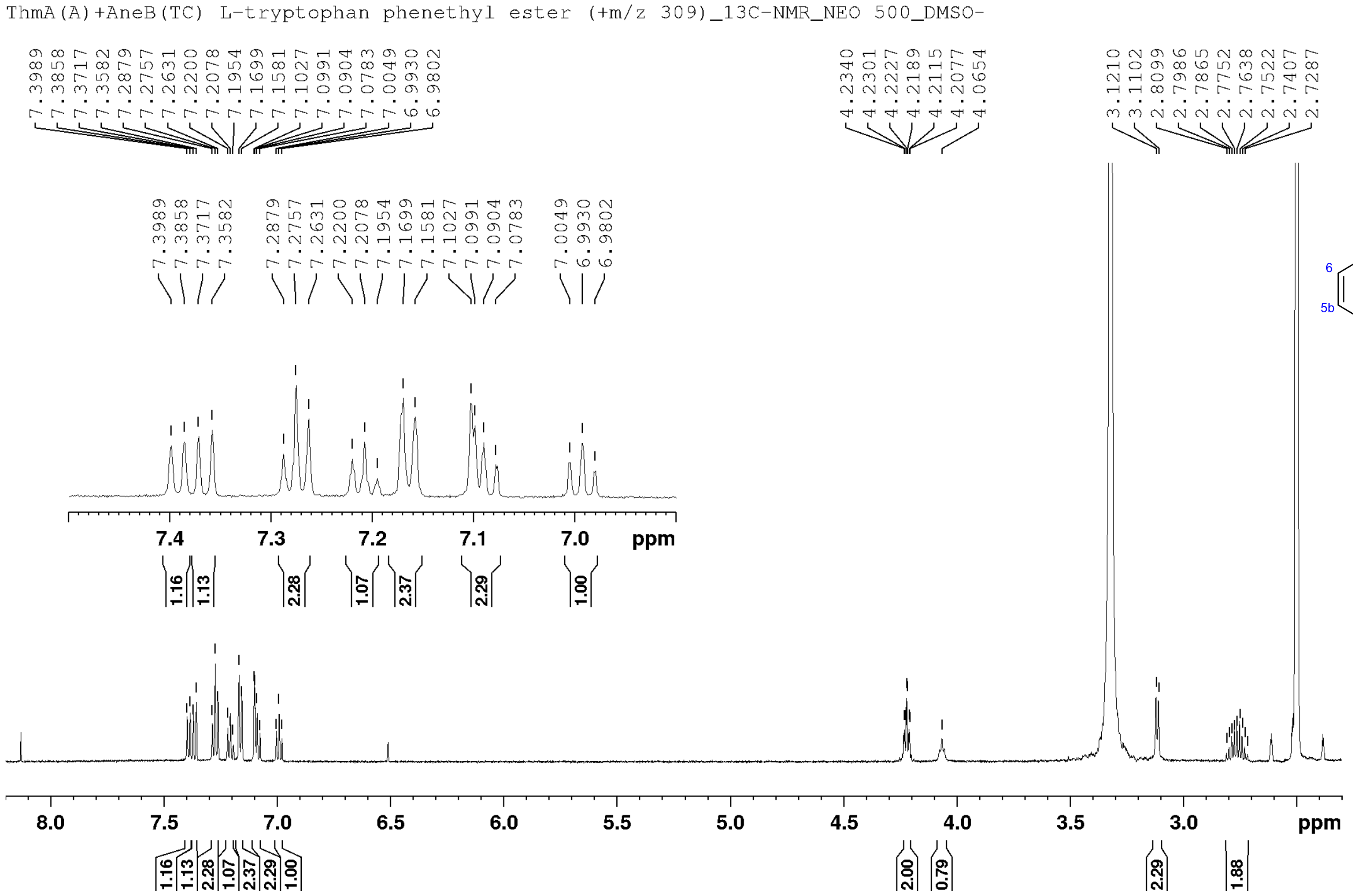

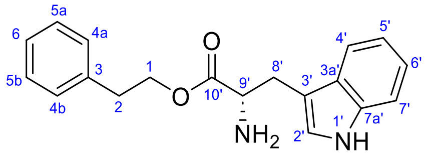

tryptophan phenethyl ester (**16**)

Fig. S22. ^{1}H NMR spectrum (DMSO-d_6, 500 MHz) of **16**.

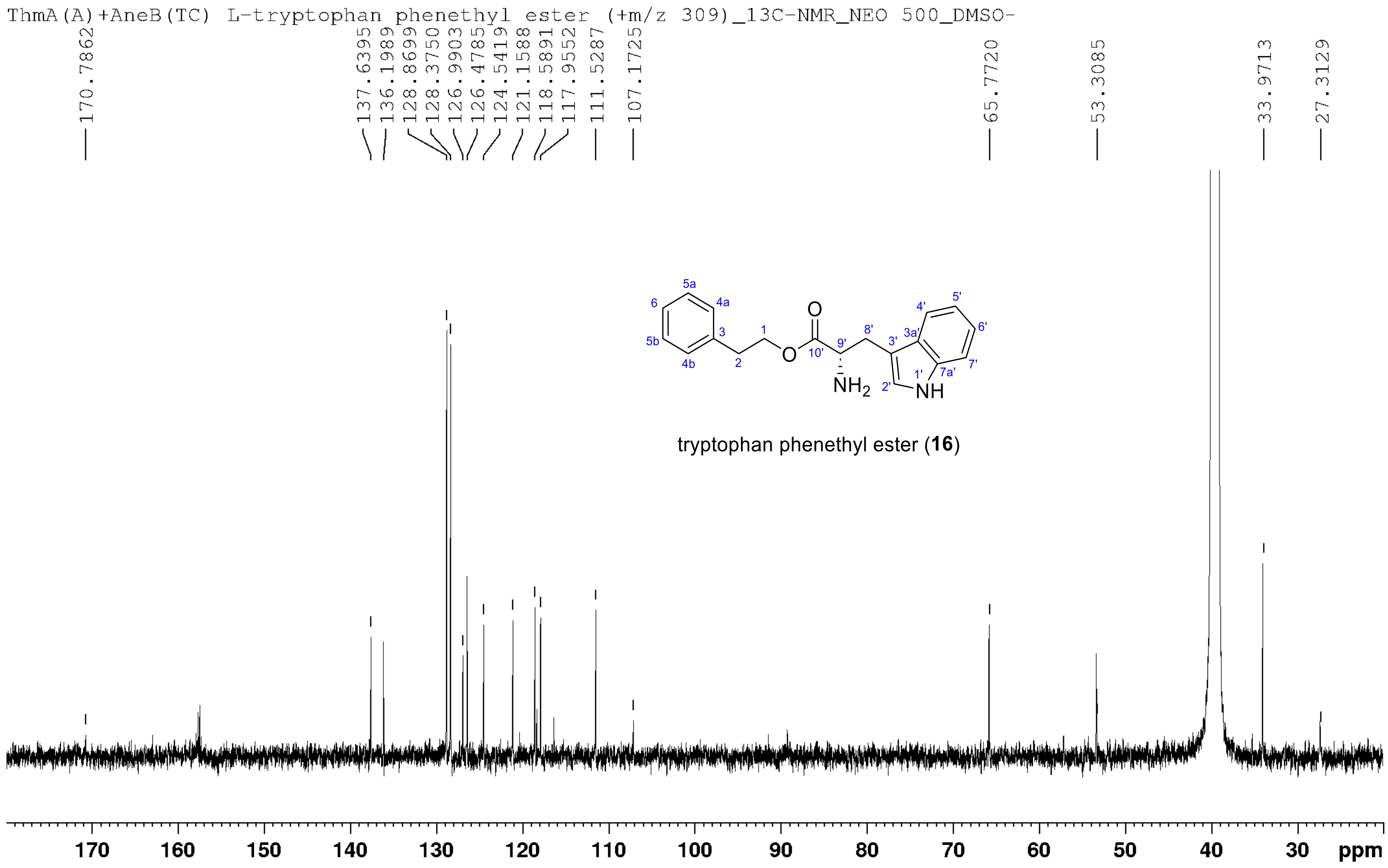

Fig. S23. ^{13}C NMR spectrum (DMSO-d_6, 125 MHz) of **16**.

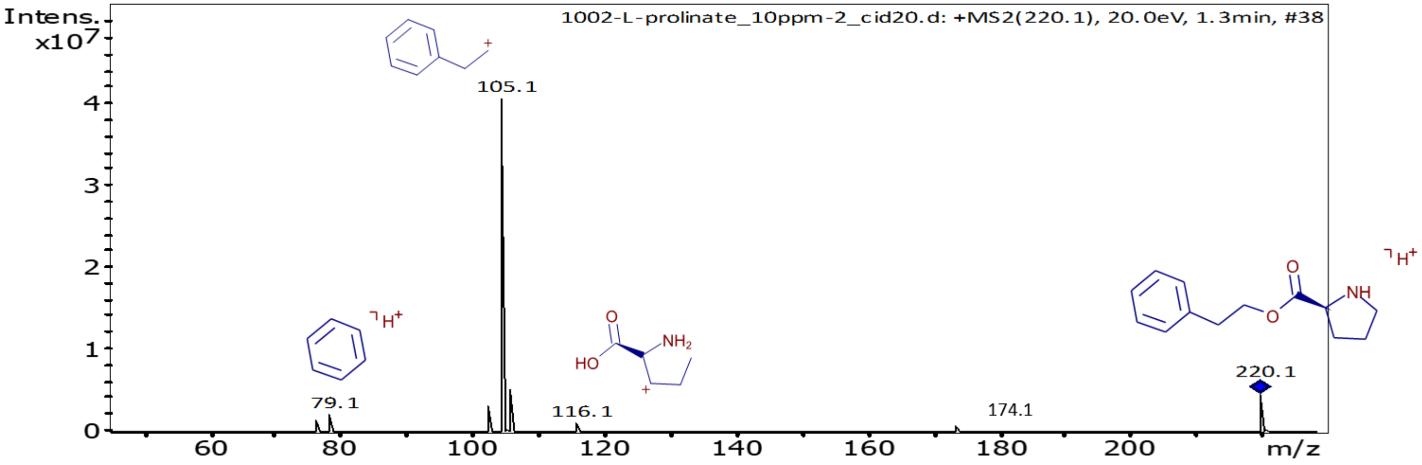

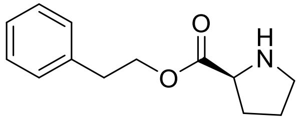

proline phenethyl ester (**10**)

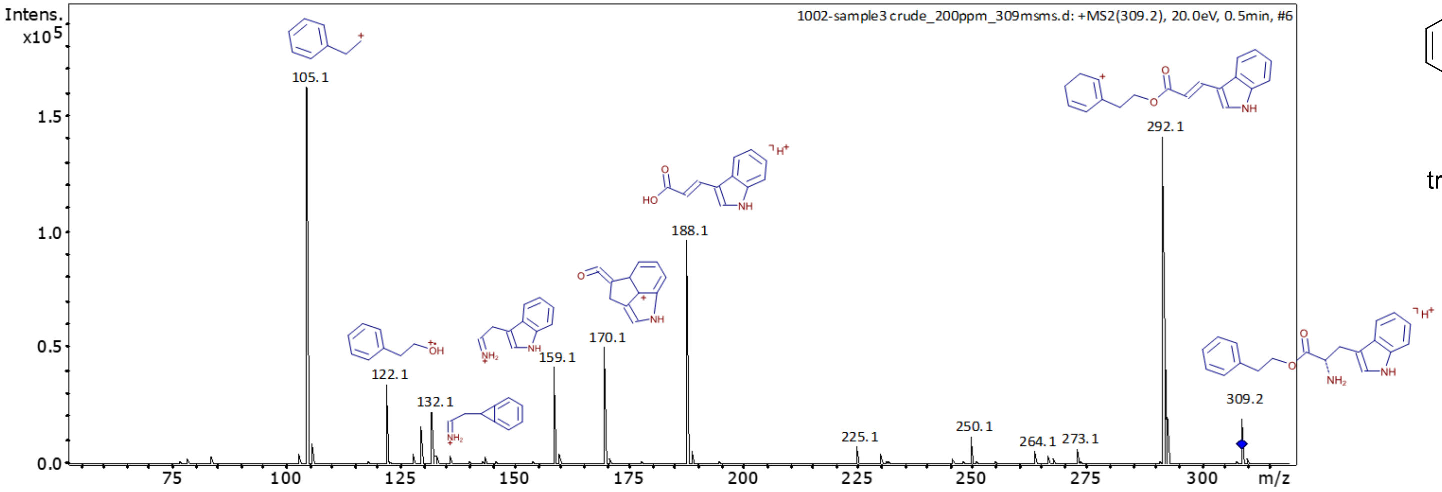

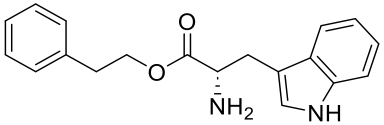

tryptophan phenethyl ester (**16**)

Fig. S24. MS/MS spectra of **10** and **16**.

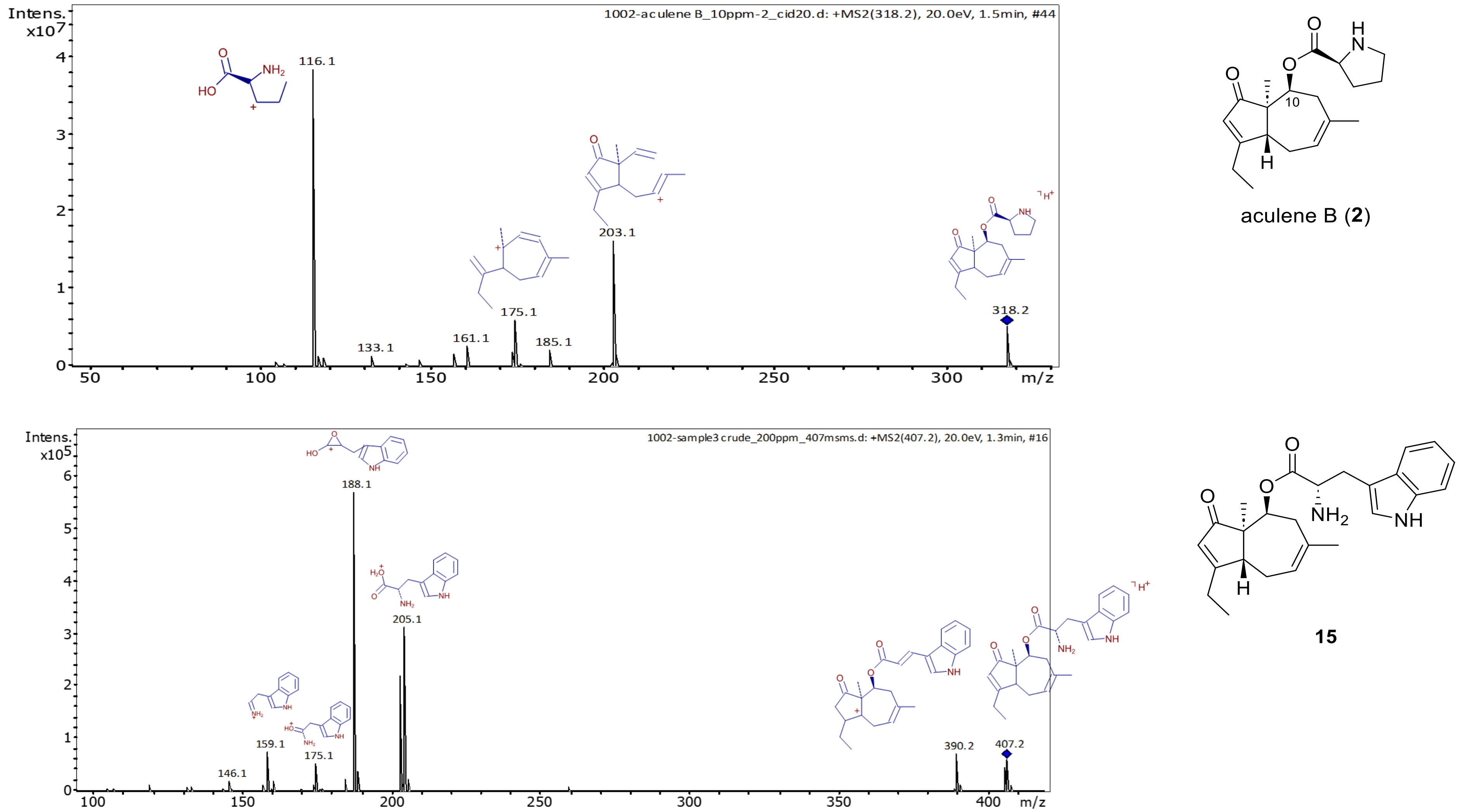

Fig. S25. MS/MS spectra of **2** and **15**.

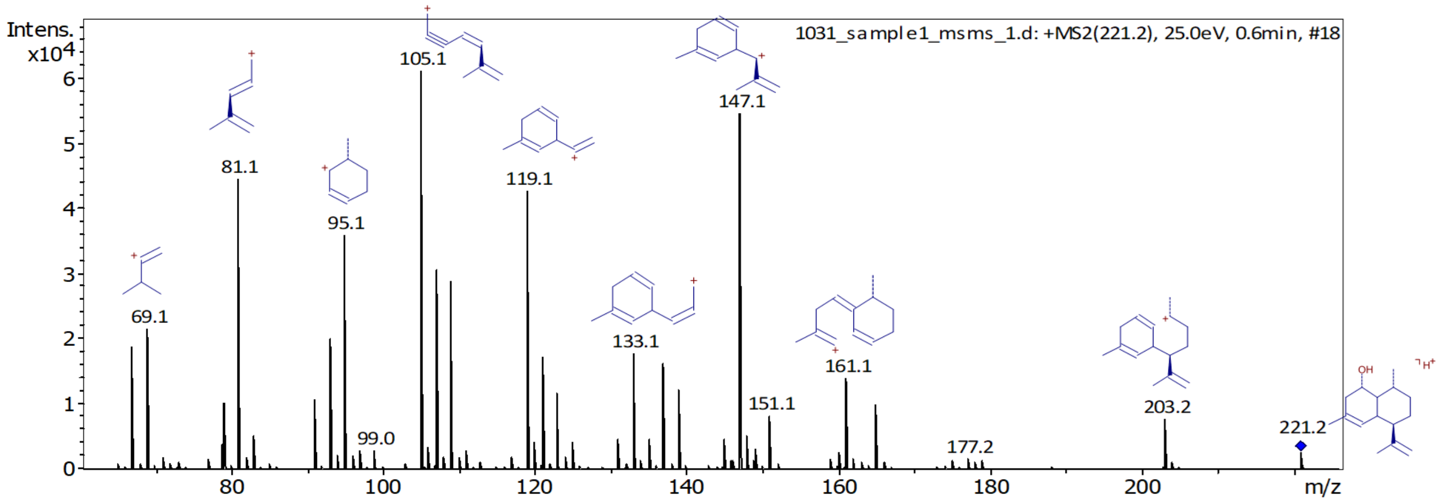

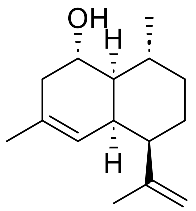

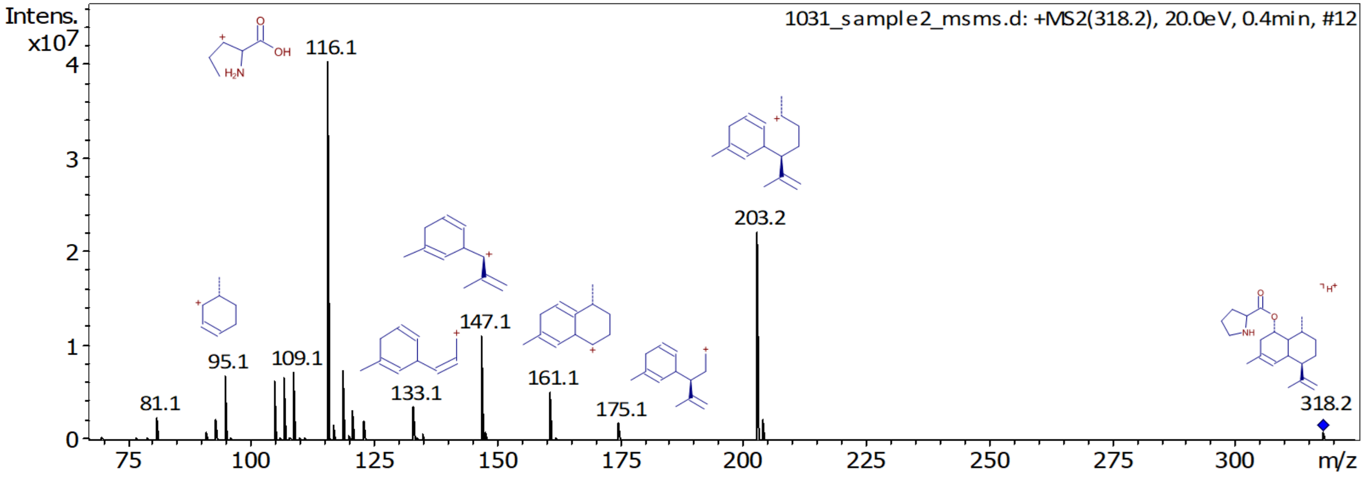

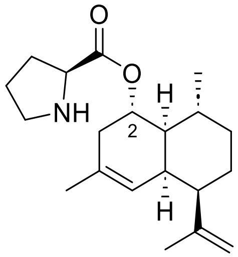

Fig. S26. MS/MS spectra of **12** and **13**.

Asperaculane H (**6**), HRMS (ESI) *m/z*: $[M+H]^+$ Calcd for $C_{19}H_{28}NO_4$ *m/z* 334.20128, Found 334.2004

Spectrum

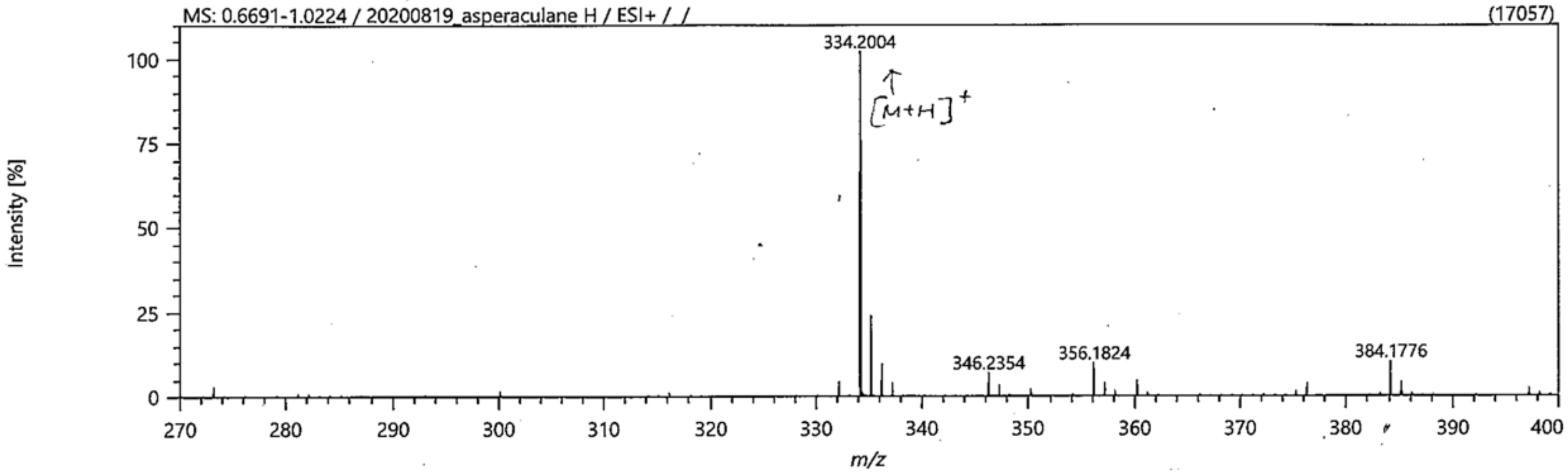

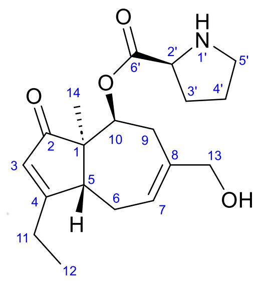

asperaculane H (**6**)

Elemental Composition

Parameters

Tolerance:	±10.00 ppm
Electron:	Odd/Even
Charge:	+1
DBE:	-99.0 - 999.0

Elements Set 1:

Symbol	C	H	N	O
Min	0	0	1	4
Max	1000	4000	1	4

Results

Mass	Formula	Calculated Mass	Mass Difference [mDa]	Mass Difference [ppm]	DBE
334.20041	C19 H28 N O4	334.20128	-0.87	-2.61	6.5

Fig. S27. HRMS spectrum of asperaculane H (**6**).

Compound	MS	UV

$[M+H]^+ = 316$, $[M+Na]^+ = 338$

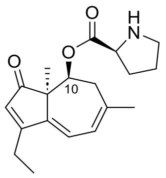

aculene A (**1**)

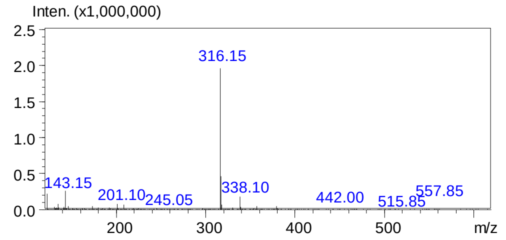

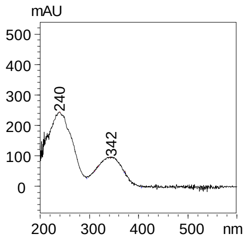

$[M+H]^+ = 318$, $[M+Na]^+ = 340$

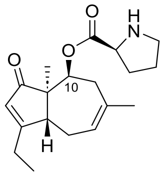

aculene B (**2**)

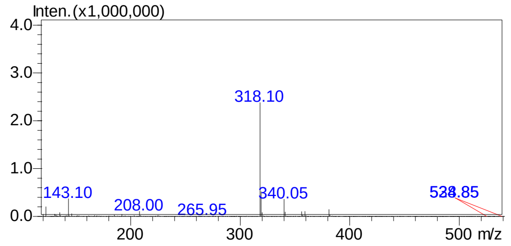

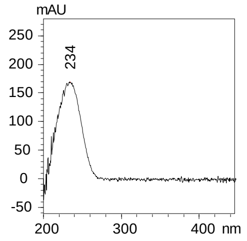

$[M+H]^+ = 219$, $[M+H-H_2O]^+ = 201$

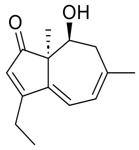

aculene C (**3**)

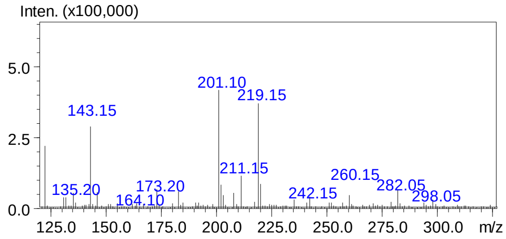

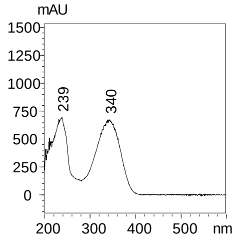

$[M+H]^+ = 221$, $[M+Na]^+ = 243$, $[M+H-H_2O]^+ = 203$

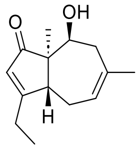

aculene D (**4**)

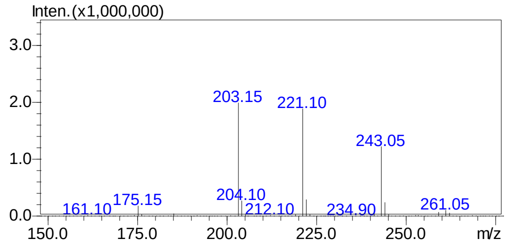

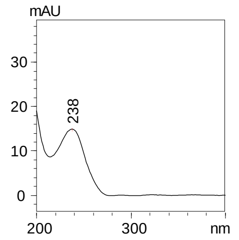

$[M+H]^+ = 237$, $[M+Na]^+ = 259$,

$[M+H-H_2O]^+ = 219$, $[M+H-2H_2O]^+ = 201$

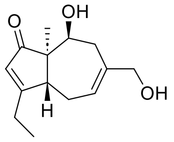

asperaculane B (**5**)

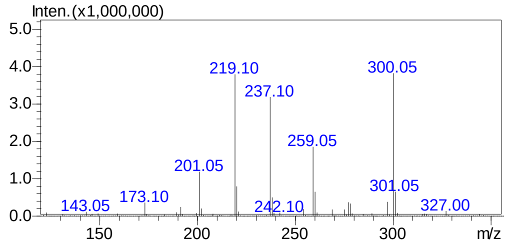

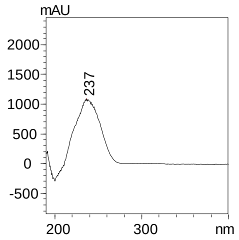

Fig. S28. UV and MS spectra of **1–5**. MS spectra are at positive mode.

Compound	MS	UV

$[M+H]^+ = 334$, $[M+H+ACN]^+ = 375$

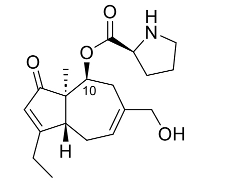

asperaculane H (**6**)

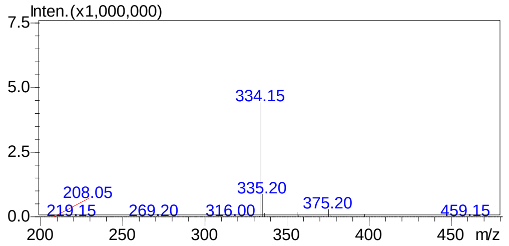

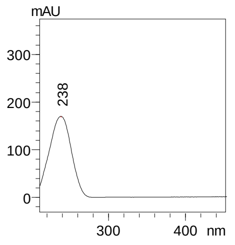

$[M+H]^+ = 233$, $[M+H+ACN]^+ = 274$

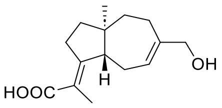

asperaculane C (**7**)

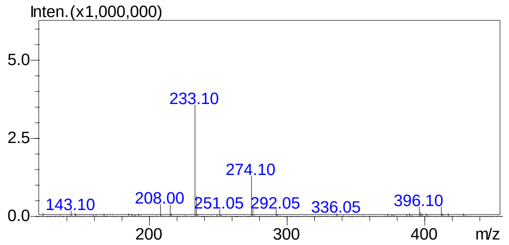

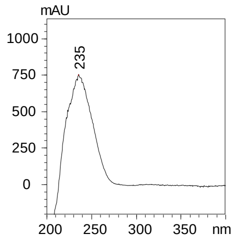

$[M+H]^+ = 348$, $[M+H+ACN]^+ = 389$

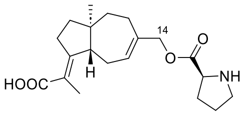

14-prolyl asperaculene C (**8**)

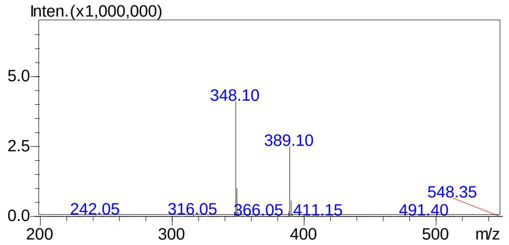

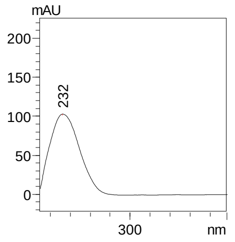

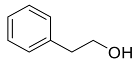

2-phenylethanol (**9**)

N/A

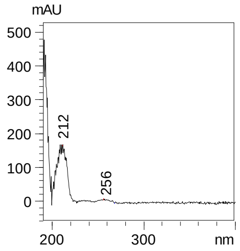

$[M+H]^+ = 220$, $[M+H+ACN]^+ = 261$

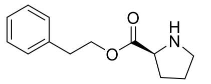

proline phenethyl ester (**10**)

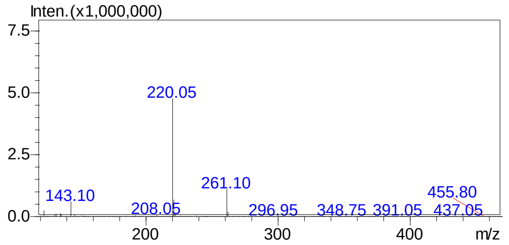

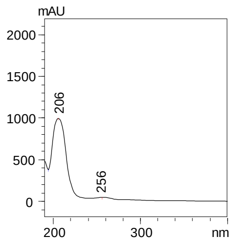

Fig. S29. UV and MS spectra of **6−10**. MS spectra are at positive mode.

Compound	MS	UV
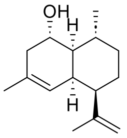 amorpha-4,11-diene-2-ol (**12**)	$[M+H-H_2O]^+ = 203$ 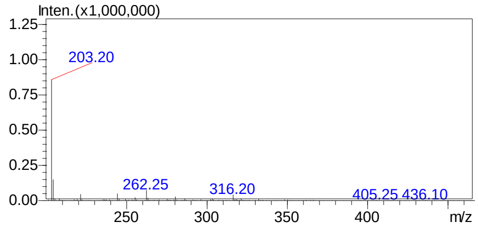	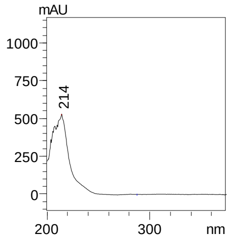
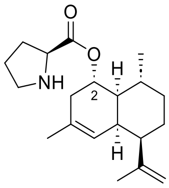 **13**	$[M+H]^+ = 318$, $[M+H+ACN]^+ = 359$ 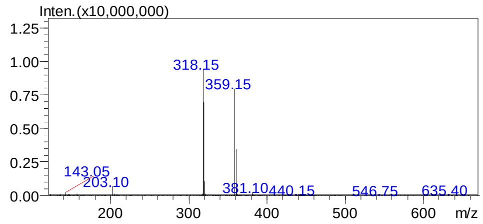	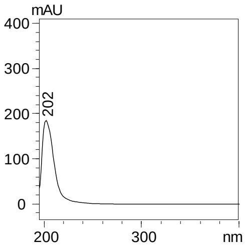
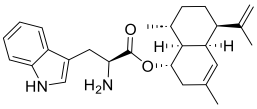 **14**	$[M+H]^+ = 407$ 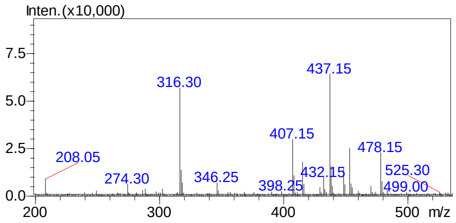	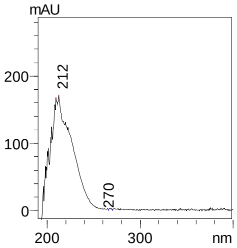
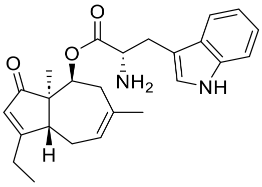 **15**	$[M+H]^+ = 407$, $[M+Na]^+ = 429$ 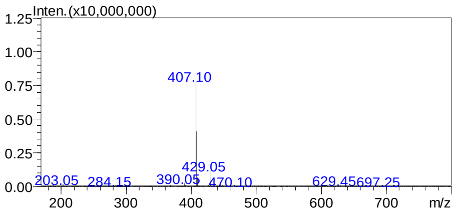	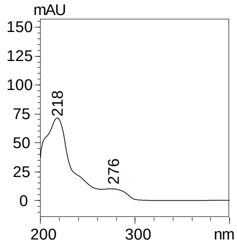
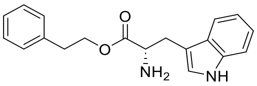 tryptophan phenethyl ester (**16**)	$[M+H]^+ = 309$, $[M+H+ACN]^+ = 350$ 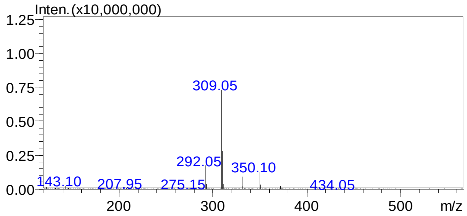	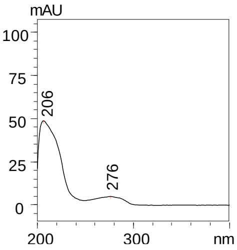

Fig. S30. UV and MS spectra of **12−16**. MS spectra are at positive mode.

References

1. Ngo, K.-S.; Brown, G. D., Synthesis of amorphane and cadinane sesquiterpenes from fabiana imbricata. *Tetrahedron* **1999**, *55* (52), 15099-15108.
2. Cheng, W.; Chen, M.; Ohashi, M.; Tang, Y., Biosynthesis of Terpenoid–Pyrrolobenzoxazine Hybrid Natural Product CJ-12662. *Angewandte Chemie International Edition* **2022**, *61* (12), e202116928.
3. Scott, D. E.; Coyne, A. G.; Venkitaraman, A.; Blundell, T. L.; Abell, C.; Hyvönen, M., Small-Molecule Inhibitors That Target Protein–Protein Interactions in the RAD51 Family of Recombinases. *ChemMedChem* **2015**, *10* (2), 296-303.
